# Substitution-Mutation Rate Ratio (c/µ) As Molecular Adaptation Test Beyond Ka/Ks: A SARS-COV-2 Case Study

**DOI:** 10.1007/s00239-025-10248-6

**Published:** 2025-05-03

**Authors:** Chun Wu, Nicholas J. Paradis, Khushi Jain

**Affiliations:** 1 Department of Chemistry and Biochemistry, Rowan University, Glassboro, NJ 08028 USA; 2 Department of Biological & Biomedical Sciences, Rowan University, Glassboro, NJ 08028 USA

**Keywords:** c/u, Ks/u, Ka/Ks, Translated Region, UnTranslated Region, SARS-COV-2, Adaptive mutations

## Abstract

The Ka/Ks ratio test, which assesses nonsynonymous versus synonymous substitution rates in Translated Region (TR) of a genome, is widely used to quantify fitness changes due to mutations but its critical limits are to be addressed. Ka/Ks can categorize the total fitness change as neutral (Ka/Ks = 1), beneficial (Ka/Ks > 1), or deleterious (Ka/Ks < 1), only if synonymous mutations are neutral. Otherwise, Ka/Ks only provides the fitness change due to protein sequence change. This neutrality assumption also renders this test inapplicable to sites in non-protein-coding UnTranslated Region (UTR). Our previous work introduced a substitution-mutation rate ratio (c/µ) per nucleotide site test (c: substitution rate in UTR/TR or a mean value of Ka and Ks in TR; and µ: mutation rate) as a generalized alternative to detect selection pressure, offering a broader application without forementioned presumptions. This paper derives a general equation linking c/µ with weighted Ks/µ and Ka/µ (c/µ = Ps*(Ks/μ) + Pa*(Ka/μ), Ps and Pa: proportions of synonymous and nonsynonymous sites under a mutation model and a codon table), demonstrating that Ka/Ks infers the same fitness change as c/µ does only if synonymous mutations are neutral (i.e. Ks/µ = 1). Otherwise, Ka/Ks might provide a different assignment from the c/µ test. Indeed, our comparative analysis of the c/µ and Ka/Ks tests across 25 proteins of SARS-COV-2 using three independent genomic sequence datasets shows that Ka/Ks inaccurately reports the type of fitness change for 7 proteins. Our findings advocate for the c/µ test to complement traditional Ka/Ks test to detect the selection pressure at a nucleotide site in a genome.

**Supplementary Information:**

The online version contains supplementary material available at 10.1007/s00239-025-10248-6.

## Introduction

In evolutionary biology, continuous-time Markov models describe sequence evolution as a stochastic process influenced by mutation, selection, and genetic drift, where state transitions (e.g., nucleotide, codon or amino acid changes) occur continuously over time (Anisimova and Kosiol 2009). Two fundamental frameworks, built on continuous-time Markov models, are central to measuring the mode and strength of selection in protein-coding sequences (Spielman and Wilke 2015). The well-known Ka/Ks ratio framework (Ka: nonsynonymous amino acid-changing substitution rate, Ks: synonymous substitution rate) is commonly used to detect molecular adaptation of nonsynonymous nucleotide (NT) mutations in the Translated Region (TR) of a genome, leading to amino-acid change in protein sequence (i.e. the fitness change is neutral if Ka/Ks = 1, beneficial if Ka/Ks > 1 or deleterious if Ka/Ks < 1) (Nei and Gojobori 1986) (Li et al. 1985). Although Ka/Ks is powerful to reflect selective pressure on protein sequence evolution (Echave et al. 2016) and adaptation (Moutinho et al. 2019), its validity on probing the total fitness beyond the protein level requires a critical assumption that synonymous mutations are effectively neutral (Ks = µ = constant) (Kimura 1977). This assumption also implies that the Ka/Ks test is not applicable to the non-protein-coding UnTranslated Regions (UTRs) of the genome as they are assumed to be neutral from a protein-centric viewpoint. Yet, from a nucleic-acid-centric view, mounting evidence suggests synonymous sites in TR and regulation sites in UTR can be under strong natural selection due to their critical roles in transcription and translation of DNA/RNA (Clarke 1970; Minchin and Lodge 2019) and their influence on protein folding and drug binding (Kimchi-Sarfaty et al. 2007). Furthermore, the Ka/Ks test faces a paradox: estimating Ka and Ks requires a predetermined mutation model and genetic codon table for determining the proportions of nonsynonymous and synonymous sites (Pa and Ps) in TR. Because the NT mutation model can more likely be shaped by selection, its parameter assumption on frequency and transition rates can lead to potential bias in assessing selection pressure (Latrille and Lartillot 2022). For example, common codon usage for optimizing protein translation is reported for fast-growing organisms (viruses, yeast, bacteria, Drosophila) (Tian et al. 2018), therefore using mutation models with equal probabilities of codon usage would lead to the wrong assessment. In contrast, the genetic codon table is probably not heavily shaped by selection, as the standard codon table is shared by most organisms. Nonetheless, some species utilize non-standard genetic codes (Bezerra et al. 2015), often involving codon reassignments in their mitochondrial or nuclear genomes, demonstrating the genetic code's adaptability to specific organismal needs in very long evolution time-scales.

Another critical framework is the mutation-selection (MutSel) models which are firmly grounded in population genetics theory (Fisher 1931; Kimura 1962; Wright 1931). While Ka/Ks offers a comparative measure of selection pressure (Eyre-Walker 2006; Yang and Bielawski 2000), MutSel provides a mechanistic explanation of sequence evolution under mutation and selection (Teufel et al. 2018). In details, MutSel assesses the selection strength at individual NT sites or codons by estimating their scaled selection coefficients ($S=2{N}_{e}s$) (S < 0 if purifying, S = 0 if strictly neutral and S > 0 if beneficial) (Halpern and Bruno 1998), where N_e_ is the effective population size and s is the selection coefficient acting on a codon site. MutSel models incorporate the detailed balanced condition of the substitution process (${c}_{ij}{\pi }_{i}={c}_{ji}{\pi }_{j}$) (Halpern and Bruno 1998), assuming that the observed substitution rates ${c}_{ij}$ and ${c}_{ji}$ are time-independent, and codon frequencies ${\pi }_{i}$ and ${\pi }_{j}$ at long time limit have reached stationary/equilibrium state, to obtain the scaled selection coefficient for a site at equilibrium (${S}_{ij}=\text{ln}(\frac{{\mu }_{ji}{\pi }_{j}}{{{\mu }_{ij}\pi }_{i}}$)) where ${\mu }_{ij}$ and ${\mu }_{ji}$ are time-independent forward and backward NT mutation rates between codon *i* and *j* (Halpern and Bruno 1998). Although MutSel models are considered as alternatives to Ka/Ks in probing the site-specific selection pressure acting on both nonsynonymous and synonymous mutation sites in TR, obtaining the stationary distribution of the genotypes in the population from empirical sequence data is challenging, requires intensive computation, data and resource requirements [2], limiting its potential use when compared to Ka/Ks. Lastly, MutSel per codon framework has not yet been widely extended to study the selection pressure in UTR at a NT level.

Interestingly, Spielman et al. have established mathematical links between the two frameworks [2] by deriving equations to obtain Ka (dN) and Ks (dS) for a codon site in TR using µ_ij,_ c_ij_, and π_i_ (i.e., m_ij_, q_ij_ and P_i_ respectively in Eqs. 8 and 9 of the original paper: $dN=\frac{{K}_{N}}{{L}_{N}}=\frac{\sum_{i}\sum_{j\epsilon {\text{\rm N}}_{i}}{\pi }_{i}{c}_{ij}}{\sum_{i}\sum_{j\epsilon {\text{\rm N}}_{i}}{\pi }_{i}{\mu }_{ij}}$ and $dS=\frac{{K}_{S}}{{L}_{S}}=\frac{\sum_{i}\sum_{j\epsilon {\text{S}}_{i}}{\pi }_{i}{c}_{ij}}{\sum_{i}\sum_{j\epsilon {\text{S}}_{i}}{\pi }_{i}{\mu }_{ij}}$, where *S*_i_ is the set of codons that are synonymous to codon *i* and differ from it by one NT substitution; *Ln* and *Ls* are the number of nonsynonymous and synonymous sites according to the mutational opportunity definition of a site) (Spielman and Wilke 2015). Using this relationship, they revealed the critical limits of these two frameworks, being the Ka/Ks values of codon sites when Ks = µ was assumed or not; when assumed, Ka/Ks ≤ 1 for all observed codon sites; when not assumed, Ka/Ks can take on arbitrarily high values even if all selection is purifying. Thus, the Halpern-Bruno MutSel does not inherently accommodate positive diversifying selection due to its presumption of constant selection pressures over time (i.e. a static fitness landscape), whereas Ka/Ks cannot differentiate between purifying selection on synonymous codons and positive selection on amino acids due to its presumption of neutral synonymous sites. Clearly, there is a strong need to develop a novel unified framework that combines the strengths of the Ka/Ks and MutSel frameworks while also addressing their limitations.

**Fig. 1:**
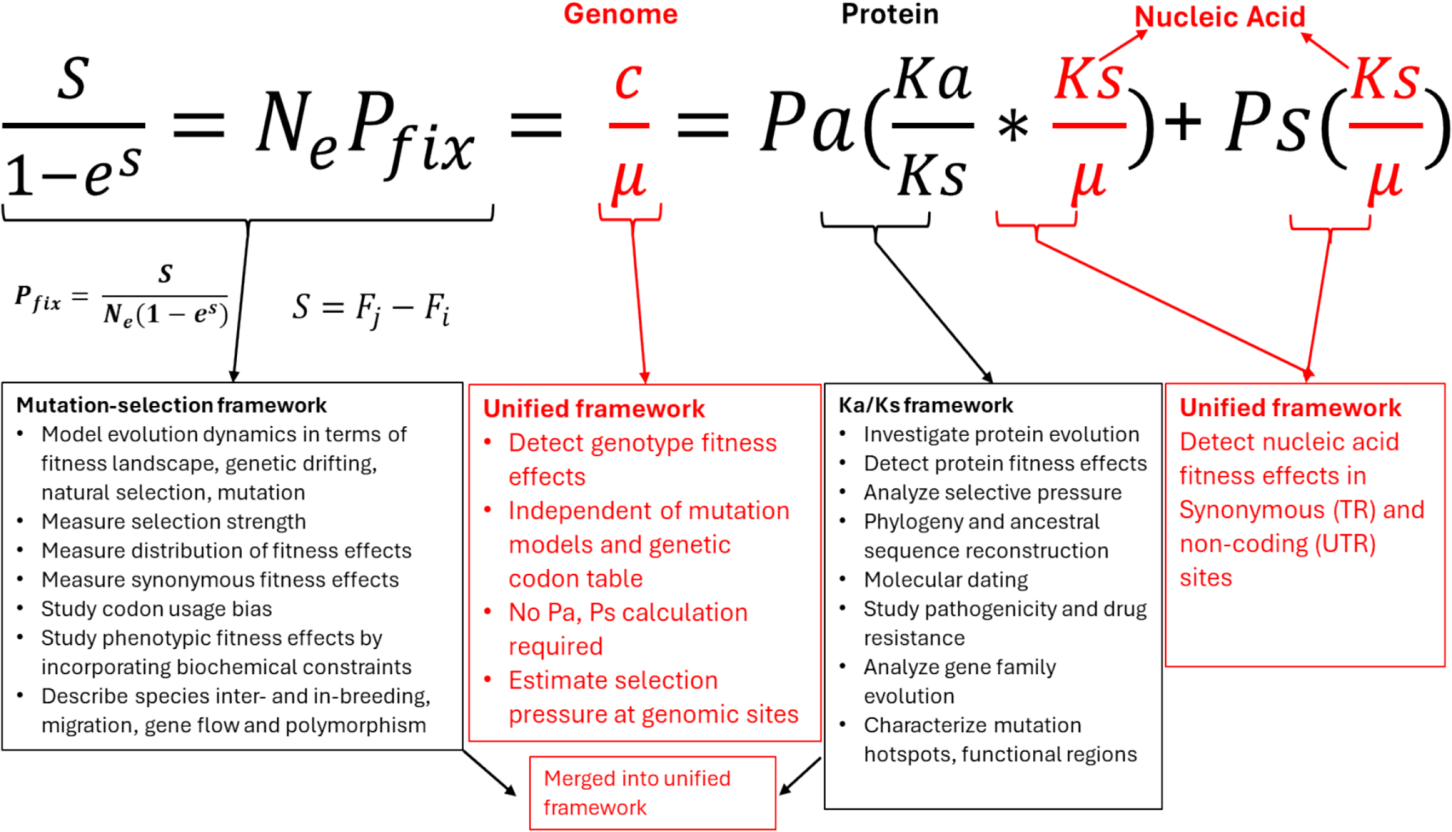
Integrating c/µ per NT site framework with MutSel per codon and Ka/Ks per gene frameworks to quantify the transient scaled selection coefficients (S) across different genome regions.* N*_*e*_ represents the effective haploid population size, *P*_*fix*_ denotes the probability of fixation, and the fitness of a NT mutant (${F}_{j}$) and wild type (${F}_{i}$) are provided

To address the critical limits harbored by the Ka/Ks per gene and MutSel per codon frameworks, we recently proposed a unified framework utilizing the substitution-mutation rate ratio (c/µ) test per NT site to quantify selection pressure on any genomic region, including NT sites in UTR and synonymous and nonsynonymous NT sites in TR, using empirical sequence data (Fig. 1). The foundation of our c/µ framework lies in our replication-selection model (Wu et al. 2023), which assumes the mutation and selection steps are independent from each other as required in MutSel (Teufel et al. 2018); it calculates the substitution rate (c) as a product of the effective population size with the estimated mutation rate (µ) and the fixing probability (P_fix_) from sequence data. The resulting c/µ ratio can serve as a test to reflect the selection pressure on mutants at a NT site in the population surviving selection (beneficial if c/µ > 1, neutral if c/µ = 1 and purifying if c/µ < 1). The c/µ framework has several distinct advantages over the Ka/Ks and MutSel frameworks. **First**, our c/μ framework offers a simple way to calculate transient scaled selection coefficients using c/µ without assuming constant selection pressures, requiring stationary base frequencies (π_i_) or the phenotype fitness of a mutant (${F}_{j}$) and wild type (${F}_{i}$) from MutSel. The transient scaled selection coefficients can provide useful insight of evolution dynamics for rapidly evolving species populations across short timespans. **Second**, the c/µ ratio can detect fitness changes due to mutations in TR and UTR, similarly to the Ka/Ks ratio for TR, but without assuming that synonymous sites are neutral, enabling unbiased observation of the fitness effects acting on synonymous sites in TR and NT sites in UTR. Thus, while Ka/Ks obtains the fitness effects from protein sequence change, c/µ can obtain the complete fitness effects from nucleic acid sequence change including nonsynonymous Ka/µ and synonymous Ks/µ (see further below). **Third**, c/µ does not require the use of a predetermined mutation model and genetic codon table, providing a more robust, unbiased assessment of the total fitness effects and their roles in nucleic acid evolution. **Fourth**, contrary to Ka/Ks, the c/µ test enables the highest-resolution analysis of substitution timelines, site substitution-mutation rate ratio spectra (SSMRRS), distribution of fitness effects (DFE) and fraction of mutation types within a short sampling time. This advantage allows c/µ to assess the selection forces shaping the molecular clock feature, reliably identify adaptive mutations in both TR and UTR and predict the selection pressures from mutation acting at each NT site and segment. The full assessment of c/µ against the Ka/Ks and MutSel frameworks in probing mutation-selection effects is beyond the scope of this paper (a separate paper is under preparation).

Here, we derived a general equation (c/µ = Ps*(Ks/μ) + Pa* (Ka/μ) and Ps + Pa = 1) explicitly linking three relative substitution rates c/µ, Ka/µ, Ks/µ, showing that while c/µ is always true to quantify the fitness change of any NT mutation, Ka/Ks per codon is equivalent to c/µ for codon mutations only when the assumption of synonymous sites (Ks/µ = 1) is true. If this assumption is false (Ks/µ ≠ 1), Ka/Ks could provide different assignments than c/µ because it only provides the fitness change due to protein sequence change (Yang and Bielawski 2000), whereas c/µ provides the fitness change due to nucleic acid sequence change (Ks/µ and Ka/µ) (Clarke 1970). Furthermore, when synonymous sites are under purifying selection (Ks≈0), c/µ is numerically more robust than Ka/Ks for these sites because the mutation rate (µ) is a well-defined non-zero number, and c/µ, Ks/µ and Ka/µ can be defined at these sites; in contrast, Ka/Ks is not well-defined at these sites and can produce false positive signals. Indeed, the difficulty of Ka/Ks is escalated when the sequence length is short or a site-by-site analysis is studied (Pond and Frost 2005). More importantly, this general equation is also applicable to UTR where Ps = 1 and Pa = 0.

Although the c/µ analysis is applicable to all organisms in principle, viruses are the focus of our initial development, validation and optimization due to urgent public health needs and their relatively simple genomic replication mechanism that might lead to a site-independent time-independent µ in a short time scale such as months and years. In our first study, c/µ analysis of a SARS-COV-2 genomic dataset comprising three independent sets totaling 11,198 genome sequences collected over the first 19 months of the pandemic, inferred the proportions of different mutation types (86% strongly deleterious, 7% weakly deleterious, 0.5% strictly neutral, 3% weakly beneficial, 4% strongly beneficial) for the first time from an L-shaped c/μ DFE despite exhibiting a genomic substitution timeline under a constant rate (R^2^ = 0.9833) and large fluctuations in human infection cases and increasing vaccination rates. This L-shaped DFE was inconsistent with those predicted by mainstream evolution theories (Genomic Substitution Rate Model/GSRM, Neo-Darwinist Selectionist Theory/ST, Kimura’s Neutral Theory/KNT and Ohta’s Nearly Neutral Theory/ONNT), but was consistent with our Nearly Neutral Balanced Selectionist Theory (NNBST), which proposes the constant substitution rate for a genomic segment, perceived to be under effective neutral selection rather than strictly neutral selection, is produced by the balancing effect of slower substitution rates under deleterious selection with faster substitution rates under beneficial selection (Wu et al. 2023). In our second study, we refine our estimate of the lower boundary of µ using NNBST and show that combined methodologies can detect mutation sites under adaptive selection for nucleotide sites in UTR (c/µ > 3), nonsynonymous sites in TR (c/µ > 3 and Ka/Ks > 2.5) and synonymous sites in TR (c/µ > 3 and Ks/µ > 3), with most identified UTR and nonsynonymous mutation sites showing good consistency with literature-reported adaptive mutation effects. These literature-validated mutation sites aid in optimizing the upper boundary of the true µ value at least for SARS-COV-2 (Paradis and Wu 2024).

In this study, we continue our assessment of applying the c/µ framework to SARS-COV-2 using the same genomic dataset. We compare our in vivo µ with in vitro cell-based and cell-free µ values in the literature to show its consistency; and then we provide a detailed assessment of our c/µ framework against the Ka/Ks framework in probing the fitness effects at segment/gene level using the derived general equation. In detail, comparison between c/µ and Ka/Ks for detecting molecular adaptation of 10 genes (25 proteins), 11 UTRs and 9 TRSs (Transcriptional Regulatory Sequences) was carried out. Indeed, all UTRs and TRSs except for Orf1ab 5’UTR are not effectively neutral. Furthermore, none of the 25 proteins exhibited Ks/µ = 1, satisfying the equivalency condition between c/µ and Ka/Ks. This suggests that most synonymous mutations in these proteins are not effectively neutral. Although c/µ and Ka/Ks report the same type of fitness change for 18 out of the 25 proteins in TR, Ka/Ks reports a different type of fitness change for the 7 proteins from the c/µ assignment by missing the fitness change due to synonymous mutations (Ks/µ).

## Methods

Our previous paper (Wu et al. 2023) used a replication-selection model (Figure S1) to describe the molecular evolution of a virus, defining the substitution-mutation rate ratio (c/µ) to quantify fitness changes due to mutations. In this paper, we provide a concise description of the model and focus on deriving the general equation linking c/µ, Ka/µ, and Ks/µ. We demonstrate that while c/µ consistently quantifies fitness change, Ka/Ks is equivalent to c/µ only when the effective neutrality assumption of synonymous sites holds true (Ks/µ = 1). Refer to Table 1 for definitions of major symbols presented throughout this text.

**Table 1:** Definitions of Major symbols

Symbol	Definition
n_c_/Nc	Census Population of a virus
Ne	Replicated Population of a virus before the selection
G	Number of genomes
t	Collecting date of a genome
Δt	Δt = t_max_-t_min_
m_ij_	Substitution count matrix (Time, Position)
P_mut_	Mutation probability
P_fix_	Fixation/substitution probability
µ	Number of mutations per NT site per unit time; mutation rate
c	Number of substitutions per NT site per unit time; substitution rate
c/µ	Substitution/mutation rate ratio; relative substitution rate
S	Number of synonymous NT sites in a sequence
A	Number of nonsynonymous NT sites in a sequence
N	Number of NT sites in the sequence (N = S + A)
Ρs	Proportion of synonymous sites (Ps = S/(S + A))
Ρa	Proportion of nonsynonymous sites (Pa = 1-Ps = A/(S + A))
Ts	Total number of synonymous substitutions per unit
Ta	Total number of nonsynonymous substitutions per unit
Tn	Total number of substitutions per unit (Tn = Ts + Ta)
Ks	Number of synonymous substitutions per synonymous NT site per unit time; synonymous substitution rate (Ks = Ts/S)
Ka	Number of nonsynonymous substitutions per nonsynonymous NT site per unit time; nonsynonymous substitution rate (Ka = Ta/A)
Ks/µ	Synonymous substitution/mutation rate ratio; relative synonymous substitution rate
Ka/µ	Nonsynonymous substitution/mutation rate ratio; relative nonsynonymous substitution rate
Ka/Ks	Nonsynonymous/synonymous rate ratio
NT	NT
AA	Amino Acid
TR	Translated Region
UTR	UnTranslated Region
TRS	Transcriptional Regulatory Sequence

### The Replication-Selection Model for Molecular Evolution: Quantifying Fitness Changes with Relative Substitution Rate for NT sites (c/µ), Nonsynonymous Sites (Ka/µ), and Synonymous Sites (Ks/µ)

**Fig. 2:**
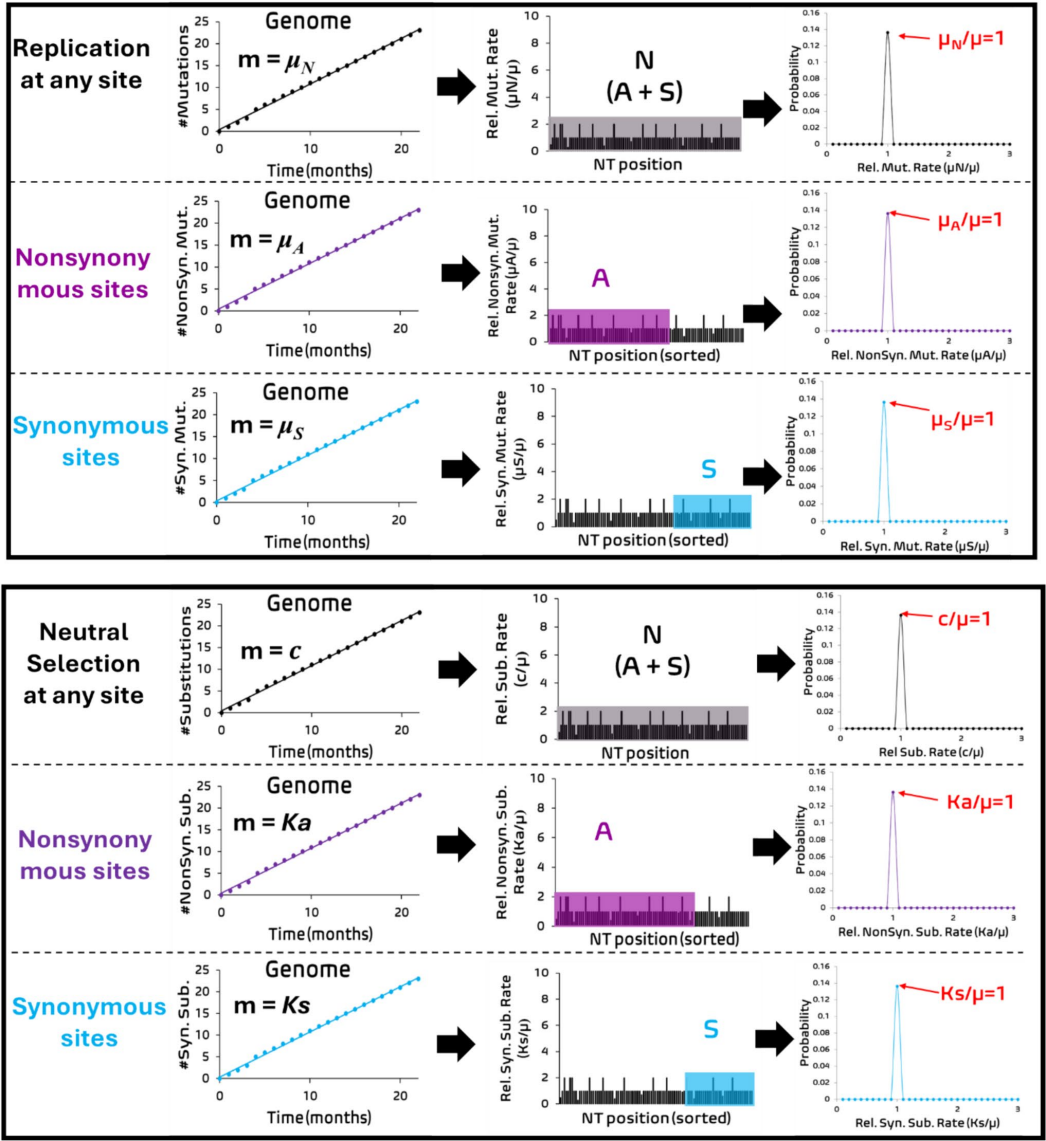
The viral genomic sequence under neutral selection. During replication, the viral polymerase induces copy errors at rate µ at each NT site in the copied viral sequence strand. The total NT sequence length (N) is the sum of nonsynonymous sites in the translated region (A) and synonymous sites in the sorted translated and untranslated regions (S) under a Markov mutation model and a codon table. Similarly, nonsynonymous and synonymous mutations are fixed into sites A and S at rates Ka and Ks, respectively. The resulting probability distribution of the relative substitution rate (c/μ), relative nonsynonymous substitution rate Ka (Ka/μ) and relative synonymous substitution rate Ks (Ks/μ) for a given NT site under neutral selection is described as a Poisson distribution curve due to sampling error

A replication-selection model (Figure S1) was introduced to describe the evolution of a virus (Wu et al. 2023). To define µ, three prior assumptions are made in the replication step of the model (Fig. 2). In the first assumption, mutations occur independently from the selection process over short time periods such as months or years, and the probability of mutation introduction (P_mut_) is quantifiable by in vitro cell-based or cell-free test of the polymerase machinery including its proofreading component. Hence, the substitution rate (c) is determined by multiplying the introduced mutations in the population (Ne*µ) with its fixation probability under selection (P_fix_). In the second assumption, the copy error from the polymerase ± proofreader machinery occurs without preference for a particular NT site regardless of its roles in the selection such as nonsynonymous or synonymous sites in TR or UTR, and this copy error occurs with a phenomenological rate (i.e. μ_N_ = μ_A_ = μ_S_ = μ). In the third assumption, mutations in the polymerase machinery do not significantly change its structure in the short time scale (e.g. months), thereby µ does not significantly change over time. These assumptions appear to be justified by the low mutation rates observed in viruses (10^–3^-10^–8^ mutations per nucleotide site per year). While µ could change over longer time periods or across different viral lineages, the time-independence of µ can be relaxed to accommodate these effects but is beyond the scope of this study. These assumptions have been stated in numerous mutation models (Kimura and Crow 1964; Teufel et al. 2018), although they remain to be experimentally verified. It is worthwhile to note the phenomenological constancy of µ can be consistent with a set of Markov mutation models ranging from one parameter to nine parameters. (Jukes and Cantor 1969; Tavaré 1986; Kimura 1980; Hasegawa et al. 1985). However, a Markov mutation model and a codon table is required to determine synonymous and nonsynonymous sites in a coding sequence for the Ka/Ks method.

The total substitution rate of a NT site (c) including contributions from the nonsynonymous substitution rate (Ka) and the synonymous substitution rate (Ks), is defined as the product of µ and c/µ for that NT site, including either the nonsynonymous substitution-mutation rate ratio (Ka/µ) or synonymous substitution-mutation rate ratio (Ks/µ), reflecting different selection pressures at the site. Here, the following assumption can be made: all NT sites, including either all nonsynonymous NT sites or all synonymous NT sites, undergo neutral selection, which can be described from several equivalent models (e.g., Genomic Substitution Rate Model/GSRM (Wu et al. 2023) or the Strict neutrality hypothesis (de Jong et al. 2024)). If the assumption is satisfied, then the observed mutation rate at any NT site *j* (with a substitution rate, c_j_) including either the observed nonsynonymous substitution rate (Ka_j_) at any nonsynonymous NT site or the observed synonymous substitution rate (Ks_j_) at any synonymous NT site in the viral population, is equal to the spontaneous mutation rate in individual organisms (c_j_ = Ka_j_ = Ks_j_ = µ, shown in Fig. 2) (Gojobori et al. 1990; Kimura 1968). Therefore, the substitution rate for all nonsynonymous NT sites (A) in TR or all synonymous NT sites (S) in TR and UTR, genetic and sub-genetic segments in TR and non-coding UTR/TRS segments is a constant and is equal to the spontaneous mutation rate (μ), leading to a global molecular clock (Bromham and Penny 2003). Together, the GSRM is characterized by three genetic hallmarks: a global molecular clock, a discrete uniform distribution of c/µ for each site in a genomic sequence, and a Poisson distribution centered at c/µ = 1 due to random sampling error, indicating neutral selection. The Segment Substitution Rate Model (SSRM) was introduced to allow for time-dependent natural selection pressure acting on the genome, thus causing the deviation of the (total, nonsynonymous or synonymous) substitution rate of a specific segment *j* from the constant mutation rate (i.e. the fitness change for the total, nonsynonymous or synonymous mutation is beneficial if c_j_/µ > 1, Ka_j_/µ > 1, or Ks_j_/µ > 1; deleterious if c_j_/µ < 1, Ka_j_/µ < 1, or  Ks_j_/µ < 1).

### The General Equation Linking c/µ, Ka/µ, and Ks/µ Showing c/µ is More General than the Ka/Ks Test to Infer Fitness Change Without the Neutrality Assumption of Synonymous Mutations

The genome length of *N* NTs consists of *i* functional segments (genes in TR and UTRs). We assume that spontaneous mutations are produced by the genomic replication system (mainly polymerase and proofreading enzymes) at any NT site with a single time-independent rate µ (i.e. mutation rate: the number of mutations per NT site per unit time). In other words, µ for each gene, UTR, or each NT site is the same constant over time, leading to a single global molecular clock (Bromham and Penny 2003).

Traditionally, synonymous sites (S) and nonsynonymous sites (A) of TR containing *N* NT sites are defined as the mutational opportunity, or the proportions of mutations that lead to expected synonymous and nonsynonymous mutations under a given Markov mutation model (Li 1993) and a genetic codon table for the studied species. Thus, S and A can be estimated by multiplying the TR sequence length (N) by the expected proportions of synonymous (Ps) and nonsynonymous (Pa) sites before undergoing selection (Ina). To calculate Ps and Pa, an appropriate Markovian mutation model (Nei and Jin 1989; Pamilo and Bianchi 1993) and genetic codon table for determining the NT mutation type (synonymous VS. nonsynonymous) must be chosen. A codon consists of three NTs, but 2- or 3-point NT mutations in a codon are very rare due to a low mutation rate. Practically, only 1-point NT mutations in a codon are considered for protein change (nonsynonymous NT mutation → amino acid replacement; synonymous NT mutation → no amino acid replacement) (Nei and Gojobori 1986) (Li et al. 1985). From 1-point mutations, this mutational opportunity (Pa and Ps) can be defined at each NT site, and it can be extended to UTRs, which are considered as “synonymous sites” (i.e., Ps = 1 and Pa = 0). This mutational opportunity definition hinges on the probability of the mutation affecting protein function and hence is good in studies of adaptive protein evolution (Bierne and Eyre-Walker 2003). However, this traditional definition can lead to a physical NT site with a certain chance to be synonymous (Ps) and the remaining chance to be nonsynonymous (Pa) simultaneously for the first position and third position of some codons. To remove those complexities, a more physical definition of synonymous sites was proposed to rely only on the genetic code for estimating the degeneracy at each codon in a given sequence (i.e., fourfold, threefold, and twofold degenerate sites) and only twofold (Bulmer 1991; Bulmer et al. 1991) or fourfold (Bierne and Eyre-Walker 2003) degenerate sites are defined as synonymous sites only (Ps = 1) to estimate the synonymous substitution rate (Ks). Nonetheless, the physical definition can be considered as a special case of the traditional definition. Following our assumption on the time-independent site-independent mutation rate (µ) in our replication-selection model, the ratio of expected synonymous mutations to nonsynonymous mutations for the whole TR (M) is equal to the ratio of the number of synonymous sites (S) to the number of nonsynonymous sites (A). Thus, our model follows the mutational opportunity definition (e.g. Ps/Pa = (S*µ) /(A*µ) and Ps + Pa = 1); and the derivation below is also true for each NT site (i.e. 1 = s + a, Ps/Pa = S/A):

1 $$N \, = S \, + \, A$$

2 $$Ps \, = {\raise0.7ex\hbox{$S$} \!\mathord{\left/ {\vphantom {S {\left( {S + A} \right)}}}\right.\kern-0pt} \!\lower0.7ex\hbox{${\left( {S + A} \right)}$}}$$

3 $$Pa \, = \, {\raise0.7ex\hbox{$A$} \!\mathord{\left/ {\vphantom {A {\left( {S \, + \, A} \right)}}}\right.\kern-0pt} \!\lower0.7ex\hbox{${\left( {S \, + \, A} \right)}$}} = \, 1 \, - \, Ps$$

S and A are required to calculate the number of synonymous substitutions per synonymous site per unit time (Ks) and the number of nonsynonymous substitutions per nonsynonymous site per unit time (Ka). Because the total number of substitutions is equal to a sum of synonymous substitutions and nonsynonymous substitutions based on the genetic code table,

4 $$Tn \, = \, Ta \, + \, Ts$$

The number of substitutions per NT site per unit time (c = Tn/N) can be obtained from weighted Ks (Ks = Ts/S) and Ka (Ka = Ta/A) parameters as follows:

5 $$c \, = \, Ps*Ks \, + \, Pa*Ka$$

If a spontaneous mutation within an individual is neutral, it will be fixed in its population at the same rate as its generation (i.e. c/µ = 1) at the mutation step. If the mutation change is deleterious, purifying selection will reduce its fixation rate (i.e. c/µ < 1). Only when the mutation offers a selective advantage will it be fixed at a higher rate (i.e. c/µ > 1). Regardless of whether a NT site is synonymous or nonsynonymous, its generation rate is the mutation rate (µ) at the mutation step preceding the selection step. Therefore, if a synonymous/nonsynonymous mutation within an individual is neutral, it will be fixed in its population at the same rate as its generation (i.e. Ks/µ = 1// Ka/µ = 1) at the mutation step. If the mutation change is deleterious, purifying selection will reduce its fixation rate (i.e. Ks/µ < 1// Ka/µ < 1). Only when the mutation offers a selective advantage, it will be fixed at a higher rate (i.e. Ks/µ > 1// Ka/µ > 1). To get these three ratios, Eq. (5) can be transformed into Eq. (6) by dividing both sides of Eq. (5) with μ:

6 $$\frac{c}{\mu } = Ps*\left( {\frac{Ks}{\mu }} \right) + Pa* \left( {\frac{Ka}{\mu }} \right)$$

Which implies that an overall fitness change due to mutations (c/µ) at a specific NT site is equal to the sum of a weighted fitness change due to synonymous mutations (Ks/µ) and a weighted fitness change due to nonsynonymous mutations (Ka/µ) at that site. The fitness of synonymous mutations at that site can be non-neutral, because silent nucleic acid sequence change (Ks/µ) can significantly modulate the replication, transcription and translation of genetic information. Whereas the fitness of nonsynonymous mutations at that site includes the fitness effects due to both non-silent nucleic acid change (i.e. protein sequence and protein function change) and “silent” nucleic acid change due their aforementioned processes (i.e. $\text{Ka}/\upmu =\text{Ka}/\text{Ks}*\text{Ks}/\upmu )$. When the “silent” fitness effects of nucleic acid changes at nonsynonymous sites, that excluding the related protein fitness change, is similar to the fitness effects of synonymous sites (Yang and Nielsen 2008), 6 can be expressed as 7:

7 $$\frac{c}{\mu } = Ps*\left( {\frac{Ks}{\mu }} \right) + Pa*\left( {\frac{Ka}{{Ks}}*\frac{Ks}{\mu }} \right)$$

where the Ka/Ks test quantifies fitness associated with protein sequence changes only and is left blind to the fitness effects of silent nucleic acid changes (Ks/μ), especially in the cases of ‘junk’ genomic regions like UTR, pseudogenes, introns, and synonymous sites in the TR region.

Only two out of the three ratios (c/µ, Ks/μ, Ka/μ) are independent variables, and the third ratio (Ka/μ) can be calculated from Eq. (7). Equation (7) can be further simplified to Eq. (8) under the assumption that synonymous substitutions are neutral (i.e. Ks = µ):

8 $$\frac{c}{\mu } = Ps + Pa*\left( {\frac{Ka}{\mu }} \right) = Ps + Pa*\left( {\frac{Ka}{{Ks}}} \right) = \frac{{S + A*\frac{Ka}{{Ks}}}}{S + A}$$

Therefore, Ka/Ks infers the same selection pressure as c/µ does, because both are determined by the selection pressure on the nonsynonymous sites (i.e. Ka/μ) when synonymous sites are neutral. In the words when Ks = µ, c/µ = 1 $\Leftrightarrow$ Ka/Ks = 1; c/µ > 1 $\Leftrightarrow$ Ka/Ks > 1; and c/µ < 1 $\Leftrightarrow$ Ka/Ks < 1 (see **Appendix A** for the proof of the six cases of equivalency between c/μ and Ka/Ks). When Ks $\ne$ μ, then Ka/Ks alone may provide a different c/µ ratio and thus the selection pressure of the NT site. Ka/Ks contains only the relative selection pressure between nonsynonymous and synonymous sites, reflecting the fitness change due to the amino acid change (Suzuki and Gojobori 1999; Yang and Bielawski 2000). Nonetheless, it provides a good measurement of protein fitness change without the strict neutrality requirement of synonymous change (Yang and Nielsen 2008). Put together, if Ks/µ≈1, c/µ and Ka/Ks report the same type of overall fitness change, otherwise c/µ and Ka/Ks could report the different type of the fitness change because Ka/Ks misses the non-neutral fitness change due to synonymous mutations (Ks/µ $\ne 1$).

For a NT site, we can further clarify how the different selection effects acting on a given site in “silent” nucleic acid sequences (Ks/µ) and protein sequences (Ka/Ks) contribute toward the overall selection acting on that same site (c/µ). We demonstrate this by substituting Eq. 3 into Eq. 7 to produce Eq. 9**:**

9 $$\frac{c}{\mu } = \frac{Ks}{\mu }\left( {1 + Pa\left( {\frac{Ka}{{Ks}} - 1} \right)} \right)$$

where c/µ can be under effective neutral selection (c = µ) or non-neutral selection (c ≠ µ), and it is directly influenced by Ks/µ and Ka/Ks, the latter is modified by the proportion of nonsynonymous sites (Pa). We define three such cases which lead to c/µ under neutral and non-neutral selection.

In Case 1, if Ka and Ks are under true neutral selection (Ka = Ks = µ, Fig. 2), then c/µ = 1 is also under true neutral selection because the nucleic acid and protein sequences are both under strict neutral selection.

In Case 2, if Ks ≠ µ and Ka = µ or Ks = µ and Ka ≠ µ, then c/µ ≠ 1. In the former condition, both the nucleic acid sequence (Ks/µ ≠ 1) and the protein sequence (Ka/Ks ≠ 1) are under non-neutral selection. In the latter condition, only the protein sequence (Ka/Ks ≠ 1) is under non-neutral selection. Nonetheless, under both conditions, overall (c/µ ≠ 1) is under non-neutral selection.

In Cases 3 and 4, if Ks ≠ µ and Ka ≠ µ, the resulting c/µ will depend on whether the selection forces acting on Ka and Ks balance each other out or not, which is more easily demonstrated using Eq. 6. In Case 3, the weaker and stronger selection forces acting on Ka and Ks balance each other out, producing c/µ = 1 under effective neutral selection. Otherwise, in Case 4, the opposing selection forces of Ka and Ks do not balance each other out, producing c/µ ≠ 1 under non-neutral selection. As an example, using Eq. 6 and assuming Pa and Ps are of equal weight (0.50), Ks/µ = 1.50 and Ka/µ = 0.50, the resulting equation would be (c/µ = (0.50)(1.50) + (0.50)(0.50) = 0.75 + 0.25 = 1). Using more likely weights for Pa (0.75) and Ps (0.25), the Ks/µ and Ka/µ values would have to be modified to produce the same answer (i.e. c/µ = (0.75)(1.25) + (0.25)(0.24) = 0.94 + 0.06 = 1).

When the physical definition of synonymous and nonsynonymous sites is used, a site in TR is either synonymous or nonsynonymous, suggesting either (Ps = 1, Pa = 0) or (Ps = 0, Pa = 1). Thus, Eq. 7 will be simplified into two simple equations, one is for a synonymous site (10) and the other is for a nonsynonymous site (11):

10 $${\raise0.7ex\hbox{$c$} \!\mathord{\left/ {\vphantom {c \mu }}\right.\kern-0pt} \!\lower0.7ex\hbox{$\mu $}} = \, {\raise0.7ex\hbox{${Ks}$} \!\mathord{\left/ {\vphantom {{Ks} \mu }}\right.\kern-0pt} \!\lower0.7ex\hbox{$\mu $}} = 1$$

11 $${\raise0.7ex\hbox{$c$} \!\mathord{\left/ {\vphantom {c \mu }}\right.\kern-0pt} \!\lower0.7ex\hbox{$\mu $}} = \, {\raise0.7ex\hbox{${Ka}$} \!\mathord{\left/ {\vphantom {{Ka} \mu }}\right.\kern-0pt} \!\lower0.7ex\hbox{$\mu $}} = {\raise0.7ex\hbox{${Ka}$} \!\mathord{\left/ {\vphantom {{Ka} {Ks}}}\right.\kern-0pt} \!\lower0.7ex\hbox{${Ks}$}}$$

Equation (6) can be simplified to Eq. (12) when the UTR region is considered as “synonymous sites” (i.e., Ps = 1 and Pa = 0):

12 $${\raise0.7ex\hbox{$c$} \!\mathord{\left/ {\vphantom {c \mu }}\right.\kern-0pt} \!\lower0.7ex\hbox{$\mu $}} = \, {\raise0.7ex\hbox{${Ks}$} \!\mathord{\left/ {\vphantom {{Ks} \mu }}\right.\kern-0pt} \!\lower0.7ex\hbox{$\mu $}}$$

because they do not encode protein and will not change protein sequence.

Equation 7 provides the highest resolution of fitness information in a genome as it can report fitness change effects for a specific NT site. But this resolution can be reduced to also provide fitness information of per codon site and per segment. In UTR, estimating fitness per NT sites makes more sense since UTRs don’t encode proteins. Whereas in TR, estimation of fitness effects per codon might make more sense where each codon is reported as an average of 3 NT sites in that codon and is stated per NT site. It has been pointed out that no method reports both Ka and Ks for a specific NT site of a genome (Bierne and Eyre-Walker 2003).

### Approximation of the Mutation Rate (μ) from a Genomic Sequence Dataset

In principle, the mutation rate (µ) and the substitution rate (c_i_) can be obtained from the counting of mutations at each NT site from the samples of the replicated population and the selected population, respectively (Figure S1). However, it is challenging to get the replicated population to obtain the former. We outline an in vitro experimental protocol which does not allow the replicated virus population to undergo selection, which might be able to provide a good estimate of the mutation rate (Wu et al. 2023). Therefore, as an alternative, under near effectively neutral selection for a segment *i* (i.e. c_i_/µ ≈ 1), the mutation rate (µ) can be estimated from the highest segment substitution rate (c_i_ or Ka_i_ or Ks_i_) exhibiting molecular clock out of all measured segments. Hence, after the substitution rate (c) of TR and UTR segments, the synonymous substitution rate (Ks) and nonsynonymous substitution rate (Ka) of all TR segments are calculated, the highest substitution rate of the segment satisfying constant rate is set to be µ, if 100% of its sites are under effective neutral selection. If not, this approximated μ is at least a lower boundary for the true μ value. The upper boundary of µ can be further refined by using the lowest c/µ value of all the experiment-determined adaptive mutations from the literature. Finally, this μ can be further validated by the values from in vitro cell-based and cell-free experiments if available.

### Computational Workflow to Generate the Segment Timelines and Relative Substitution Rate Values

**Fig. 3:**
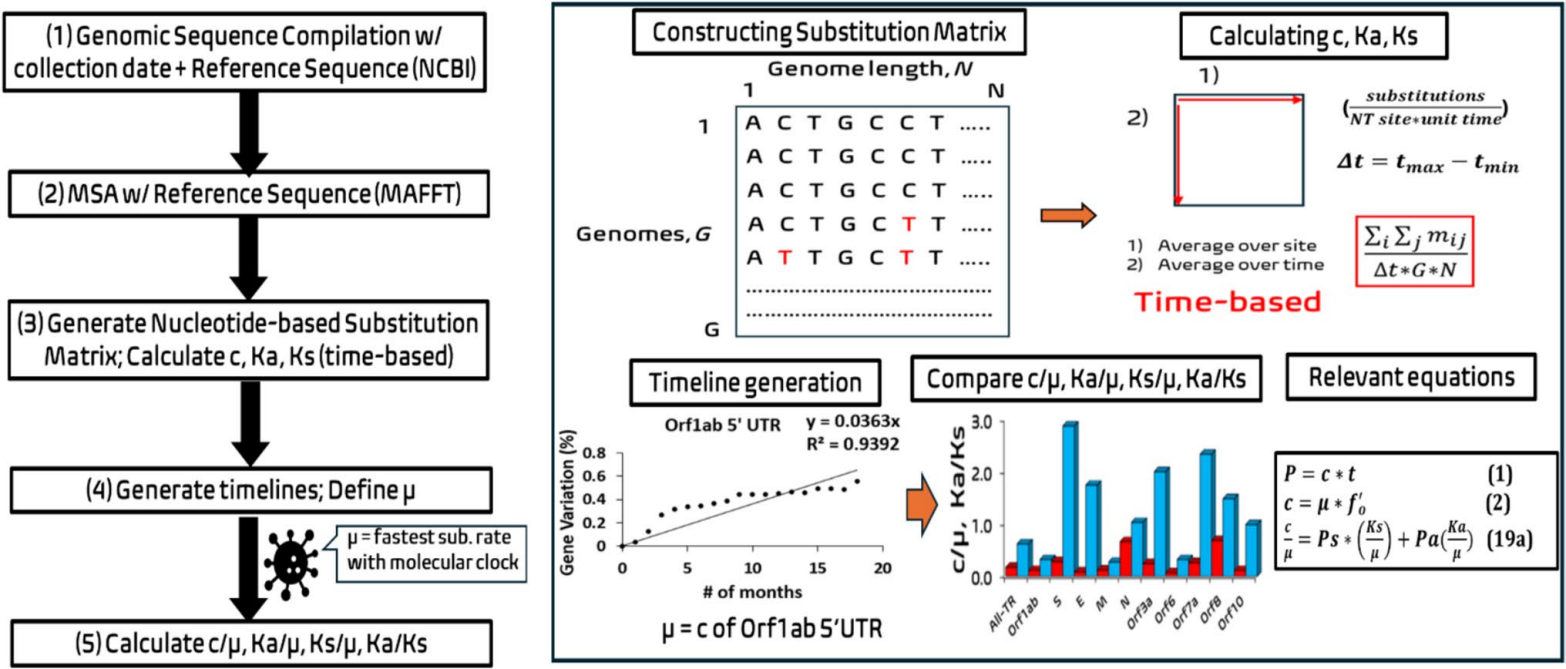
Computational workflow to determine the relative substitution rates for each genetic segment within the studied SARS-COV-2 genomic datasets. (1) Compile the three SARS-COV-2 genomic datasets (11,198 sequences) containing 29,903 NTs. The sequences were collected between December 2019 and June 2021 and compiled into three datasets (A1a, A1b, A1c) and the NCBI reference sequence Wuhan-Hu-1 (Accession number: NC_045512.2) was also compiled. (2) Multiple sequence alignment (MSA) of the NCBI reference sequence against the three genomic datasets was performed using the standard MAFFT protocol. (3) Generate the NT-base substitution matrix for each genomic dataset to calculate the total substitution rate (c), nonsynonymous substitution rate (Ka) and synonymous substitution rate (Ks). Ka and Ks were calculated using the Nei-Gojobori (NG) method. (4) Generate the substitution timelines (c, Ka and Ks) for each segment. Obtain the Coefficient of Determination (R^2^) values for each segment (c R^2^, Ka R^2^ and Ks R^2^). Designate the highest c, Ka or Ks with molecular clock feature as the approximated value for the fundamental genomic mutation rate (μ). (5) Calculate the relative substitution rate values c/μ, Ka/μ and Ks/μ and Ka/Ks for each segment

We present a comprehensive computational workflow to determine time-based segment substitution rates, timelines, definition of the fundamental genomic mutation rate (μ) and relative substitution rates (c/μ, Ka/μ and Ks/μ) for the three SARS-COV-2 genomic datasets in this study (Fig. 3).

### Proving c/µ Could Offer More Information than Ka/Ks for Detecting Molecular Adaptation in Principle (Table 2)

**Table 2:** Comparison between Ka/Ks and c/µ

Ka/Ks (per gene)	c/µ (per NT site)
Neutrality assumption of synonymous sites is required to quantify overall fitness	Neutrality assumption of synonymous sites is not required to quantify overall fitness
This method quantifies fitness at protein level only (Ka/Ks) Cannot explain fitness change due to silent nucleic acid change (Ka/µ), especially in the case of UTR, pseudogene, intron, and synonymous sites	This method quantifies the overall fitness change due to both non-silent (Ka/µ) and silent (Ks/µ) nucleic acid mutations. Can also explain fitness at UTR, pseudogene, intron, and synonymous sites $\frac{c}{\mu } = {\text{Pa}}\left( {\frac{{{\text{Ka}}}}{{{\text{Ks}}}} \times \frac{{{\text{Ks}}}}{\mu }} \right) + {\text{Ps}}\left( {\frac{{{\text{Ks}}}}{\mu }} \right)$ (**7**)
The Ka/Ks ratio depends on the selection of Markovian mutation models and a codon table. A suitable model and a codon table needs to be selected to calculate Pa, Ps, Ka, Ks	The c/µ ratio does NOT depend on the selection of Markovian mutation models and a codon table, as long as the overall mutation rate is time-independent and site-independent$\mu = \sum\nolimits_{i} {\pi_{i} } \sum\nolimits_{j \ne i} {\mu_{ij} }$ $\pi_{i}$ = equilibrium frequency of NT *i*; $\mu_{ij}$ = mutation rate from NT *i*—*j*
Ks is not a good neutral reference for determining selection type, as it is not truly neutral	This method provides a better neutral reference state (µ), because the mutation step can be done in vitro without going through the selection in principle
This method provides a good estimate of fitness at gene level At codon level, the Ka/Ks ratio is very likely undefined as Ks is likely approaching to zero (Ks ≈ 0), because of strong negative selection on the synonymous site or fewer sequence samples. As a result, although the methods for Ka/Ks per codon are available, the values are rarely reported in the literature Harder to determine true adaptive mutations	This method can accurately quantify fitness even at NT site level with no such error Easier to determine true adaptive mutations
Because of low resolution Ka/Ks per gene, timelines, Site Substitution-Mutation Rate Ratio Spectra (SSMRRS), Distribution of Fitness Effects (DFE), and fraction of selection types (${f}^{-}, {f}_{0}^{-},{f}_{0} , {f}_{0}^{+} and {f}^{+}$) CANNOT be determined, and existing evolution theories cannot be evaluated One of the reasons that the long-standing evolutionary debate remains unresolved	Using high resolution c/µ per NT, timelines, SSMRRS, DFE, and the fractions can be easily determined The fractions can be used to evaluate the evolution theories This method provides a solid foundation that can lead to resolution of the debate

The detailed comparison between Ka/Ks and c/µ are listed in Table 2**,** and the following is a brief discussion. The general Eq. (7) described above shows that Ka/Ks and c/µ will report similar fitness change effects only when synonymous sites are truly neutral (Ks = µ). However, there is growing evidence that suggests otherwise (Chamary et al. 2006) (Shen et al. 2024, 2022) (Zhang et al. 2023). This means that Ka/Ks may underestimate the fitness change due to synonymous mutations and contain information on protein sequence change only, whereas c/µ provides overall fitness change information (based on both protein and NT sequence change) even in the low complexity genomic regions like UTRs and non-coding genes.

To calculate Ka and Ks values to estimate the rates and then their ratio (Ka/Ks), a Markovian mutation model and a genetic codon table must be selected, results of which, as pointed out earlier, would be different based on the model chosen for calculating the rates. Because the mutation model and genetic codon table are likely the consequence of selection, these parameters could not be predetermined ahead of selection. The prior assumption on these parameters can lead to biased assessment of the selection pressure using Ka/Ks (Bierne and Eyre-Walker 2003). In contrast, c/µ does not require the mutation model and the codon table in theory and simply counts the substitutions to estimate the rate. This holds true as long as the mutation rate is time independent for the selection period, and this makes the mutation rate (µ) a better reference state, since it is a non-zero number which is truly neutral, unlike Ks, because synonymous sites are not truly neutral. Nonetheless, an unbiased determination of µ remains to be addressed, given the experimental challenges to generate the sequence ensemble without going through selection and to detect mutations due to very low mutation rate. Having said that, we acknowledge that Ka/Ks is a good estimate of amino acid fitness at gene level because Ks is averaged over sites and could be a non-zero number, despite not being truly neutral. But at codon level, Ka/Ks ratio can be undefined when Ks approaches 0 due to purifying selection on synonymous sites. This issue can lead to false infinity predictions and may inaccurately report fitness effects at codon level. This problem was reported (Spielman and Wilke 2015) and Ka/Ks values per codon have hardly been reported, making it harder to identify adaptive mutations. Due to this low-resolution reports of Ka/Ks, the timelines, Site Substitution-Mutation Rate Ratio Spectrum (SSMRRS), Distribution of Fitness Effects (DFE) as well as the fraction of selection types (${f}^{-}, {f}_{0}^{-},{f}_{0} , {f}_{0}^{+} and {f}^{+}$) cannot be estimated using Ka/Ks. This also makes it difficult to evaluate evolution theories using Ka/Ks and could possibly be the reason that the evolutionary debate is still ongoing (de Jong et al. 2024). Whereas c/µ can accurately determine fitness even at NT level with no such problems and hence make it easier to identify truly adaptive mutations, and this high resolution provides better timelines, SSMRRS, DFE and fractions, that makes it easier to evaluate evolution theories and resolve the debate.

### Position-Based and Time-Based Approaches for Calculating Substitution Rates (c, Ka or Ks)

Calculating c, Ka and Ks involve counting the number of total, nonsynonymous or synonymous substitutions at each NT site over a given time interval using multiple sequence alignment with MAFFT (Katoh et al. 2019). The substitution rate, defined as the number of substitutions per NT site per unit time, is calculated from a sequence set of size *G* (containing a number of *g*enomic sequences) that is aligned against a reference sequence of length *N* (containing a number of NTs). From this aligned sequence set, a two-dimensional substitution count matrix (m_ij*,*_ where *i* and *j* are the Time and Position dimensions which comprise the matrix, respectively) is generated using the various collection dates and genome positions. From Eq. (9), the average substitution frequency across position and time can be calculated using either time-based or position-based approaches:

13 $$\frac{{\mathop \sum \nolimits_{i} \mathop \sum \nolimits_{j} m_{ij} }}{\Delta t*G*N} = \frac{{\mathop \sum \nolimits_{j} \mathop \sum \nolimits_{i} m_{ij} }}{N*\Delta t*G} \left( {\frac{substitutions}{{NT site * unit time}}} \right)$$

In the time-based approach of Eq. (12, left), substitution counts are averaged across positions to identify temporal trends, then averaged over time ($\Delta t={t}_{max}-{t}_{min})$. In the position-based approach in Eq. (12, right), substitution counts are averaged across time to obtain the site substitution-mutation rate ratio spectrum (SSMRRS), then averaged across positions.

### Statistical Determination on Constancy of Substitution Rate

Both time-based and position-based methods provide distinct insights into substitution dynamics, highlighting temporal and spatial trends, respectively. One key difference with the time-based approach is that it allows least squares fitting (y = ax, R^2^) to the generated timeline in calculating the absolute rate, which might be different from the position-based rates due to the assumption of a constant rate in the time-based approach. The coefficient of determination (R^2^) from the least square fit offers a statistical measure on how good the time-independent constant rate holds, which is one of two critical hallmarks of neutral substitutions: time-independent rate and equal rate at any site in a genome. The constant rate could help improve the accuracy of (c) by averaging out the noise associated with random sampling. Therefore, the absolute time-based rate value will differ from the position-based rate by a scaling constant ($\frac{\sum_{i}{t}_{i}}{G*\Delta t}$), which is removed upon calculating the relative rates (c/µ, Ka/µ, and Ks/µ). In this way, both methods can yield similar relative rate values if the molecular clock holds. If R^2^ is relatively high (R^2^ > 0.6000) indicating a decent rate constancy, then the time-based relative rates can be used to infer selection pressure. Otherwise, the relative rates from the position-based approach should be used. It should be noted that R^2^ is impacted by (1) the presence of signal(s) (i.e., substitution(s)), (2) the number of signal datapoints which are plotted and (3) the time-dependency of the signal between each datapoint within the time period studied. Because R^2^ is calculated for the timeline of each segment where the size of the segment is constant, R^2^ does not depend on the size of the segment but the time-dependence of substitution number. We also note that R^2^ forces the trendline to go through the origin of the substitution timeline (0,0) reflecting the change in percent NT substitutions at time zero. The trendline can also be fitted using the correlation coefficient (r^2^) such that it does not pass through the origin but attempts to better fit the substitution data and to avoid overfitting. Using r^2^ may be more appropriate for cases where the sample size is small or numerous lineages are within the substitution data. We checked to see if R^2^ and r^2^ for the SARS-COV-2 genomic substitution rate timeline were significantly different; no significant difference in R^2^ (0.9957) or r^2^ (0.9714) was evident (**Figure S7**), therefore we chose to use R^2^ for the remainder of our strict molecular clock analysis.

### Ka, Ks and Ka/Ks Calculations for Coding Gene Segments

**Fig. 4:**
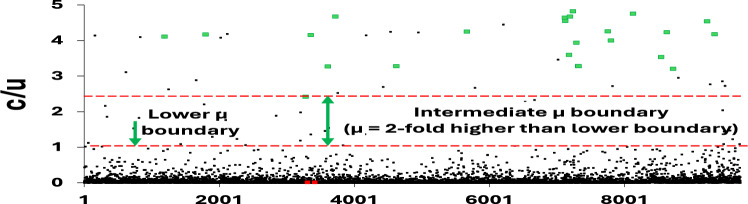
Lowering upper boundary for the mutation rate μ of SARS-COV-2 using literature-validated 51 adaptive mutations (Green dots). Two lethal mutations reported in literature are also consistent with our predictions in a red box (Color figure online)

The nonsynonymous substitutions (Ka), synonymous substitutions (Ks) and Ka/Ks ratio values were calculated across each coding gene for the three datasets using the Nei-Gojobori (NG) (Nei and Gojobori 1986) method, which is based on JC69. NG was chosen based on its consistency with Li-Wu-Luo (LWL) (Li et al. 1985) based on K2P, Pamilo-Bianchi-Li (PBL) (Pamilo and Bianchi 1993) and maximum likelihood (ML) (Goldman and Yang 1994) methods based on HKY85 in processing these SARS-COV-2 datasets demonstrated in our previous paper (Wu et al. 2023). The very similar Ka, Ks and Ka/Ks values from these methods suggest that the bias in transition and transversion rates and the bias in codon usage are not significant in these datasets (Fig. 4).

## Results

### Determination of Mutation Rate (µ) of SARS-COV-2 Using Sequence Data

**Fig. 5:**
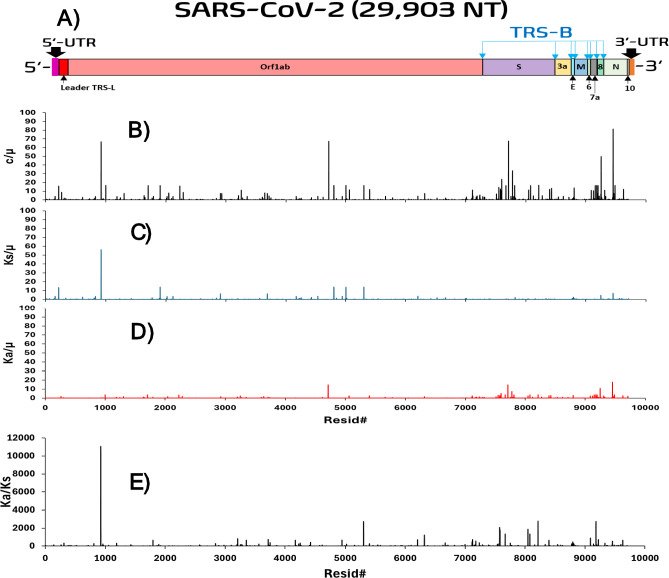
The relative ratio site substitution-mutation rate ratio spectra (SSMRRS) of the SARS-COV-2 All-TR. **A** The structure of the SARS-COV-2 genome. **B** Position-based c/µ at each codon in All-TR over 19 months. **C** Position-based Ka/µ for each codon site in All-TR over 19 months. **D** Position-based Ks/µ for each codon site in All-TR over 19 months. **E** Position-based Ka/Ks for each codon site in All-TR over 19 months, excluding sites with only nonsynonymous substitutions that their Ka/Ks are not defined

In our previous study (Wu et al. 2023), µ was arbitrarily defined as the genomic substitution rate (GSR) derived from Near-Neutral Balanced Selectionist Theory/NNBST$c = \mu f^{\prime}_{0}$, where $f^{\prime}_{0}$ is the fraction of sites in the genome under near-neutral selection. The GSR was calculated from least square fitting of genomic substitutions per month over 19 months exhibiting a constant rate feature (R^2^ = 0.9833) using ($\text{P}=\text{c}*\text{t}$). By setting the expression ($\upmu =\text{c}$), the fraction of mutation sites under near-neutral selection in the genome is set to one ($f^{\prime}_{0} = 1$). However, some segments (in order of decreasing substitution rate: Orf1ab 5’UTR, Orf8, N, all-UTR, S and Orf3a) exhibited a good molecular clock feature (R^2^ > 0.6000) and a much higher substitution rate compared to GSR (Orf1ab 5’UTR: 5.46-fold faster than GSR). If GSR is still defined as µ, $f^{\prime}_{0} \;\rangle \;1$ would occur for all sites in Orf1ab 5’UTR. To circumvent this violation, the fastest segment substitution rate exhibiting a molecular clock feature, (c) of Orf1ab 5’UTR, was defined as µ. We denote that this approximated μ is most likely a lower boundary for the true μ value for SARS-COV-2 (if and when it is experimentally verified) and is sufficient for at least escaping the $f^{\prime}_{0} \;\rangle \;1$ violation for this dataset. The upper boundary of µ can be further refined as described in the methods, but must be done carefully; if one uses the fastest NT substitution rate as the upper boundary of μ (e.g., C241T of Orf1ab 5’UTR:$\frac{\text{c}}{\upmu }=178.1$) (Paradis and Wu 2024), all other genomic sites will be under deleterious selection and hence no adaptive sites would be observed. From the observed top mutation effects that benefit virus fitness, using this upper boundary of μ value would be nonsensical and inconsistent with literature. Thus, we can be confident that the true upper boundaries of μ must be lower than the fastest NT rate. Following the same logic, when more single point adaptive mutations can be determined via biological studies, the upper boundary of μ can be lowered by picking the lowest c/µ value of experiment-determined adaptive mutations (e.g., G15S of NSP5: $\frac{c}{\mu }=2.42$ in Fig. 5). (Paradis and Wu 2024) This updated lower boundary of µ (twofold higher than the lower boundary) provides a more reasonable range for containing the true µ, at least for SARS-COV-2 (Table 3).

**Table 3:** Comparison of the approximated SARS-COV-2 mutation rate in this study to the experimentally determined cell line substitution rates and polymerase fidelity rates in the literature

Study type	Virus strain	Method	Cell line	Key cell receptors	MOI	#Cell passages	Total cell passage time (hours)	#Muts after cell passaging	S/N/Y (E-03)	Major figure#	R (hours)	S/N/R (E-06)	Ref
In vivo (population)	Wuhan-Hu-1	ENSR	N/A	hACE2, hTMPRSS2	N/A	N/A	N/A	N/A	4.30	N/A	N/A	N/A	This study
In vitro (cells)	BT20.1	Plaque assay	WD-PNECs	hACE2, hTMPRSS2	0.1	4	72	4	16.27	Figure 4C	5	9.29	Bamford et al
In vitro (cells)	USA-WA1/2020	RT- qPCR	Caco2	hACE2, hTMPRSS2	0.1	5	72	3	2.44	Figure 3	2	0.56	Chen et al
In vitro (cells)	B.1.1.7	RT- qPCR	Caco2	hACE2, hTMPRSS2	0.1	5	72	8	6.51	Figure 2	8	5.95	Feng et al
In vitro (cells)	hCoV-19/Singapore/2/2020	RT- qPCR	NECs	hACE2, hTMRPSS2	0.1	3	72	2	2.71	Figure 2	6	1.86	Gamage et al
In vitro (cells)	B.1.1.7	Plaque assay	A549	hACE2	0.1	4	24	2	6.10	Figure 1A-1E	6	4.05	Meganck et al
In vitro (cells)	B.1.1.7	Plaque assay	AT2	hACE2, hTMRPSS2	0.1	4	24	2	6.10	Figure 1A-1E	10	7.21	Meganck et al
In vitro (cells)	B.1.1.7	Plaque assay	LAE	hACE2, hTMRPSS2	0.1	4	24	2	6.10	Figure 1A-1E	4	2.48	Meganck et al
In vitro (cells)	B.1.1.7	Plaque assay	NEC	hACE2, hTMRPSS2	0.1	4	24	2	6.10	Figure 1A-1E	6	3.96	Meganck et al
In vitro (cells)	B.1.1.7	Plaque assay	SAE	hACE2, hTMRPSS2	0.1	4	24	2	6.10	Figure 1A-1E	4	2.52	Meganck et al
In vitro (cells)	BA.1	RT- qPCR	Calu3	hACE2, hTMRPSS2	0.01	3	72	15	20.34	Figure 3A	5	13.93	Savellini et al
In vitro (cells)	UVE/SARS-CoV-2/2021/FR/7b	RT- qPCR	Caco2	hACE2, hTMRPSS2	0.001	2	96	2	3.05	Figure 1B	11	3.83	Touret et al
In vitro (cells)	Portugal/PT0054/2020, Portugal/PT1136/2020	RT- qPCR	Vero E6	hACE2	0.1	15	24	18	1.35	N/A	N/A	N/A	Amicone et al
In vitro (cells)	SP02/BRA	RT- qPCR	Vero E6	N/A	0.02	7	48	2	1.74	Figure 2B	5	1.00	Araujo et al
In vitro (cells)	BT20.1	Plaque assay	Vero E6	N/A	0.01	4	72	4	16.27	Figure 4A	8	14.86	Bamford et al
In vitro (cells)	BT20.1	Plaque assay	Vero E6	hACE2, hTMPRSS2	0.01	4	72	4	16.27	Figure 4B	4	7.43	Bamford et al
In vitro (cells)	USA-WA1/2020	RT- qPCR	Vero E6	hACE2, hTMPRSS2	0.1	5	72	5	4.07	Figure 3	2	0.93	Chen et al
In vitro (cells)	B.1.1.7	RT- qPCR	Vero E6	N/A	0.1	5	72	8	6.51	Figure 2	6	4.46	Feng et al
In vitro (cells)	Australia/VIC01/2020	Plaque assay	Vero E6	hACE2, hTMRPSS2	3	4	48	5	7.63	Figure 2	3	2.61	Ogando et al
In vitro (cells)	SZH-UZH-IMV1/2020	Plaque assay	Vero E6	N/A	0.01	3	96	10	10.17	Figure 1	4	4.64	Pohl et al
In vitro (cells)	UVE/SARS-CoV-2/2021/FR/7b	RT- qPCR	Vero E6	hTMPRSS2	0.001	2	96	2	3.05	Figure 1A	6	2.09	Touret et al
In vitro (cells)	SARS-COV-ExoN1	Plaque assay	Vero E6	N/A	0.1	20	24	23	1.83	Figure 3	12	12.00	Eckerle et al
In vitro (cells)	SARS-COV-WT	Plaque assay	Vero E6	N/A	0.1	20	24	3	14.04	Figure 3	12	0.90	Eckerle et al
Enzyme kinetics	Synthetic	Polymerase fidelity	N/A	N/A	N/A	N/A	N/A	N/A	N/A	N/A	N/A	8400.00	Yin et al

^*^Converting factors: Length of SARS-COV-2 genome (NTs: 29,903), Length of SARS-COV-1 genome (NTs: 29,727). The slope of the linear portion of growth curve data was used to calculate the time to complete one infection cycle (R)

^**^Human Cell lines: **A549**: Adenocarcinomic human alveolar basal epithelial cells; **AT2**: Alveolar Type 2 organoid; **Caco2**: Colorectal adenocarcinoma cells; **Calu3**: Lung adenocarcinoma cells; **NEC**: Nasal epithelial cells; **LAE**: Large airway epithelial cells; **SAE**: Small airway epithelial cells; **WD-PNECs**; Well-differentiated nasal epithelial cells. **Simian cell lines**: **Vero E6**: African green monkey kidney cells. **Key cell receptors**: **hACE2**: Human angiotensin converting enzyme type 2; **hTMRPSS2**: Human transmembrane protease serine 2

**Terms to define. MOI**: Multiplicity of infection; **#Cell passages:** The number of cell passages which took place during the study; **#Muts after cell passaging**: Mutations accumulated in the virus genome was determined using genomic sequencing of the virus after the last cell passaging event with the virus strain reference sequence; **Major figure/table #**: Figure and/or table numbers used to extract growth curve and cumulative mutation information from the cell line study. **R:** Time to complete one virus infection cycle during cell passaging (hours); **S/N/Y**: substitution rate (substitutions per site per year) scaled by 1 × 10^3; **S/N/R**: substitution rate (substitutions per site per infection cycle) scaled by 1 × 10^6. **Ref:** Citation of the study

**Equations. S/N/**Y= ((#Mutations after cell passaging) * (365 days) / (Total cell passaging time/24 h)*(genomic sequence length)); **S/N/R** = ((# Mutations after cell passaging) / (Total cell passaging time / R (hours) * (genomic sequence length));${{\varvec{T}}}_{{\varvec{g}}{\varvec{e}}{\varvec{n}}}=\frac{{{\varvec{l}}{\varvec{o}}{\varvec{g}}}_{10}^{2}}{{{\varvec{k}}}_{{\varvec{l}}{\varvec{o}}{\varvec{g}}}}$***,**** T*_*gen*_: the generation time or doubling time; *K*_*log*_: the specific growth rate in log_10_ units;${log}_{10}^{2}$:the logarithm (base 10) of 2, a scaling factor

### Our Inferred Mutation Rate (µ) from In vivo SARS-COV-2 Sequence Data is Consistent with the Experimentally Determined In vitro Cell-based and Cell-free Substitution Rates from the Literature

To validate the accuracy of our approximated µ value from in vivo human SARS-COV-2 sequence data (substitutions per NT site per year, S/N/Y), we compared it to the substitution rate values calculated from in vitro cell-based and cell-free experiments (Table 4) (Amicone et al. 2022; Araujo et al. 2020; Bamford et al. 2022; Chen et al. 2023; Feng et al. 2022; Gamage et al. 2020; Meganck et al. 2024; Ogando et al. 2020; Pohl et al. 2021; Savellini et al. 2023; Touret et al. 2021) including 9 human cell lines (i.e. Caco2, Calu3, A549, epithelial cells) and 1 African Green Monkey cell line (Vero E6), which are commonly used to study SARS-COV-2 replication kinetics, infectivity and pathogenicity. Studies were only included if they performed cell passaging of SARS-COV-2 and reported both viral growth curve(s) across time (i.e. Viral titer, TCID50/mL, PFU/mL per post-infection time) and observed cumulative genomic mutations after the final cell passaging event. We include the supplementary data as an **Excel file.** Indeed, our approximated µ value (4.30E-03 S/N/Y) falls within the range of the cell line substitution rates [1.35E-03, 20.34E-02 S/N/Y] (Table 4**,** see equation for calculations), with an average rate of 7.49E-03 S/N/y, suggesting our µ value is a good approximation for the mutation rate, at least for this virus. The observed deviation in cell line substitution rates is likely due to variations in number of cell passages performed, duration of a single cell passage, total passage time, cell line used, key cell surface receptor expressed in the cell line, multiplicity of infection (MOI), virus strain used and experimental protocol.

**Table 4:** Position-based c/µ, R^2^ regression parameter and function from the time-based c/µ analysis for All-UTR, All-TRS and each UTR and TRS of SARS-COV-2 over 19 months

Seg (NT length)		c/µ	c R^2^	Gene Function
**All**	**UTR (771)**	**0.52 ± 0.02(H-)**	**0.7167**	n/a
	**TRS (61)**	**0.07 ± 0.00(H-)**	**0.6299**	n/a
**Orf1ab**	**5’UTR (265)**	**0.97 ± 0.02**	**0.9392**	Directs viral mRNA to ribosome
	**TRS-L (7)**	**0.01 ± 0.02(L-)**	0.2699	TRS-L
**S**	**5'UTR (7)**	**0.01 ± 0.01(L-)**	0.0239	N/A
	**TRS-B (7)**	**0.01 ± 0.01(L-)**	− 0.0075	TRS-B
**E**	**5'UTR (24)**	**0.05 ± 0.00(L-)**	0.0399	N/A
	**TRS-B (6)**	**0.00 ± 0.00(L-)**	N/A	TRS-B
**M**	**5'UTR (50)**	**0.06 ± 0.01(L-)**	0.4925	N/A
	**TRS-B (7)**	**0.00 ± 0.00(L-)**	N/A	TRS-B
**N**	**5'UTR (14)**	**0.90 ± 0.07(L-)**	0.5577	N/A
	**TRS-B (7)**	**0.03 ± 0.00(L-)**	0.2535	TRS-B
**Orf3a**	**5'UTR (8)**	**0.00 ± 0.00(L-)**	− 0.0235	N/A
	**TRS-B (7)**	**0.01 ± 0.00(L-)**	− 0.0402	TRS-B
**Orf6**	**5'UTR (10)**	**0.03 ± 0.02(L-)**	0.2871	N/A
	**TRS-B (6)**	**0.03 ± 0.02(L-)**	− 0.2110	TRS-B
**Orf7a**	**5'UTR (6)**	**0.34 ± 0.05(L-)**	0.2692	N/A
	**TRS-B (7)**	**0.29 ± 0.04(L-)**	0.3129	TRS-B
**Orf8**	**5'UTR (134)**	**0.23 ± 0.01(L-)**	0.4862	N/A
	**TRS-B (7)**	**0.27 ± 0.08(L-)**	0.4698	TRS-B
**Orf10**	**5'UTR (24)**	**0.27 ± 0.06(L-)**	0.2323	N/A
**Orf10**	**3'UTR (229)**	**0.38 ± 0.04(L-)**	0.1415	Modulates RNA replication

^*^c/µ calculated with reference to Orf1ab 5’UTR substitution rate (µ = 36.1E-03% substitutions/NT site/month; c = total substitutions per NT site per year). Selection type is positive ( +) if c/µ > 1, negative (–) if c/µ < 1 or near-neutral if c/µ ≈ 1

^**^c/µ is classified with letters for high R2 (H, R2 > 0.6000), low R2 (L, R2 < 0.6000), positive c/µ ( +) or negative c/µ (-) values. See Table S1 for the average absolute substitution rate (c) value for All-UTR, All-TRS, each UTR and TRS and each segment calculated from the three datasets

As is, these yearly substitution rates hide the substitution rate at the molecular level (i.e., during a single virus genome replication event), also known as the substitution rate per virus generation (i.e. substitutions per NT site per generation, S/N/R), where T_R_ is the time for a single generation event (i.e. doubling of population). If determined accurately, this substitution rate per generation can provide insight into the polymerase copy error during a single replication event, which is the primary source of the mutation rate under neutral selection. Additionally, this substitution rate per generation more closely reflects Gillespie’s concept of “weak mutation, strong selection”, where new mutations occur at a very low frequency, yet the selection pressure acting on these mutations is very high due to large population size (e.g. ~ 10^3^ virions of burst size), such that small fitness advantages or disadvantages profoundly impact genomic fitness (Gillespie 1983). Using the growth curve data to approximate (T_R_), the number of generations per total cell passage time and the cumulative mutation data were used to calculate the substitution rate per generation across each cell line study (see Table 4 for calculation details). These rates were then compared to the polymerase error rate with its proofreading 5′→3′ exoribonuclease machinery (9.00E-07 per NT site per replication) determined from SARS-COV infection of Vero E6 cells. The SARS-COV-2 substitution rate per generation across each cell line [5.57E-07, 1.49 E-05 S/N/R], with an average rate of 5.07E-06 S/N/R, were generally consistent with the polymerase error rate with its proofreader (9.00E-07 substitutions per NT site per replication cycle) (Eckerle et al. 2010). In contrast, the polymerase error rate is significantly elevated when its proofreader is mutated (1.20E-05 S/N/R) (Eckerle et al. 2010) and removed completely (8.40E-03 S/N/R) (Yin et al. 2023), causing a reduction in viral growth fitness and justifying the importance of maintaining high polymerase replication fidelity.

### c/µ, Ka/µ, Ks/µ and Ka/Ks were Obtained from a Time-Based Approach

**Fig. 6:**
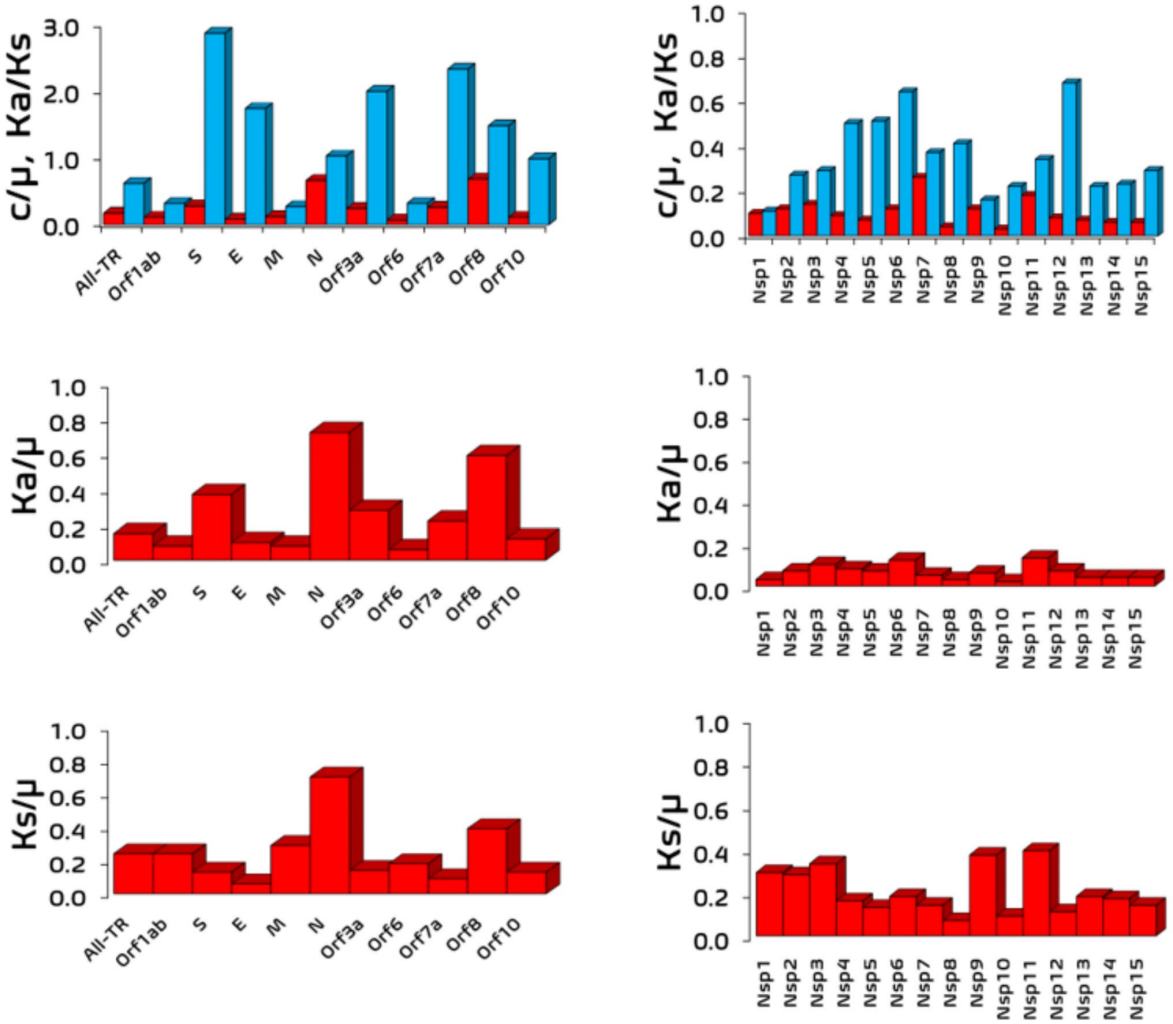
c/μ versus Ka/Ks, Ka/u and Ks/u values of each coding gene (left column) and NSP (right column). In the first row, c/µ and Ka/Ks are represented in red and blue bars, respectively (Color figure online)

To validate c/µ and Ka/Ks values (per NT and per unit time) reported in our previous paper (Wu et al. 2023) obtained via position-based approach in which SSMRRS was used to get the rates (per unit time per NT) and their ratios (Fig. 6), these two ratios plus Ka/µ and Ks/µ are obtained in this study from the time-based approach (Table S1–S12**, Figure S2-S6**) in which a temporal trend (timeline) is fitted by least square (y = m*x) to obtain the rates and thus their ratios from the three independent genome sequence datasets used in our previous paper (Wu et al. 2023). In addition, R^2^ from the least square fit offers a statistical measure on how good the molecular clock feature (i.e. time-independent constant rate) holds, which is one of two critical hallmarks of neutral substitutions: time-independent rate and equal rate at any site in a genome (Fig. 2**)**.

### Molecular Clock was Observed for the Genome, All-UTR, All-TR, 5’UTR of Orf1ab, and Most Genes and NSPs of SARS-COV-2

The raw time-based total substitution rate (c) values were tabulated (UTR/TRS: Table S1 and **Figure S2-S3; and** TR: Table S2 and **Figure S4**) as well as the raw Ka, Ks, Ka R^2^, Ks R^2^ and Ka/Ks values (**Tables S8-S12,** and **Figure S5-S6)**. The high R^2^ values of the genome, All-UTR, All-TR, 5’UTR of Orf1ab, and most genes and NSPs of SARS-COV-2 indicate their molecular clock features (R^2^ > 0.6000). In contrast, the low R^2^ values of All-TRS, most UTR and TRS segments, the remaining genes and NSPs indicate the absence of molecular clock features (R^2^ < 0.6000)**.** Indeed, only 3 out of 22 non-coding segments exhibited molecular clocks (Table 4).

Specifically, Orf1ab 5’UTR (0.9392), All-UTR (0.7167) and All-TRS (0.6299) are the only non-coding segments which exhibited good molecular clocks, whereas the remaining UTRs and TRSs did not exhibit molecular clocks [− 0.2110, 0.5577]. The conserved nature of E TRS-B and M TRS-B (c/μ = 0) prevented the generation of R^2^ values; thus, no constant rate was observed for these segments (Table 4**).** In contrast to UTR/TRS, 20 out of 25 coding segments in TR showed good molecular clocks [0.6116, 0.9957], whereas the remaining five segments did not exhibit molecular clocks [0.0000, 0.5965] (Table 4).

Next, the Ka R^2^ values indicate 23 out of the 25 coding segments exhibited a good molecular clock (Table 5). Ka R^2^ indicate All-TR (0.9838) Orf1ab (0.9929), S (0.9668), E (0.8859), M (0.7196), N (0.9497), Orf3a (0.9147), Orf8 (0.9148), Orf10 (0.8195), NSP1-NSP6 [0.7883, 0.9773], NSP8-NSP15 [0.8714, 0.9648] and NSP7 (0.6427) exhibit good molecular clocks, whereas Orf6 (0.5536) and Orf7a (0.5531) did not exhibit molecular clocks (Table 5). Lastly, Ks R^2^ values indicate 23 out of 25 coding segments exhibited good molecular clocks (Table 5), specifically All-TR (0.9877), Orf1ab (0.9935), S (0.9252), E (0.6311), M (0.7504), N (0.9435), Orf3a (0.8250), Orf7a (0.8782), Orf8 (0.8751) and NSP1-NSP15 [0.8330, 0.9715] exhibit good molecular clocks, whereas Orf6 (0.2133) and Orf10 (0.4193) do not exhibit molecular clocks.

**Table 5:**
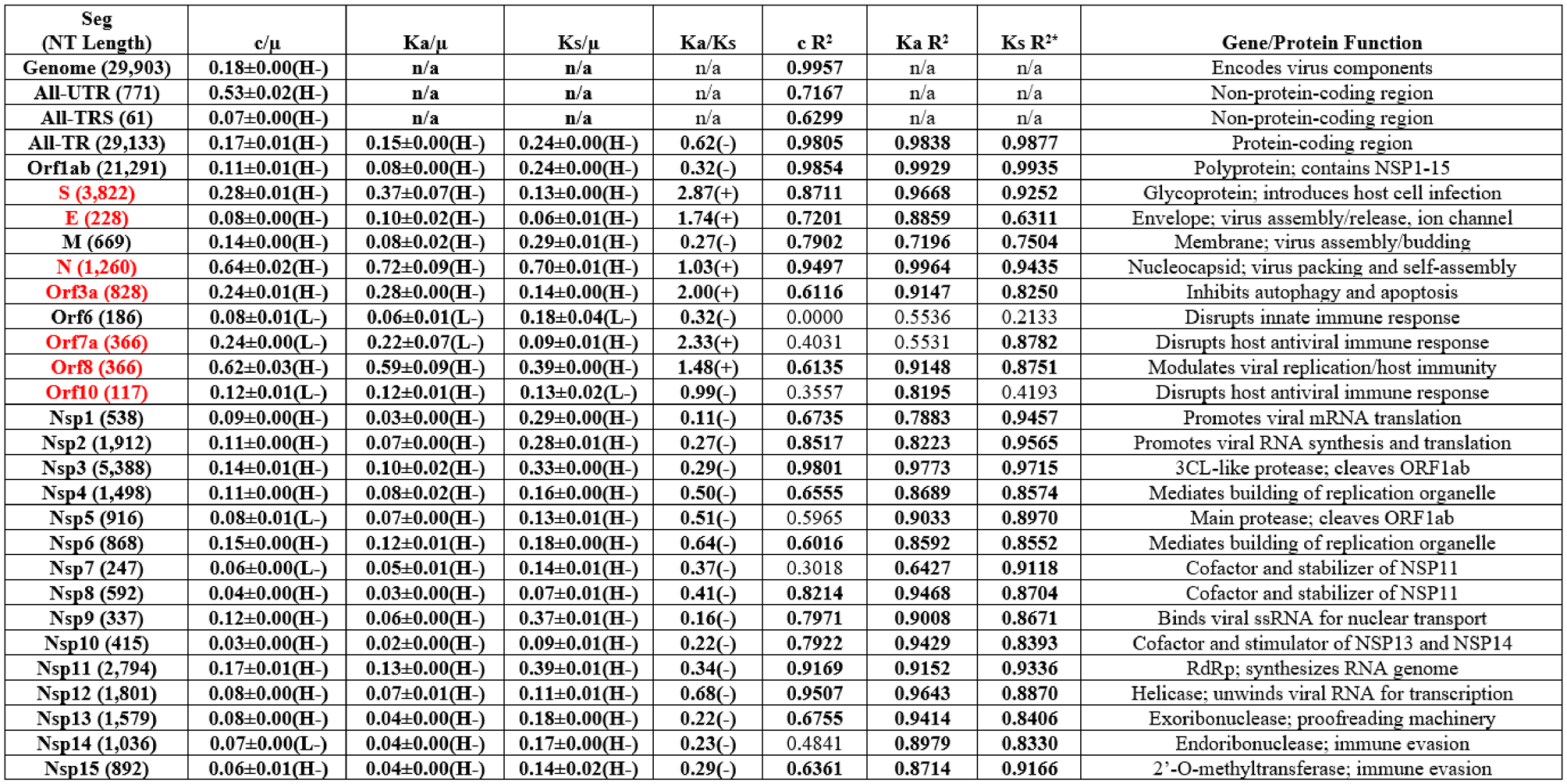
Time-based c/µ, Ka/µ, Ks/µ, Ka/Ks, R^2^ values and Gene/Protein function for the genome, All-UTR, All-TR and each coding segment of SARS-COV-2 over 19 months

*c/µ calculated with reference to Orf1ab 5’UTR substitution rate (µ = 36.1E-03% substitutions/NT site/month; c = total substitutions per NT site per year). Selection type is positive ( +) if c/µ > 1, negative (–) if c/µ < 1 or near-neutral if c/µ ≈ 1. c/µ is classified with letters for high R^2^ (H, R^2^ > 0.6000), low R^2^ (L, R^2^ < 0.6000), positive c/µ ( +) or negative c/µ (-) values. See Table S2 for the average absolute substitution rate (c) value for the genome and each segment calculated from the three datasets

**R**^**2**^: Coefficient of determination; **n/a**: Not applicable

Segments in red highlight the different selection types assigned by Ka/Ks and c/µ

### Time-Based and Position-Based c/μ and Ka/Ks are Highly Consistent for Most of Segments in TR, also Suggesting the Validity of Molecular Clock for these Segments

c/µ and Ka/Ks values calculated using time-based and position-based approaches were assessed for UTR/TRS segments where applicable (**Table S3**) and the TR segments (**Tables S4 and S7)** based on the linearity (R^2^) of c, Ka and Ks obtained from least square fitting, as described in the methods section. Both absolute and percent differences for calculated c/µ and Ka/Ks values between the two methods are provided for the reader’s preference. For the UTR/TRS, the absence of molecular clocks in most segments caused larger deviations in c/µ (**Table S3: [**0.00, 0.71] or [0.00%, 55.56%]) compared to segments with molecular clocks (**Table S3**: [0.03, 0.18] or [1.74%, 8.11%]). For TR, the same observation was made for segments without molecular clocks (**Table S4: [**0.01, 0.54] or [0.53%, 38.57%]) versus segments with molecular clocks (**Table S4:** [0.00, 0.07] or [0.00%, 12.50%]). In contrast, Ka/Ks values calculated from time-based and position-based approaches showed greater deviations compared to c/µ across each segment (**Table S7:** [0.01, 1.07] or [1.51%, 60.00%]). The increased error rate in the time-based Ka/Ks methods indicates reduced robustness compared to c/μ, which may be caused by synonymous sites exhibiting low Ks, which would inflate Ka/Ks values across coding segments.

### c/µ ≠ 1 and Lack of Molecular Clock for the UTR/TRS Indicates They are Not Under Strictly Neutral Selection

The major assumption in the Ka/Ks test that synonymous sites in TR are effectively neutral would also imply that the UTR/TRS is also effectively neutral. This implication will be assessed by checking if the c/µ = 1 equivalency is satisfied for UTR/TRS. Because the molecular clock was absent for each UTR/TRS except All-UTR, All-TRS and Orf1ab 5’UTR, the calculated position-based c/µ values will be assessed here. Only Orf1ab 5’UTR satisfies the c/µ = 1 equivalency due to its substitution rate being defined as µ. Otherwise, the remaining UTRs and TRSs do not satisfy the c/µ = 1 equivalency, indicating they are not under strictly neutral selection (Table 4). Unless otherwise specified, position-based c/μ values Orf1ab 5’UTR (time-based c/μ = 1.00), N 5’UTR (0.90/-), All-UTR (time-based c/μ = 0.53/-), Orf10 3’UTR (0.38/-), Orf10 5’UTR (0.27/-), Orf7a 5’UTR (0.34/-), Orf8 TRS-B (0.27/-), Orf7a TRS-B (0.29/-), Orf8 5’UTR (0.23/-), All-TRS (time-based c/μ = 0.07/-), M 5’UTR (0.06/-), E 5’UTR (0.05/-), Orf6 5’UTR (0.03/-), Orf6 TRS-B (0.03/-), N TRS-B (0.03/-), Orf3a TRS-B (0.01/-), S 5’UTR (0.01/-), S TRS-B (0.01/-), Orf1ab TRS-L (0.01/-), Orf3a 5’UTR (0.00/-), E TRS-B (0.00/-) and M TRS-B (0.00/-) are likely under weak to strong negative selection (Tables 4 and S3).

### Ks/µ ≠ 1 for All Coding Segments Suggest that Synonymous Sites in These Segments are Not Effectively Neutral

The major assumption in the Ka/Ks test that synonymous sites in TR are effectively neutral can be directly assessed by checking if the Ks/µ = 1 equivalency is satisfied for each segment. Ks/µ indicates the fitness change due to the nucleic acid sequence change because synonymous substitutions only impact the nucleic acid sequence composition. The average Ks/µ values and raw Ks/µ values have been tabulated for each coding segment (Tables 5 and S11 and Fig. 6).

None of the 25 coding segments satisfy the Ks/µ = 1 condition [Ks/µ: 0.06, 0.70] Table 5) and are likely under negative selection despite the presence of the molecular clock feature [Ks R2: 0.6311, 0.9935] (Table 5) for 23 out of 25 coding segments. Please note that Ks/µ = 1 with a good molecular clock is not sufficient for indicating strictly neutral selection; a bell-shaped Poisson distribution of Ks/µ centered at Ks/µ = 1 is required (Fig. 2). However, our previous report demonstrated that an L-shaped probability distribution for synonymous substitutions was observed for each coding segment (Wu et al. 2023), indicating Ks/µ for most segments aren’t under strictly neutral selection.

### Varying Ka/µ Values Indicate Fitness Change Due to the Protein and Nucleic Acid Sequence at Nonsynonymous Sites in TR

Ka/µ reflects the fitness change in nonsynonymous coding sites due to protein and nucleic acid sequence change. Unlike Ks/µ which is assumed to be under neutral selection in the conventional Ka/Ks test, we expect Ka/µ to vary between each segment as evidenced by the L-shaped probability distribution for nonsynonymous substitution rates of each coding segment in our previous report (Wu et al. 2023). The averaged Ka/µ and raw Ka/µ values were tabulated (Tables 5 and S10 and Fig. 6**)** to check if Ka/µ varied significantly from neutral selection for all coding segments. Indeed, this was the case for each segment [Ka/µ: 0.02, 0.72] (Table 5) despite the observed molecular clock observed in most segments [Ka R^2^: 0.6427, 0.9929] (Table 5) except Orf6 (0.5536) and Orf7a (0.5531). These findings match our expectation for the nonsynonymous coding sites to be under non-neutral selection, despite their present molecular clock feature.

### Ka/Ks and c/µ Inconsistencies Explain that Ka/Ks Tells the Fitness Due to Amino Acid Sequence Change Only

**Fig. 7:**
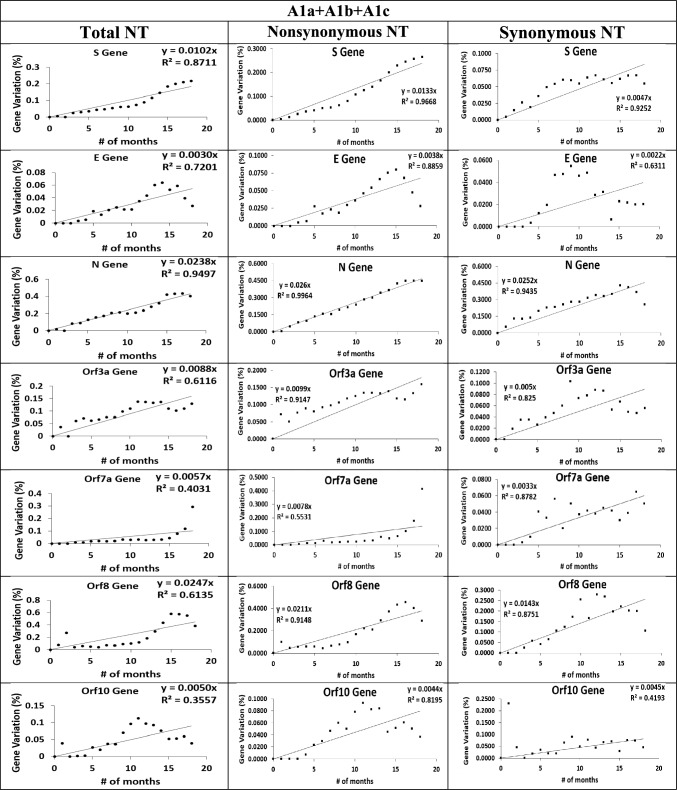
Substitution rates of S, E, N, Orf3a, Orf7a, Orf8 and Orf10 gene segments calculated from linear regression. The timelines for the remaining gene segments are in the supporting document (Figures S5-S6)

Lastly, Ka/Ks measures the relative fitness change at the protein sequence level, but it can provide overall fitness change as c/µ **(**see **Eq. **7**)** if synonymous sites are under neutral selection (i.e., Ks/µ = 1). Otherwise, Ka/Ks might provide a different interpretation due to the fitness change of the nucleic acid sequence change only. Due to none of the segments satisfying the Ks/µ = 1 condition, we expect Ka/Ks might give an incorrect assignment when comparing with the assignment by c/µ. The average Ka/Ks values (Table 5 and Fig. 6) and raw Ka/Ks values for each dataset (**Table S12**) have been tabulated. The time-based Ka/Ks values suggest S (2.87/ +), Orf7a (2.33/ +), Orf3a (2.00/ +), E (1.74/ +), Orf8 (1.48/ +) are under positive selection and N (1.03/ +) and Orf10 (0.99/-) are under almost effective neutral selection (Fig. 7).

However, their c/µ values suggest that these seven segments are under negative selection [c/µ: 0.08, 0.64].

We demonstrate a few example cases where the overall fitness change cannot be exclusively described by Ka/Ks. The Ka/Ks of N indicates almost effective neutral selection at its protein sequence (1.03/ + , Table 5); yet its c/μ indicates overall weak negative selection (0.64/-, Table 5). This nearly neutral Ka/Ks value is due to the very close consistency between its Ka/μ (0.72/-) and Ks/μ (0.70/-). Conversely, NSP1-12 and NSP15 report low Ka/Ks values [0.11, 0.68] (Table 5) indicating negative selection at their protein sequences. The c/µ values are much lower per NSP [0.04, 0.17], indicating strong negative selection; their Ks/μ values are 2.3–3.0-fold higher than their Ka/μ values [Ks/μ: 0.07, 0.39] [Ka/µ: 0.03, 0.13] (Table 5), indicating higher nucleic acid fitness change. Another case is Orf7a, which reports a Ka/Ks indicating strong positive selection (2.33/ +), however its c/µ indicates strong negative selection (0.24/-); this results from a larger Ka/μ (0.22/-) compared to Ks/μ (0.09/-), indicating an increased protein sequence fitness change. A more extreme case is E, with a Ka/Ks indicating weak positive selection (1.74/ +) despite exhibiting stronger negative selection at Ka/μ (0.10/-) and Ks/μ (0.06/-). From here, the interpretation of Ka/Ks alone can give an incomplete and sometimes incorrect determination of a segment’s overall fitness change, based on varying Ks/μ and Ka/μ values.

### The Overall Fitness Change Ascribed by c/µ

Time-based c/µ indicates the overall selection type for N (0.64/-), Orf8 (0.62/-), S (0.28/-), Orf7a (position-based c/μ = 0.24/-), Orf3a (0.24/-), M (0.14/-), Orf10 (position-based c/μ = 0.12/-), Orf1ab (0.11/-), Orf6 (0.08/-), E (0.08/-), All-TR (0.07/-) and NSP1-15 (0.03–0.17/-) are under negative selection (Table 5 & Fig. 6). The NT fitness change from Ks/µ indicates N (0.70/-), Orf8 (0.39/-), M (0.29/-), All-TR (0.24/-), Orf1ab (0.24/-), Orf3a (0.14/-), S (0.13/-), Orf10 (0.13/-), Orf7a (0.09/-), E (0.06/-) and NSP1-15 (0.09–0.39/-) are under negative selection. The nonsynonymous site fitness change by Ka/µ indicates N (0.72/-), Orf8 (0.59/-), S (0.37/-), Orf3a (0.28/-), Orf7a (0.22/-), All-TR (0.15/-), Orf10 (0.12/-), E (0.10/-), M (0.08/-), Orf1ab (0.08/-), Orf6 (0.30/-) and NSP1-15 (0.03–0.13/-) are under negative selection. The overall protein fitness change by Ka/Ks indicates Orf6 (0.32/-), NSP6 (0.64/-), NSP13 (0.22/-) and NSP14 (0.23/-) are under negative selection (Table 5**)**.

## Discussion

A growing body of sequence-based evidence shows that UTR is not just “junk,” and conserved regulatory NT sites in UTR are crucial for life's complexity, making them subject to strong natural selection. As we see in Table 6**,** the increase in UTRs and their conservation (0.2–7.0% of the whole genome) in more complex life forms, from viruses to humans, underscores their critical role in regulating gene expression for more complex life functions (Grainger et al. 2006; Havilio et al. 2005; Lamoureux et al. 2024; Rands et al. 2014; Yocca et al. 2021). By regulating gene expression, non-coding genes in UTR ensure precise control over cellular functions and protein synthesis (Baoxu et al. 2022; Marco and Giorgio 2022). Additionally, non-coding RNAs, such as microRNAs and long non-coding RNAs, are essential for epigenetic regulation, chromatin remodeling, and modulating gene activity (Cen et al. 2022; John et al. 2023; Noa and Igor 2020). It is well known that dysregulation or mutations in non-coding genes are linked to the manifestation of various diseases, including cancer and neurodegenerative disorders (Ekta et al. 2016; Philip and Janice 2023). Conserved UTRs are thus paramount to building and maintaining life complexity through increasing genetic regulation. Despite this, the current Ka/Ks and MutSel frameworks do not address mutations in this region.

**Table 6:** Key genomic properties of major life forms

*Life* *Form*	*Rep* *Species*	*Size*	*N*_*e*_	*Total NT* *sites*	*µ* *(per NT site per year)*	*UTR* *(%)*	*Conserved* *UTR (%)*	*TR* *(%)*	*#* *Protein*
*Viruses*	SARS-COV-2	29.3 Kb	10E6	10E10	4.3 × 10E-3	2.6	~ 0.2	97.4	25
*Bacteria*	E. coli	4.6 Mb	10E8	10E12	5.7 × 10E-6	~ 10.0	~ 6.4	~ 90.0	4,300
*Fungi*	S. cerevisiae	12.1 Mb	10E6	10E13	8.3 × 10E-11 to 3.1 × 10E-8	25.0	~ 7.0	75.0	5,800
*Plants*	A. thaliana	135 Mb	10E6	10E14	2.2 × 10E-8	67.0	~ 7.0	33.0	27,000
*Animals*	Homo sapiens	3.2 Gb	10E5	10E14	8.5 × 10E-11	98–99	~ 3.5	1–2	22,000

Additionally, ~ 25% of the total sequence in the TR consists of synonymous sites, however, increasing evidence points to most synonymous sites are under strong non-neutral selection in representative yeast genes (Shen et al. 2024, 2022) and in mammals (Chamary et al. 2006). Non-neutral selection in synonymous sites can be highly beneficial in drug and vaccine development. For example, reshaping mRNA vaccine topology through synonymous codon optimization significantly enhanced the stability and immunogenicity of SARS-COV-2 mRNA vaccines by > 130-fold in mice (Zhang et al. 2023). Sauna et al. have shown that synonymous mutations are nearly as important as nonsynonymous mutations in causing human disease (Sauna and Kimchi-Sarfaty 2011). From this, Zhang and coworkers proposed reassessing many biological conclusions concerning mutation, selection, effective population size, divergence time, and disease mechanisms that rely on the assumption that synonymous mutations are neutral (Shen et al. 2024, 2022).

To overcome these limitations, we proposed a replication-selection model (Figure S1) that uses the c/µ test to measure selection pressure on any NT site in both the TR and UTR of a viral genome using sequence data (Wu et al. 2023). To demonstrate that c/μ potentially offers a broader scope of application with greater accuracy and sensitivity (Table 2), we derived a general equation (Fig. 1) integrating the overall fitness effect (c/μ) with the fitness effects of protein sequence change (Ka/Ks), nonsynonymous (Ka/μ) and synonymous (Ks/μ) mutations in this study.

As a case study, we conducted a comparative analysis of c/µ and Ka/Ks in detecting molecular adaptation across various genomic segments in SARS-COV-2: 11 UTRs, 9 TRSs, 10 genes encoding 25 proteins. The inequality c/µ ≠ 1 for all UTRs and TRSs suggests that these regions are under selective pressure and not evolving neutrally. This means that either the substitution rate (c) is influenced by natural selection, or the mutation rate (μ) does not solely determine the observed substitution rate. Similarly, the inequality Ks/μ ≠ 1 for all 25 coding genes indicates that the synonymous sites within these coding genes are also not evolving neutrally. Synonymous mutations are generally assumed to be neutral, but this inequality suggests that natural selection might be acting on these sites as well, potentially due to factors like codon usage bias, translational efficiency, or other regulatory mechanisms. Put together, the foundational assumption for the Ka/Ks test to detect the selection pressure on the mutations for SARS-COV-2 is not satisfied, thus Ka/Ks could report an inaccurate fitness impact by overlooking changes due to synonymous mutations (Ks/µ ≠ 1). Indeed, c/µ significantly differs from Ka/Ks for most of the 25 coding genes by including the fitness change due to the nucleic acid sequence change only (i.e. Ks/µ). c/µ and Ka/Ks report the same type of fitness change for 18 out of the 25 coding genes, thus Ka/Ks reports the incorrect type of fitness change for the remaining 7 coding genes.

Ka/Ks also has difficulty in determining true adaptive mutations within a codon when Ks approaches zero (Ks≈0) despite Ka being above zero, producing undefined Ka/Ks values. These false positive sites stem from the core issue being the choice of a variable reference state that can be zero (Ks). To reduce the false positive mutation count due to Ka/Ks codon-level technical error (Ks = 0 for 2,487/2,972 top mutations), we combined methodologies and identified 69 top nonsynonymous mutations in TR (c/μ > 3 + Ka/Ks > 2.5) with > 83-fold reduced false positive counts, 107 top synonymous mutations in TR (c/μ > 3 + Ks/μ > 3) and 11 top mutations in UTR (c/μ > 3) for the SARS-COV-2 genomic datasets in this study. Encouragingly, 70% (nonsynonymous TR) and 20% (UTR) of the top mutation effects were in excellent agreement with the literature, especially for three critical proteins (Spike, RdRp, and 3CL-PRO). Several reverse genetic engineering studies (Spike: 4; 3CL-PRO: 4; RdRp: 2) were also identified, which support the relationship between the genotype frequency (c/μ) and selection fitness due to an observed phenotype (e.g. viral infectivity), at least for SARS-COV-2 (Paradis and Wu 2024). In spike, 5 valid and 14 undefined Ka/Ks values (Ks = 0) out of 32 top nonsynonymous mutations (entire spike and receptor binding domain/RBD) were noted from the literature (N501Y (Liu et al. 2021), L452R (Tchesnokova et al. 2021), E484K (Ferrareze et al. 2021), T487K (Liu et al. 2021), S477N (Liu et al. 2022)), whereas our combined c/µ + Ka/Ks criteria correctly predicted all 32 mutations and 12 top mutations within spike RBD, which were 100% confirmed in the literature (D614G (Plante et al. 2021), P681H (Wang et al. 2021) R346K (Changrob et al. 2021), K417N (Gobeil et al. 2021), N439K (Schrors et al. 2021), N440K (Schrors et al. 2021), L452R (Tchesnokova et al. 2021), S477N (Gomez et al. 2022), T478K (Sanches et al. 2021), E484K (Ferrareze et al. 2021), F490S (Liu et al. 2021), and N501Y(Tian et al. 2021)). Existing literature reports that while the former mutations might increase RBD binding to ACE2 and lead to increased infectivity, the latter mutations might reduce antibody binding to RBD, leading to vaccine escape, such as D614G in a mouse model (c/μ = 68.67 and Ka/Ks = Undefined, Ks = 0) (Plante et al. 2021). For RdRp, 9 mutations were identified** (**A97V (Mohammad et al. 2021), A185V (Chand et al. 2020), P227L (Bravo et al. 2022), P323L (Pachetti et al. 2020), M666I (Kumar et al. 2022), G671S (Sarkar et al. 2021), and V776L (Kumar et al. 2022)) that could enhance viral infectivity. Increased RdRp-viral RNA binding complex stability for transcription and increase replication kinetics (Kim et al. 2023). Lastly for 3CL Protease, 4 mutations were identified (G15S, T21I, L89F, and K90R (Ullrich et al. 2022)) which slightly modulate drug resistance and enzyme efficiency GC376, S-217622 and PF-07321332 (Bei et al. 2023; Greasley et al. 2022). Moreover, the 107 top synonymous mutations in TR may play a role in RNA transcription or in codon optimization to improve translation, as was demonstrated for a codon-optimized SARS-COV-2 mRNA vaccine displaying > 130-fold potency in mice (Zhang et al. 2023). The full mutation analysis can be found in our report (Paradis and Wu 2024).

While some sites exhibited Ks = 0 using the c/μ + Ka/Ks method, c/μ provides a numerical threshold for a codon site to likely be under strong beneficial selection, so that its corresponding Ka/Ks value does not suffer from technical false positives. Moreover, since the new reference state (μ) is a non-zero number and is empirically determined from the SARS-COV-2 genomic datasets, not only does it avoid the technical error encountered by Ka/Ks, but the approximation of the mutation rate is actually quite good for identifying the top adaptive mutations under beneficial selection. We note that while our approximated μ value (the fastest segment substitution rate Orf1ab 5’UTR) may not represent the true mutation rate for SARS-COV-2, it is at the very least a lower boundary of μ for this virus. The upper boundary of µ can be further refined using the lowest c/µ value from the 51 experiment-determined adaptive mutations reported in the literature (see Fig. 5), where the true µ appears to be within 1–2.42 times the estimated µ.

Protein-coding TR are considered as more important than non-coding UTR and TRS in function from a protein-centric viewpoint, thus the inverse relationship from negative selection predicts TR sequence should be more conserved (c, _TR_ < c, _UTR_ or c, _TR_ < c, _TRS_) (de Jong et al. 2024). The former is correct ((c/µ_, TR_ (0.17) < c/µ_, UTR_ (0.53)) and the latter is wrong (c/µ_, TR_ (0.17) > c/µ_, TRS_ (0.07)), suggesting TRS play very important transcription regulation functions. From the protein-centric viewpoint, nonsynonymous mutation sites leading to protein sequence change are considered more important than synonymous mutation sites in function, thus the reverse relationship predicts nonsynonymous sites should be more conserved relative to synonymous sites (Ka < Ks) (de Jong et al. 2024). This prediction is true for the TR (Ka/µ (0.15) < Ks/µ (0.24)). From the protein-centric viewpoint, both UTR/TRS and synonymous sites that are not involved protein sequence change are less important, thus the reverse relationship predicts the substitution rate of synonymous mutation sites is close to the substitution rate of UTR (c, _UTR_ ≈ Ks_, TR_ or c, _TRS_ ≈ Ks). This prediction is not satisfied for the UTR (c/µ, _UTR_ (0.53) ≠ Ks/µ,_TR_ (0.24)) as well as the TRS (c/µ, _TRS_ (0.07) ≠ Ks/µ,_TR_ (0.24)). These irregularities suggest that more sophisticated tests such as c/μ, Ks/μ, and Ka/μ should be used alongside the conventional Ka/Ks ratio to better detect molecular adaptation and understand the functions of genomic segments. These advanced tests might provide more accurate insights into evolutionary pressures and functional roles of specific genes or genomic regions.

In this work, the c/µ analysis was applied to SARS-COV-2. By expanding the c/µ analysis to more viruses and even other model organisms with ample public data, one can evaluate the robustness of the proposed c/µ test and NNST theories across different species. Therefore, c/µ analysis can serve as a high-resolution tool to decipher evolution dynamics of a species using its dated genomic sequences in both temporal and sequence space, allowing it to rigorously evaluate different evolutionary theories toward resolving the longstanding debate in evolutionary biology. Nonetheless, a potential risk is our assumption of time-independent site-independent global genomic mutation rate (µ) for a virus. Although it appears to be true for SARS-COV-2, it remains to be validated for other species from the replication in vitro experiments without subjecting the selection. It is well known that mutation rates can vary across different regions of the genome for more complex organism like human (Hodgkinson and Eyre-Walker 2011) (Harris and Pritchard 2017) due to some local factors, such as differences in replication timing; sequence context (e.g. CpG sites mutate at higher rates); and chromatin structure or proximity to regulatory regions. If necessary, we will relax our assumption to take the variation of mutation rate into consideration. For example, CpG sites can take different mutation rates or different genes can take different mutation rates. In addition, the secondary structure of RNA or DNA will be used to adjust mutation rate through sequence-structure compatibility (Teufel et al. 2018) or/and structure stability (Dasmeh et al. 2014). To relax the assumption of the time-independent mutation rate, different lineages are allowed to take different rates (Bloom and Neher 2023). To summarize, our c/µ method currently applies to simple haploid systems such as RNA viruses, where a constant mutation rate across the genome and time can be safely assumed. Our future investigations aim to extend our c/µ method to higher organisms (i.e. bacteria, humans) by relaxing this assumption and allowing variations in the mutation rate across genomic space and time.

## Conclusion

In summary, this paper derives a general equation linking c/μ with weighted Ks/μ and Ka/μ (c/μ = Ps*(Ks/μ) + Pa*(Ka/μ), where Ps and Pa are the proportions of synonymous and nonsynonymous sites of a coding sequence under a choice of a Markov mutation model and a codon table. It demonstrates that Ka/Ks infers the same mutation type as c/μ only if synonymous mutations are neutral (Ks/µ = 1); otherwise, Ka/Ks might give different selection assignments. In a case study, the relative substitution ratios c/µ and Ka/Ks from the time-based and position-based approaches are consistent with each other, suggesting that the constancy of the rates (i.e. strict molecular clock) holds for most coding segments of SARS-COV-2. However, none of the 25 proteins exhibited a Ks/µ = 1, failing to satisfy the equivalency condition between c/µ and Ka/Ks. c/µ and Ka/Ks report the same type of fitness change for 18 out of the 25 proteins, thus Ka/Ks reports the incorrect type of fitness change by missing the fitness change due to synonymous mutations (Ks/µ) for the remaining 7 proteins. Thus, the c/µ test without assuming synonymous site neutrality, a specific Markov model and a specific codon table or limitation to protein-coding region provides a powerful tool to detect selection pressure. Nonetheless, unbiased determination of µ through comparative approach remains to be improved for different life domains.

## Electronic supplementary material

Below is the link to the electronic supplementary material.

Supplementary file1 (XLSX 3453 KB)—A pdf file includes Tables S1–S12 present detailed time-based and position-based analyses of SARS-CoV-2 substitution rates and ratios (e.g., c, c/μ, Ka, Ks, and Ka/Ks) across non-coding (UTR/TRS) and coding regions, aggregated over multiple datasets. Figures S1–S7 illustrate the replication–selection model (S1), depict total nucleotide substitution variation in UTR and TRS regions over time (S2, S3), show percent synonymous and non-synonymous codon changes across major and accessory genes (S4–S6), and compare trendlines for genomic substitution rates under different fitting approaches (S7)

Supplementary file2 (PDF 4431 KB)—An Excel file includes a summary of calculating generation time using literature data

## Data Availability

The SARS-COV-2 sequence metadata will be provided as a supporting document. The MATLAB code will be made available to readers upon request.

## Appendix A

To show the proof that the Ka/Ks ratio is a special case of the c/μ ratio test under the condition that $\text{Ks}=\upmu$, and if $\text{Ks}\ne\upmu$, Ka/Ks will miss the fitness effect due to silent nucleic acid change. We present six cases demonstrating the equivalency $\frac{{\text{c}}}{\mu } \Leftrightarrow \frac{{{\text{Ka}}}}{{{\text{Ks}}}}$ and $\frac{{{\text{Ka}}}}{{{\text{Ks}}}} \Leftrightarrow \frac{{\text{c}}}{\upmu }$ (3 cases each, 6 total cases), given Eq. 14 reduces down to Eq. 15 when $\text{Ks}=\upmu$,:

14 $$\frac{c}{\mu } = Pa*\left( {\frac{Ka}{{Ks}} + \frac{Ks}{\mu }} \right) + Ps*\left( {\frac{Ks}{\mu }} \right)$$

15 $$\frac{c}{\mu } = Pa*\left( {\frac{Ka}{{Ks}}} \right) + Ps$$

16 $$Pa + Ps = 1$$

where Pa and Ps are the fractions of nonsynonymous sites and synonymous sites/mutations under a mutation model and a codon table, respectively; Ka and Ks are the nonsynonymous and synonymous substitution rates, respectively.

## Case 1: $\frac{\mathbf{K}\mathbf{a}}{\mathbf{K}\mathbf{s}}=1\boldsymbol{ }({\varvec{N}}{\varvec{e}}{\varvec{u}}{\varvec{t}}{\varvec{r}}{\varvec{a}}{\varvec{l}}\boldsymbol{ }{\varvec{s}}{\varvec{e}}{\varvec{l}}{\varvec{e}}{\varvec{c}}{\varvec{t}}{\varvec{i}}{\varvec{o}}{\varvec{n}})$

When Ka = Ks for a codon site, this implies synonymous and nonsynonymous mutations are fixed at an equivalent rate, thus the site is under neutral selection.

*When* $\frac{\mathbf{K}\mathbf{a}}{\mathbf{K}\mathbf{s}}=1$ is substituted into 1b of c/μ:

17 $$\frac{c}{\mu } = Pa*1 + Ps$$

Ka and Ks terms cancel out, and c/μ becomes the sum of Pa and Ps. Simplifying further,

18 $$\frac{c}{\mu } = Pa + Ps = 1$$

Therefore, $$\frac{Ka}{{Ks}} = 1 \Leftrightarrow \frac{c}{\mu } = 1$$.

## Case 2: $\frac{\mathbf{K}\mathbf{a}}{\mathbf{K}\mathbf{s}}>1\boldsymbol{ }({\varvec{A}}{\varvec{d}}{\varvec{a}}{\varvec{p}}{\varvec{t}}{\varvec{i}}{\varvec{v}}{\varvec{e}}\boldsymbol{ }{\varvec{s}}{\varvec{e}}{\varvec{l}}{\varvec{e}}{\varvec{c}}{\varvec{t}}{\varvec{i}}{\varvec{o}}{\varvec{n}})$

When Ka is greater than Ks for a codon site, this implies that nonsynonymous mutations are being fixed at a higher rate than synonymous mutations, indicating this codon site is under adaptive selection.

19 $$\frac{c}{\mu } = Pa*\frac{Ka}{{Ks}} + Ps$$

Since $$\frac{{{\text{Ka}}}}{{{\text{Ks}}}} > 1$$. Therefore,

20 $$Pa*\frac{Ka}{{Ks}}\rangle Pa$$

When (4) is substituted into 1b:

21 $$\frac{c}{\mu }\rangle Pa + Ps = 1$$

## Case 3: $\frac{\mathbf{K}\mathbf{a}}{\mathbf{K}\mathbf{s}}<1({\varvec{P}}{\varvec{u}}{\varvec{r}}{\varvec{i}}{\varvec{f}}{\varvec{y}}{\varvec{i}}{\varvec{n}}{\varvec{g}}\boldsymbol{ }{\varvec{s}}{\varvec{e}}{\varvec{l}}{\varvec{e}}{\varvec{c}}{\varvec{t}}{\varvec{i}}{\varvec{o}}{\varvec{n}})$

When Ka is smaller than Ks for a codon site, this implies that synonymous mutations are being fixed at a higher rate than nonsynonymous mutations, indicating it is under purifying selection.

When substituting for $\frac{{\text{c}}}{\upmu } = {\text{Pa}}*\frac{{{\text{Ka}}}}{{{\text{Ks}}}} + {\text{Ps}}$ and simplifying further,

22 $$Pa*\frac{Ka}{{Ks}}\langle Pa$$

Since $$\frac{{{\text{Ka}}}}{{{\text{Ks}}}} < 1$$. Therefore,

23 $$\frac{c}{\mu }\langle Pa + Ps = 1$$

## Case 4: $\frac{\mathbf{c}}{{\varvec{\upmu}}}=1({\varvec{N}}{\varvec{e}}{\varvec{u}}{\varvec{t}}{\varvec{r}}{\varvec{a}}{\varvec{l}}\boldsymbol{ }{\varvec{s}}{\varvec{e}}{\varvec{l}}{\varvec{e}}{\varvec{c}}{\varvec{t}}{\varvec{i}}{\varvec{o}}{\varvec{n}})$

When c = μ, mutations are being fixed at a rate equivalent to the approximated mutation rate, indicating the codon site is under neutral selection.

By the expression:

24 $$\frac{c}{\mu } = Pa*\frac{Ka}{{Ks}} + Ps = 1$$

$Pa*\frac{Ka}{Ks}$ and $Ps$ contribute to the codon site under neutral selection. By algebraic manipulation of Eq. 8,

25 $$Pa*\frac{Ka}{{Ks}} = 1 - Ps$$

And following the condition that $\text{Pa}+\text{Ps}=1$, then Eq. 9 simplifies to:

26 $$Pa*\frac{Ka}{{Ks}} = Pa$$

Dividing both sides by $Pa$, we obtain $\frac{{{\text{Ka}}}}{{{\text{Ks}}}} = 1$ when $\frac{\text{c}}{\upmu }=1$

## Case 5: $\frac{\mathbf{c}}{{\varvec{\upmu}}}>1\boldsymbol{ }({\varvec{A}}{\varvec{d}}{\varvec{a}}{\varvec{p}}{\varvec{t}}{\varvec{i}}{\varvec{v}}{\varvec{e}}\boldsymbol{ }{\varvec{s}}{\varvec{e}}{\varvec{l}}{\varvec{e}}{\varvec{c}}{\varvec{t}}{\varvec{i}}{\varvec{o}}{\varvec{n}})$

Similarly, when c > μ for a codon site, mutations are fixed at a higher rate than the approximated mutation rate, indicating the given codon site is under adaptive selection.

Hence,

27 $$Pa*\frac{Ka}{{Ks}} + Ps\rangle 1$$

When simplified,

$$Pa*\frac{Ka}{{Ks}}\rangle Pa$$

Dividing both sides by $\text{Pa}$, we obtain $\frac{{{\text{Ka}}}}{{{\text{Ks}}}} > 1$ when $\frac{\text{c}}{\upmu }>1$

## Case 6: $\frac{\mathbf{c}}{{\varvec{\upmu}}}<1\boldsymbol{ }({\varvec{P}}{\varvec{u}}{\varvec{r}}{\varvec{i}}{\varvec{f}}{\varvec{y}}{\varvec{i}}{\varvec{n}}{\varvec{g}}\boldsymbol{ }{\varvec{s}}{\varvec{e}}{\varvec{l}}{\varvec{e}}{\varvec{c}}{\varvec{t}}{\varvec{i}}{\varvec{o}}{\varvec{n}})$

When c < μ for a codon site, mutations are fixed at a lower rate than the approximated mutation rate, indicating the given codon site is under purifying selection.

Hence,

28 $$Pa*\frac{Ka}{{Ks}} + Ps < 1$$

When simplified,

$$Pa*\frac{Ka}{{Ks}}\langle Pa$$

Dividing both sides by $\text{Pa}$, we obtain $\frac{{{\text{Ka}}}}{{{\text{Ks}}}} < 1$ when $\frac{\text{c}}{\upmu }<1$
