# Supplementary material for: Substitution-Mutation Rate Ratio (c/µ) As Molecular Adaptation Test Beyond Ka/Ks: A SARS-COV-2 Case Study
Source: J Mol Evol. 2025 May 3;93(3):322–49. doi: 10.1007/s00239-025-10248-6 (PMC12198311; doi:10.1007/s00239-025-10248-6)
Supplement: Supplementary file 2 — Supplementary file2 (PDF 4431 KB)—An Excel file includes a summary of calculating generation time using literature data [file 239_2025_10248_MOESM2_ESM.pdf]

Table S1. Time-based c total substitution rate slope for each dataset and averaged over each dataset for all SARS-COV-2 UTR and TRS segments.

Seg (NT Length)	A1a c	A1b c	A1c c	Average c
**All UTR (771)**	18.50	18.80	19.80	19.00±0.68
**All TRS (61)**	2.30	2.50	2.60	2.47±0.15
**Orf1ab 5'UTR (265)**	35.40	35.90	36.90	36.10±0.76
**Orf1ab TRS-L (7)**	0.00	0.20	1.40	0.53±0.76
**S 5'UTR (7)**	0.70	0.20	0.30	0.40±0.27
**S TRS-B (7)**	0.70	0.00	0.00	0.23±0.40
**E 5'UTR (24)**	1.20	1.50	1.50	1.40±0.17
**E TRS-B (6)**	0.00	0.00	0.00	0.00±0.00
**M 5'UTR (50)**	1.70	2.20	2.20	2.03±0.29
**M TRS-B (7)**	0.00	0.00	0.00	0.00±0.00
**N 5'UTR (14)**	24.70	28.30	29.70	27.60±2.58
**N TRS-B (7)**	1.10	1.10	0.80	1.00±0.17
**Orf3a 5'UTR (8)**	0.00	0.00	0.30	0.10±0.17
**Orf3a TRS-B (7)**	0.70	0.70	0.40	0.60±0.17
**Orf6 5'UTR (10)**	1.40	0.70	1.90	1.33±0.60
**Orf6 TRS-B (6)**	0.80	1.60	0.40	0.93±0.61
**Orf7a 5'UTR (6)**	12.40	10.10	8.90	10.50±1.78
**Orf7a TRS-B (7)**	10.60	8.70	7.60	8.97±1.52
**Orf8 5'UTR (134)**	8.00	8.70	8.40	8.37±0.35
**Orf8 TRS-B (7)**	6.40	9.80	12.0	9.40±2.82
**Orf10 5'UTR (24)**	12.40	11.60	8.40	10.80±2.12
**Orf10 3'UTR (229)**	12.80	12.70	15.40	13.60±1.53

c = xE-03% / NT site / month.
μ = total Orf1ab 5'UTR substitution rate = 36.61E-03% substitutions / NT site / month.

Table S2. Time-based total genomic substitution rate slope for each dataset and averaged over each dataset for all SARS-COV-2 coding proteins.

Seg (NT Length)	A1a c	A1b c	A1c c	Average c	Gene/Protein Function
**Genome (29,903)**	6.40	6.90	6.50	6.60±0.26	Encodes virus components
**All-TR (29,133)**	6.10	6.10	6.10	6.10±0.00	Protein-coding region
**Orf1ab (21,291)**	4.00	4.00	4.10	4.03±0.06	Polyprotein; contains NSP1-15
**S (3,822)**	10.10	10.40	10.20	10.20±0.15	Glycoprotein; introduces host cell infection
**E (228)**	2.70	2.80	2.70	2.73±0.06	Envelope; virus assembly/release, ion channel
**M (669)**	4.80	5.00	4.90	4.90±0.10	Membrane; virus assembly/budding
**N (1,260)**	23.20	23.3	23.00	2.32±0.15	Nucleocapsid; virus packing & self-assembly
**Orf3a (828)**	8.60	8.70	9.00	8.77±0.21	Inhibits autophagy & apoptosis
**Orf6 (186)**	2.90	2.80	3.10	2.93±0.15	Inhibits nucleus molecular trafficking
**Orf7a (366)**	8.40	8.90	8.50	8.60±0.27	Downregulates antiviral activity
**Orf8 (366)**	22.20	21.90	22.40	22.20±0.25	Modulates viral replication/host immunity
**Orf10 (117)**	4.40	4.30	4.40	4.37±0.06	N/A
**Nsp1 (538)**	3.00	3.10	3.20	3.10±0.10	Promotes viral mRNA translation
**Nsp2 (1,912)**	4.00	4.00	4.00	4.00±0.00	Promotes viral RNA synthesis & translation
**Nsp3 (5,388)**	5.00	5.00	5.00	5.00±0.00	3CL-like protease; cleaves ORF1ab
**Nsp4 (1,498)**	3.90	4.00	4.10	4.00±0.10	Mediates building of replication organelle
**Nsp5 (916)**	2.70	2.60	3.20	2.83±0.32	Main protease; cleaves Orf1ab
**Nsp6 (868)**	5.30	5.40	5.10	5.27±0.15	Mediates building of replication organelle
**Nsp7 (247)**	2.40	2.30	2.30	2.33±0.06	Cofactor of NSP11/RdRp
**Nsp8 (592)**	1.30	1.30	1.50	1.37±0.12	Cofactor & stabilizer of NSP11
**Nsp9 (337)**	4.30	4.60	4.40	4.43±0.15	Binds viral ssRNA for nuclear transport
**Nsp10 (415)**	1.30	1.20	1.10	1.20±0.10	Cofactor & stimulator of NSP13 & NSP14
**Nsp11 (2,794)**	6.30	6.30	6.20	6.27±0.06	RdRp; synthesizes RNA genome
**Nsp12 (1,801)**	2.80	2.90	2.80	2.83±0.06	Helicase; unwinds viral RNA for transcription
**Nsp13 (1,579)**	2.70	2.70	2.80	2.73±0.06	Exoribonuclease; proofreading machinery
**Nsp14 (1,036)**	2.40	2.40	2.30	2.37±0.06	Proofreading exonuclease
**Nsp15 (892)**	2.10	2.00	2.30	2.13±0.15	2’-O-methyltransferase; immune evasion

c = xE-03% / NT site / month.

Table S3. Position-based c/μ (c/μ[a]), time-based c/μ (c/μ[b]) and their absolute difference values for the All-UTR, All-TRS and each UTR and TRS over 19 months, combined from datasets A1A-A1C. Bolded R^2 values exhibit good molecular clock features.

Seg (NT Length)	c/μ[a]	c/μ[b]	|Abs Difference|	|%Difference|	c R^2
**All UTR (771)**	0.52(H-)	0.53(H-)	0.01	1.74	**0.7167**
**All TRS (61)**	0.07(H-)	0.07(H-)	0.01	8.11	**0.6299**
**Orf1ab 5'UTR (265)**	0.97(H-)	1.00(H)	0.03	3.30	**0.9392**
**Orf1ab TRS-L (7)**	0.01(L-)	0.01(L-)	0.01	50.00	0.2699
**S 5'UTR (7)**	0.01(L-)	0.01(L-)	0.00	16.67	0.0239
**S TRS-B (7)**	0.01(L-)	0.01(L-)	0.00	0.00	-0.0075
**E 5'UTR (24)**	0.05(L-)	0.04(L-)	0.01	19.05	0.0399
**E TRS-B (6)**	0.00(L-)	0.00(L-)	0.00	0.00	N/A
**M 5'UTR (50)**	0.06(L-)	0.06(L-)	0.01	9.68	0.4925
**M TRS-B (7)**	0.00(L-)	0.00(L-)	0.00	0.00	N/A
**N 5'UTR (14)**	0.90(L-)	0.77(L-)	0.13	16.99	0.5577
**N TRS-B (7)**	0.03(L-)	0.03(L-)	0.01	26.67	0.2535
**Orf3a 5'UTR (8)**	0.00(L-)	0.00(L-)	0.00	50.00	-0.0235
**Orf3a TRS-B (7)**	0.01(L-)	0.02(L-)	0.01	55.56	-0.0402
**Orf6 5'UTR (10)**	0.03(L-)	0.04(L-)	0.01	15.00	0.2871
**Orf6 TRS-B (6)**	0.03(L-)	0.03(L-)	0.01	21.43	-0.2110
**Orf7a 5'UTR (6)**	0.34(L-)	0.29(L-)	0.05	15.72	0.2692
**Orf7a TRS-B (7)**	0.29(L-)	0.25(L-)	0.04	15.44	0.3129
**Orf8 5'UTR (134)**	0.23(L-)	0.23(L-)	0.00	0.79	0.4862
**Orf8 TRS-B (7)**	0.27(L-)	0.26(L-)	0.01	4.93	0.4698
**Orf10 5'UTR (24)**	0.27(L-)	0.30(L-)	0.03	9.76	0.2323
**Orf10 3'UTR (229)**	0.38(L-)	0.38(L-)	0.00	0.48	0.1415

***Low coefficient of determination causes significant deviation in c/μ[a].**
μ = total Orf1ab 5'UTR substitution rate.

Table S4. Time-based c/μ (c/μ^a), Position-based c/μ (c/μ^b), their absolute and percent differences for the genome, All-UTR, All-TR and each coding segment of SARS-COV-2 over 19 months, combined from datasets A1A-A1C. Bolded R^2 values exhibit good molecular clock features.

Seg (NT Length)	c/μ^a	c/μ^b	|Abs Difference|	|%Difference|	c R^2
**Genome (29,903)**	0.18(H-)	0.18(H-)	0.00	0.00	**0.9957**
**All-UTR (771)**	0.51(H-)	0.52(H-)	0.01	2.12	**0.7167**
**All-TRS (61)**	0.07(H-)	0.07(H-)	0.00	5.00	**0.6299**
**All-TR (29,133)**	0.17(H-)	0.17(H-)	0.01	3.16	**0.9805**
**Orf1ab (21,291)**	0.11(H-)	0.11(H-)	0.00	0.00	**0.9854**
**S (3,822)**	0.28(H-)	0.31(H-)	0.02	7.74	**0.8711**
**E (228)**	0.08(H-)	0.07(H-)	0.01	12.50	**0.7201**
**M (669)**	0.12(H-)	0.13(H-)	0.01	5.63	**0.7902**
**N (1,260)**	0.66(H-)	0.66(H-)	0.00	0.55	**0.9497**
**Orf3a (828)**	0.24(H-)	0.23(H-)	0.02	7.26	**0.6116**
**Orf6 (186)**	0.09(H-)	0.07(H-)	0.02	23.68	**0.0000**
**Orf7a (366)**	0.16(L-)	0.26(L-)	0.10	38.57	0.4031
**Orf8 (366)**	0.68(H-)	0.69(H-)	0.00	0.53	**0.6135**
**Orf10 (117)**	0.14(L-)	0.11(L-)	0.03	31.03	0.3557
**Nsp1 (538)**	0.10(H-)	0.08(H-)	0.01	18.18	**0.6735**
**Nsp2 (1,912)**	0.12(H-)	0.11(H-)	0.01	6.56	**0.8517**
**Nsp3 (5,388)**	0.14(H-)	0.14(H-)	0.00	1.28	**0.9801**
**Nsp4 (1,498)**	0.09(H-)	0.11(H-)	0.02	19.35	**0.6555**
**Nsp5 (916)**	0.09(L-)	0.07(L-)	0.02	23.08	0.5965
**Nsp6 (868)**	0.12(H-)	0.15(H-)	0.02	16.25	**0.6016**
**Nsp7 (247)**	0.07(L-)	0.05(L-)	0.02	30.00	0.3018
**Nsp8 (592)**	0.04(H-)	0.04(H-)	0.01	15.00	**0.8214**
**Nsp9 (337)**	0.12(H-)	0.13(H-)	0.01	9.72	**0.7971**
**Nsp10 (415)**	0.03(H-)	0.03(H-)	0.00	5.88	**0.7922**
**Nsp11 (2,794)**	0.18(H-)	0.18(H-)	0.00	0.00	**0.9169**
**Nsp12 (1,801)**	0.08(H-)	0.08(H-)	0.00	2.33	**0.9507**
**Nsp13 (1,579)**	0.07(H-)	0.07(H-)	0.00	2.63	**0.6755**
**Nsp14 (1,036)**	0.07(L-)	0.06(L-)	0.01	15.15	0.4841
**Nsp15 (892)**	0.06(H-)	0.05(H-)	0.01	20.69	**0.6361**

***Low coefficient of determination causes significant deviation in c/μ^a.**
μ = total Orf1ab 5'UTR substitution rate

Table S5. Time-based c/μ values for each dataset and averaged over each dataset for all SARS-COV-2 non-coding UTRs and TRSs.

Seg (NT Length)	A1a c/μ	A1b c/μ	A1c c/μ	Average c/μ
**All UTR (771)**	0.51	0.52	0.55	0.53±0.02(-)
**All TRS (61)**	0.06	0.07	0.07	0.07±0.00(-)
**Orf1ab 5'UTR (265)**	0.98	1.00	1.02	1.00±0.02(+)
**Orf1ab TRS-L (7)**	0.00	0.01	0.04	0.01±0.02(-)
**S 5'UTR (7)**	0.02	0.01	0.01	0.01±0.01(-)
**S TRS-B (7)**	0.02	0.00	0.00	0.01±0.01(-)
**E 5'UTR (24)**	0.03	0.04	0.04	0.04±0.00(-)
**E TRS-B (6)**	0.00	0.00	0.00	0.00±0.00(-)
**M 5'UTR (50)**	0.05	0.06	0.06	0.06±0.01(-)
**M TRS-B (7)**	0.00	0.00	0.00	0.00±0.00(-)
**N 5'UTR (14)**	0.69	0.79	0.82	0.77±0.07(-)
**N TRS-B (7)**	0.03	0.03	0.02	0.03±0.00(-)
**Orf3a 5'UTR (8)**	0.00	0.00	0.01	0.00±0.00(-)
**Orf3a TRS-B (7)**	0.02	0.02	0.01	0.02±0.00(-)
**Orf6 5'UTR (10)**	0.04	0.02	0.05	0.04±0.02(-)
**Orf6 TRS-B (6)**	0.02	0.04	0.01	0.03±0.02(-)
**Orf7a 5'UTR (6)**	0.34	0.28	0.25	0.29±0.05(-)
**Orf7a TRS-B (7)**	0.29	0.24	0.21	0.25±0.04(-)
**Orf8 5'UTR (134)**	0.22	0.24	0.23	0.23±0.01(-)
**Orf8 TRS-B (7)**	0.18	0.27	0.33	0.26±0.08(-)
**Orf10 5'UTR (24)**	0.34	0.32	0.23	0.30±0.06(-)
**Orf10 3'UTR (229)**	0.36	0.35	0.43	0.38±0.04(-)

***μ = 36.61E-03% substitutions / NT site / month.**

Table S6. Time-based c/μ values for each dataset and averaged over each dataset for all SARS-COV-2 coding proteins.

Seg (NT Length)	A1a c/μ	A1b c/μ	A1c c/μ	Average c/μ
**Genome (29,903)**	0.18	0.19	0.18	0.18±0.01(-)
**All-UTR (771)**	0.51	0.52	0.55	0.53±0.02(-)
**All-TRS (61)**	0.06	0.07	0.07	0.07±0.00(-)
**All-TR (29,133)**	0.17	0.17	0.17	0.17±0.00(-)
**Orf1ab (21,291)**	0.11	0.11	0.11	0.11±0.00(-)
**S (3,822)**	0.28	0.29	0.28	0.28±0.00(-)
**E (228)**	0.07	0.08	0.07	0.08±0.00(-)
**M (669)**	0.13	0.14	0.14	0.14±0.00(-)
**N (1,260)**	0.64	0.65	0.64	0.64±0.00(-)
**Orf3a (828)**	0.24	0.24	0.25	0.24±0.01(-)
**Orf6 (186)**	0.08	0.08	0.09	0.08±0.00(-)
**Orf7a (366)**	0.23	0.25	0.24	0.24±0.01(-)
**Orf8 (366)**	0.62	0.61	0.62	0.62±0.01(-)
**Orf10 (117)**	0.12	0.12	0.12	0.12±0.00(-)
**Nsp1 (538)**	0.08	0.09	0.09	0.09±0.00(-)
**Nsp2 (1,912)**	0.11	0.11	0.11	0.11±0.00(-)
**Nsp3 (5,388)**	0.14	0.14	0.14	0.14±0.00(-)
**Nsp4 (1,498)**	0.11	0.11	0.11	0.11±0.00(-)
**Nsp5 (916)**	0.07	0.07	0.09	0.08±0.01(-)
**Nsp6 (868)**	0.15	0.15	0.14	0.15±0.00(-)
**Nsp7 (247)**	0.07	0.06	0.06	0.06±0.00(-)
**Nsp8 (592)**	0.04	0.04	0.04	0.04±0.00(-)
**Nsp9 (337)**	0.12	0.13	0.12	0.12±0.00(-)
**Nsp10 (415)**	0.04	0.03	0.03	0.03±0.00(-)
**Nsp11 (2,794)**	0.17	0.17	0.17	0.17±0.00(-)
**Nsp12 (1,801)**	0.08	0.08	0.08	0.08±0.00(-)
**Nsp13 (1,579)**	0.07	0.07	0.08	0.08±0.00(-)
**Nsp14 (1,036)**	0.07	0.07	0.06	0.07±0.00(-)
**Nsp15 (892)**	0.06	0.06	0.06	0.06±0.00(-)

***μ = 36.61E-03% substitutions / NT site / month.**

Table S7. Time-based Ka/Ks (Ka/Ks[a]), position-based Ka/Ks (Ka/Ks[b]), their absolute and percent differences, Ka R^2 and Ks R^2 for All-TR and each coding segment of SARS-COV-2 over 19 months, combined from Datasets A1A-A1C. Bolded R^2 values exhibit good molecular clock features.

Seg (NT Length)	Ka/Ks[a]	Ka/Ks[b]	|Abs Difference|	|%Difference|	Ka R^2	Ks R^{2*}
**All-TR (29,133)**	0.62(-)	0.70(-)	0.08	11.43	**0.9838**	**0.9877**
**Orf1ab (21,291)**	0.32(-)	0.37(-)	0.05	12.73	**0.9929**	**0.9935**
**S (3,822)**	2.87(+)	3.23(+)	0.36	11.24	**0.9668**	**0.9252**
**E (228)**	1.74(+)	1.77(+)	0.03	1.51	**0.8859**	**0.6311**
**M (669)**	0.27(-)	0.40(-)	0.13	32.50	**0.7196**	**0.7504**
**N (1,260)**	1.03(+)	1.13(+)	0.10	9.12	**0.9964**	**0.9435**
**Orf3a (828)**	2.00(+)	2.27(+)	0.27	11.76	**0.9147**	**0.8250**
**Orf6 (186)**	0.32(-)	0.40(-)	0.08	20.00	0.5536	0.2133
**Orf7a (366)**	2.33(+)	3.40(+)	1.07	31.47	0.5531	**0.8782**
**Orf8 (366)**	1.48(+)	1.70(+)	0.22	12.94	**0.9148**	**0.8751**
**Orf10 (117)**	0.99(-)	1.03(+)	0.04	4.19	**0.8195**	0.4193
**Nsp1 (538)**	0.11(-)	0.10(-)	0.01	10.00	**0.7883**	**0.9457**
**Nsp2 (1,912)**	0.27(-)	0.23(-)	0.04	15.71	**0.8223**	**0.9565**
**Nsp3 (5,388)**	0.29(-)	0.30(-)	0.01	3.33	**0.9773**	**0.9715**
**Nsp4 (1,498)**	0.50(-)	0.57(-)	0.07	11.76	**0.8689**	**0.8574**
**Nsp5 (916)**	0.51(-)	0.47(-)	0.04	9.29	**0.9033**	**0.8970**
**Nsp6 (868)**	0.64(-)	0.67(-)	0.03	4.00	**0.8592**	**0.8552**
**Nsp7 (247)**	0.37(-)	0.27(-)	0.10	38.75	**0.6427**	**0.9118**
**Nsp8 (592)**	0.41(-)	0.37(-)	0.04	11.82	**0.9468**	**0.8704**
**Nsp9 (337)**	0.16(-)	0.10(-)	0.06	60.00	**0.9008**	**0.8671**
**Nsp10 (415)**	0.22(-)	0.20(-)	0.02	10.00	**0.9429**	**0.8393**
**Nsp11 (2,794)**	0.34(-)	0.33(-)	0.01	2.00	**0.9152**	**0.9336**
**Nsp12 (1,801)**	0.68(-)	0.80(-)	0.12	15.00	**0.9643**	**0.8870**
**Nsp13 (1,579)**	0.22(-)	0.30(-)	0.08	26.67	**0.9414**	**0.8406**
**Nsp14 (1,036)**	0.23(-)	0.20(-)	0.03	15.00	**0.8979**	**0.8330**
**Nsp15 (892)**	0.29(-)	0.30(-)	0.01	3.33	**0.8714**	**0.9166**

Table S8. Time-based Ka substitution rate slope and Ka R^2 values and position-based Ka substitution rate for each dataset and averaged over each dataset for all SARS-COV-2 coding proteins. Bolded R^2 values exhibit good molecular clock features.

Seg (NT Length)	Time-based							Position-based			
	A1a Ka	A1b Ka	A1c Ka	Average Ka	A1a Ka R2	A1b Ka R2	A1c Ka R2	A1a Ka	A1b Ka	A1c Ka	Average Ka
**All-TR (29,133)**	5.30	5.40	5.30	5.33±0.06	0.9821	0.9829	0.9856	4.23	5.08	3.62	4.31±0.74
**Orf1ab (21,291)**	2.70	2.70	2.80	2.73±0.06	0.9912	0.9914	0.9940	2.05	2.47	1.88	2.13±0.30
**S (3,822)**	11.7	12.1	16.1	13.3±2.43	0.9168	0.9249	0.9701	10.10	12.20	7.91	10.10±2.13
**E (228)**	3.50	3.40	4.40	3.77±0.55	0.8076	0.8344	0.8011	2.46	2.62	1.74	2.27±0.47
**M (669)**	2.30	2.40	3.70	2.80±0.78	0.6244	0.6460	0.8044	2.46	3.54	2.14	2.72±0.73
**N (1,260)**	23.80	24.40	29.80	26.00±3.30	0.9809	0.9795	0.9760	19.00	22.30	15.60	19.00±3.39
**Orf3a (828)**	9.80	9.90	10.10	9.93±0.15	0.9273	0.9053	0.8692	6.55	7.86	6.84	7.08±0.69
**Orf6 (186)**	1.70	2.30	2.00	2.00±0.30	0.6118	0.4371	0.4121	1.09	1.54	1.74	1.46±0.33
**Orf7a (366)**	6.30	6.40	10.6	7.77±2.45	0.5059	0.4586	0.6266	7.37	11.40	6.84	8.54±2.50
**Orf8 (366)**	19.10	19.30	24.90	21.10±3.29	0.8294	0.8308	0.8702	16.50	17.40	11.70	15.20±3.09
**Orf10 (117)**	4.70	4.40	4.20	4.43±0.25	0.6792	0.7411	0.7460	2.59	3.08	2.95	2.87±0.25
**Nsp1 (538)**	1.10	1.30	1.10	1.17±0.12	0.6750	0.7423	0.6331	0.68	0.92	0.80	0.80±0.12
**Nsp2 (1,912)**	2.80	2.70	2.60	2.70±0.10	0.8157	0.8175	0.8033	1.77	2.00	1.74	1.84±0.14
**Nsp3 (5,388)**	3.10	3.20	4.10	3.47±0.55	0.9414	0.9347	0.9705	2.59	3.08	2.14	2.61±0.47
**Nsp4 (1,498)**	2.50	2.70	3.60	2.93±0.59	0.8363	0.8287	0.9046	2.32	3.24	2.28	2.61±0.54
**Nsp5 (916)**	2.40	2.40	2.50	2.43±0.06	0.8734	0.9155	0.8620	1.36	1.69	1.74	1.60±0.21
**Nsp6 (868)**	3.90	4.00	4.80	4.23±0.49	0.8297	0.8247	0.8780	3.28	4.47	3.08	3.61±0.75
**Nsp7 (247)**	2.00	1.90	1.60	1.83±0.21	0.4957	0.6627	0.5261	0.82	1.08	1.07	0.99±0.15
**Nsp8 (592)**	1.10	1.00	1.10	1.07±0.06	0.9201	0.8841	0.8759	0.82	0.77	0.67	0.75±0.76
**Nsp9 (337)**	2.00	2.10	2.10	2.07±0.06	0.8649	0.8409	0.8224	1.36	1.39	1.07	1.27±0.18
**Nsp10 (415)**	0.70	0.60	0.70	0.67±0.06	0.7998	0.8042	0.8904	0.55	0.46	0.40	0.47±0.07
**Nsp11 (2,794)**	4.70	4.70	5.00	4.80±0.17	0.9124	0.9120	0.9158	3.28	4.01	3.22	3.50±0.44
**Nsp12 (1,801)**	2.40	2.40	2.90	2.57±0.29	0.9458	0.9389	0.9637	1.91	2.47	1.74	2.04±0.38
**Nsp13 (1,579)**	1.40	1.40	1.50	1.43±0.06	0.9342	0.9336	0.8955	1.09	1.39	1.21	1.23±0.15
**Nsp14 (1,036)**	1.50	1.40	1.50	1.47±0.06	0.8309	0.8481	0.8627	0.96	1.08	0.80	0.95±0.14
**Nsp15 (892)**	1.40	1.50	1.30	1.40±0.10	0.8178	0.8198	0.7579	0.82	0.92	1.07	0.94±0.13

***Ka substitution rate: xE-3% per NonSynonymous site per month.**

Table S9. Time-based Ks substitution rate slope and Ks R^2 values and position-based Ks substitution rate for each dataset and averaged over each dataset for all SARS-COV-2 coding proteins. Bolded R^2 values exhibit good molecular clock features.

Seg (NT Length)	Time-based							Position-based			
	A1a Ks	A1b Ks	A1c Ks	Average Ks	A1a Ks R2	A1b Ks R2	A1c Ks R2	A1a Ks	A1b Ks	A1c Ks	Average Ks
**All-TR (29,133)**	8.50	8.60	8.70	8.60±0.10	0.9892	0.9865	0.9859	6.01	6.93	5.36	6.10±0.79
**Orf1ab (21,291)**	8.40	8.50	8.60	8.50±0.10	0.9942	0.9927	0.9920	6.14	7.09	5.36	6.20±0.86
**S (3,822)**	4.60	4.60	4.70	4.63±0.06	0.9379	0.9173	0.8738	3.14	3.39	2.82	3.11±0.29
**E (228)**	2.00	2.50	2.10	22.00±0.27	0.5896	0.4230	0.3967	1.23	1.39	1.34	1.32±0.08
**M (669)**	10.90	11.40	9.40	10.60±1.04	0.7562	0.6971	0.5753	5.60	6.47	6.97	6.35±0.70
**N (1,260)**	24.80	24.10	26.90	25.30±1.46	0.9520	0.9435	0.8939	16.90	18.50	14.10	16.50±2.24
**Orf3a (828)**	5.20	5.00	4.70	4.97±0.25	0.8325	0.8117	0.7049	3.14	3.39	2.95	3.16±0.22
**Orf6 (186)**	8.10	6.00	5.60	6.57±1.34	0.1274	0.1178	0.4496	4.09	3.85	3.49	3.81±0.31
**Orf7a (366)**	2.90	3.60	3.50	3.33±0.38	0.8090	0.8515	0.7726	2.05	2.93	2.41	2.46±0.44
**Orf8 (366)**	14.10	13.90	14.70	14.20±0.42	0.8208	0.8663	0.8241	8.74	9.55	8.18	8.82±0.69
**Orf10 (117)**	4.30	5.20	4.10	4.53±0.59	0.5606	0.3216	0.1588	3.00	3.08	2.41	2.83±0.36
**Nsp1 (538)**	9.80	10.00	11.20	10.30±0.76	0.9302	0.9036	0.9164	6.14	7.86	6.30	6.77±0.95
**Nsp2 (1,912)**	9.20	9.60	12.00	10.30±1.51	0.9049	0.9249	0.9129	7.51	8.32	5.50	7.11±01.45
**Nsp3 (5,388)**	11.60	11.60	12.80	12.00±0.69	0.9793	0.9774	0.9497	8.05	9.40	7.51	8.32±0.97
**Nsp4 (1,498)**	5.40	5.50	6.60	5.83±0.67	0.8415	0.8375	0.8437	4.50	5.55	4.16	4.74±0.72
**Nsp5 (916)**	5.10	4.60	4.70	4.80±0.27	0.9031	0.8556	0.7918	3.14	3.24	3.22	3.20±0.05
**Nsp6 (868)**	6.30	6.10	7.40	6.60±0.70	0.8063	0.8177	0.8355	5.05	6.78	5.09	5.64±0.97
**Nsp7 (247)**	5.10	4.70	4.90	4.90±0.20	0.8371	0.8072	0.7729	3.55	3.70	2.95	3.40±0.40
**Nsp8 (592)**	2.40	2.80	2.70	2.63±0.21	0.8742	0.8135	0.7275	1.77	1.85	2.01	1.88±0.12
**Nsp9 (337)**	11.20	11.90	17.20	13.40±3.28	0.7719	0.7946	0.8243	10.50	14.00	8.58	11.00±2.76
**Nsp10 (415)**	3.10	3.40	2.80	3.10±0.30	0.6694	0.7184	0.7172	2.05	2.31	2.01	2.12±0.16
**Nsp11 (2,794)**	12.60	12.90	17.10	14.20±2.52	0.8660	0.8607	0.8795	10.80	11.70	7.64	10.00±2.13
**Nsp12 (1,801)**	3.60	3.90	3.90	3.80±0.17	0.8977	0.8711	0.8162	2.32	2.77	2.41	2.50±0.24
**Nsp13 (1,579)**	6.90	6.90	6.00	6.60±0.52	0.8468	0.8357	0.7224	3.96	4.31	4.02	4.10±0.19
**Nsp14 (1,036)**	6.30	6.50	6.10	6.30±0.20	0.8558	0.8004	0.7525	3.82	4.62	3.75	4.07±0.48
**Nsp15 (892)**	5.30	4.50	4.80	4.87±0.40	0.9100	0.8949	0.8651	3.14	3.24	2.95	3.11±0.15

***Ka substitution rate: xE-3% per Synonymous site per month.**

Table S10. Time-based Ka/μ values for each dataset and averaged over each dataset for all SARS-COV-2 coding proteins.

Seg (NT Length)	A1a Ka/μ	A1b Ka/μ	A1c Ka/μ	Average Ka/μ
**All-TR (29,133)**	0.15	0.15	0.15	0.15±0.00(-)
**Orf1ab (21,291)**	0.07	0.07	0.08	0.08±0.00(-)
**S (3,822)**	0.32	0.34	0.45	0.37±0.07(-)
**E (228)**	0.10	0.09	0.12	0.10±0.02(-)
**M (669)**	0.06	0.07	0.10	0.08±0.02(-)
**N (1,260)**	0.66	0.68	0.83	0.72±0.09(-)
**Orf3a (828)**	0.27	0.27	0.28	0.28±0.00(-)
**Orf6 (186)**	0.05	0.06	0.06	0.06±0.01(-)
**Orf7a (366)**	0.17	0.18	0.29	0.22±0.07(-)
**Orf8 (366)**	0.53	0.54	0.69	0.59±0.09(-)
**Orf10 (117)**	0.13	0.12	0.12	0.12±0.01(-)
**Nsp1 (538)**	0.03	0.04	0.03	0.03±0.00(-)
**Nsp2 (1,912)**	0.08	0.07	0.07	0.07±0.00(-)
**Nsp3 (5,388)**	0.09	0.09	0.11	0.10±0.02(-)
**Nsp4 (1,498)**	0.07	0.07	0.10	0.08±0.02(-)
**Nsp5 (916)**	0.07	0.07	0.07	0.07±0.00(-)
**Nsp6 (868)**	0.11	0.11	0.13	0.12±0.01(-)
**Nsp7 (247)**	0.06	0.05	0.04	0.05±0.01(-)
**Nsp8 (592)**	0.03	0.03	0.03	0.03±0.00(-)
**Nsp9 (337)**	0.06	0.06	0.06	0.06±0.00(-)
**Nsp10 (415)**	0.02	0.02	0.02	0.02±0.00(-)
**Nsp11 (2,794)**	0.13	0.13	0.14	0.13±0.00(-)
**Nsp12 (1,801)**	0.07	0.07	0.08	0.07±0.01(-)
**Nsp13 (1,579)**	0.04	0.04	0.04	0.04±0.00(-)
**Nsp14 (1,036)**	0.04	0.04	0.04	0.04±0.00(-)
**Nsp15 (892)**	0.04	0.04	0.04	0.04±0.00(-)

***μ = 36.61E-03% substitutions / NT site / month.**

Table S11. Time-based Ks/μ values for each dataset and averaged over each dataset for all SARS-COV-2 coding proteins.

Seg (NT Length)	A1a Ks/μ	A1b Ks/μ	A1c Ks/μ	Average Ks/μ
**All-TR (29,133)**	0.24	0.24	0.24	0.24±0.00(-)
**Orf1ab (21,291)**	0.24	0.24	0.24	0.24±0.00(-)
**S (3,822)**	0.13	0.13	0.13	0.13±0.00(-)
**E (228)**	0.07	0.06	0.06	0.06±0.01(-)
**M (669)**	0.32	0.26	0.29	0.29±0.01(-)
**N (1,260)**	0.67	0.75	0.70	0.70±0.01(-)
**Orf3a (828)**	0.14	0.13	0.14	0.14±0.00(-)
**Orf6 (186)**	0.17	0.16	0.18	0.18±0.04(-)
**Orf7a (366)**	0.10	0.10	0.09	0.09±0.01(-)
**Orf8 (366)**	0.39	0.41	0.39	0.39±0.00(-)
**Orf10 (117)**	0.14	0.11	0.13	0.13±0.02(-)
**Nsp1 (538)**	0.28	0.31	0.29	0.29±0.00(-)
**Nsp2 (1,912)**	0.27	0.33	0.28	0.28±0.01(-)
**Nsp3 (5,388)**	0.32	0.36	0.33	0.33±0.00(-)
**Nsp4 (1,498)**	0.15	0.18	0.16	0.16±0.00(-)
**Nsp5 (916)**	0.13	0.13	0.13	0.13±0.01(-)
**Nsp6 (868)**	0.17	0.21	0.18	0.18±0.00(-)
**Nsp7 (247)**	0.13	0.14	0.14	0.14±0.01(-)
**Nsp8 (592)**	0.08	0.07	0.07	0.07±0.01(-)
**Nsp9 (337)**	0.33	0.48	0.37	0.37±0.01(-)
**Nsp10 (415)**	0.09	0.08	0.09	0.09±0.01(-)
**Nsp11 (2,794)**	0.36	0.47	0.39	0.39±0.01(-)
**Nsp12 (1,801)**	0.11	0.11	0.11	0.11±0.01(-)
**Nsp13 (1,579)**	0.19	0.17	0.18	0.18±0.00(-)
**Nsp14 (1,036)**	0.18	0.17	0.17	0.17±0.00(-)
**Nsp15 (892)**	0.12	0.13	0.14	0.14±0.02(-)

***μ = 36.61E-03% substitutions / NT site / month.**

Table S12. Time-based Ka/Ks values for each dataset and averaged over each dataset for all SARS-COV-2 coding proteins.

Seg (NT Length)	Time-based				Position-based			
	A1a Ka/Ks	A1b Ka/Ks	A1c Ka/Ks	Average Ka/Ks	A1a Ka/Ks	A1b Ka/Ks	A1c Ka/Ks	Average Ka/Ks
**All-TR (29,133)**	0.62	0.63	0.61	0.62±0.01(-)	0.70	0.73	0.68	0.70±0.03(-)
**Orf1ab (21,291)**	0.32	0.32	0.33	0.32±0.00(-)	0.33	0.35	0.35	0.37±0.01(-)
**S (3,822)**	2.54	2.63	3.43	2.87±0.49(+)	3.22	3.59	2.81	3.23±0.39(+)
**E (228)**	1.75	1.36	2.10	1.74±0.37(+)	2.00	1.89	1.30	1.77±0.38(+)
**M (669)**	0.21	0.21	0.39	0.27±0.11(-)	0.44	0.55	0.31	0.40±0.12(-)
**N (1,260)**	0.96	1.01	1.11	1.03±0.08(+)	1.12	1.21	1.10	1.13±0.06(+)
**Orf3a (828)**	1.88	1.98	2.15	2.00±0.13(+)	2.09	2.32	2.32	2.27±0.13(+)
**Orf6 (186)**	0.21	0.38	0.36	0.32±0.09(-)	0.27	0.40	0.50	0.40±0.12(-)
**Orf7a (366)**	2.17	1.78	3.03	2.33±0.64(+)	3.60	3.89	2.83	3.40±0.55(+)
**Orf8 (366)**	1.35	1.39	1.69	1.48±0.19(+)	1.89	1.82	1.43	1.70±0.25(+)
**Orf10 (117)**	1.09	0.85	1.02	0.99±0.13(-)	0.86	1.00	1.22	1.03±0.18(+)
**Nsp1 (538)**	0.11	0.13	0.10	0.11±0.02(-)	0.11	0.12	0.13	0.10±0.01(-)
**Nsp2 (1,912)**	0.30	0.28	0.22	0.27±0.05(-)	0.24	0.24	0.32	0.23±0.04(-)
**Nsp3 (5,388)**	0.27	0.28	0.32	0.29±0.03(-)	0.32	0.33	0.29	0.30±0.02(-)
**Nsp4 (1,498)**	0.46	0.49	0.55	0.50±0.04(-)	0.52	0.58	0.55	0.57±0.03(-)
**Nsp5 (916)**	0.47	0.52	0.53	0.51±0.03(-)	0.43	0.52	0.54	0.47±0.06(-)
**Nsp6 (868)**	0.62	0.66	0.65	0.64±0.02(-)	0.65	0.66	0.61	0.67±0.03(-)
**Nsp7 (247)**	0.39	0.40	0.33	0.37±0.04(-)	0.23	0.29	0.36	0.27±0.07(-)
**Nsp8 (592)**	0.46	0.36	0.41	0.41±0.05(-)	0.46	0.42	0.33	0.37±0.07(-)
**Nsp9 (337)**	0.18	0.18	0.12	0.16±0.03(-)	0.13	0.10	0.13	0.10±0.12(-)
**Nsp10 (415)**	0.23	0.18	0.25	0.22±0.04(-)	0.27	0.20	0.20	0.20±0.04(-)
**Nsp11 (2,794)**	0.37	0.36	0.29	0.34±0.04(-)	0.30	0.34	0.42	0.33±0.06(-)
**Nsp12 (1,801)**	0.67	0.62	0.74	0.68±0.06(-)	0.82	0.89	0.72	0.80±0.08(-)
**Nsp13 (1,579)**	0.20	0.20	0.25	0.22±0.03(-)	0.28	0.32	0.30	0.30±0.02(-)
**Nsp14 (1,036)**	0.24	0.22	0.25	0.23±0.02(-)	0.25	0.23	0.21	0.20±0.02(-)
**Nsp15 (892)**	0.26	0.33	0.27	0.29±0.04(-)	0.26	0.29	0.36	0.30±0.05(-)

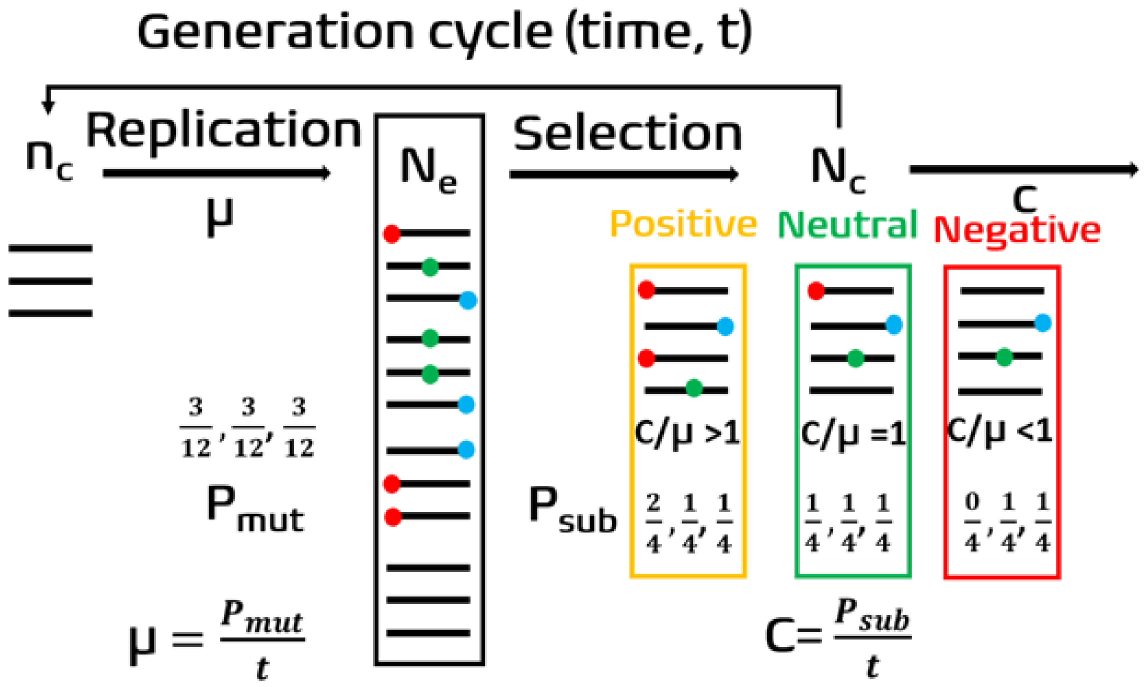

Figure S1. Simplified replication-selection model for a virus population over time under positive selection, neutral selection and negative selection for the first nucleotide position (red circle). A detailed description has been placed in the methods section. Viral genomes are shown as black lines. Mutations are shown as red, green and blue circles. *This figure was sourced from our previous paper.

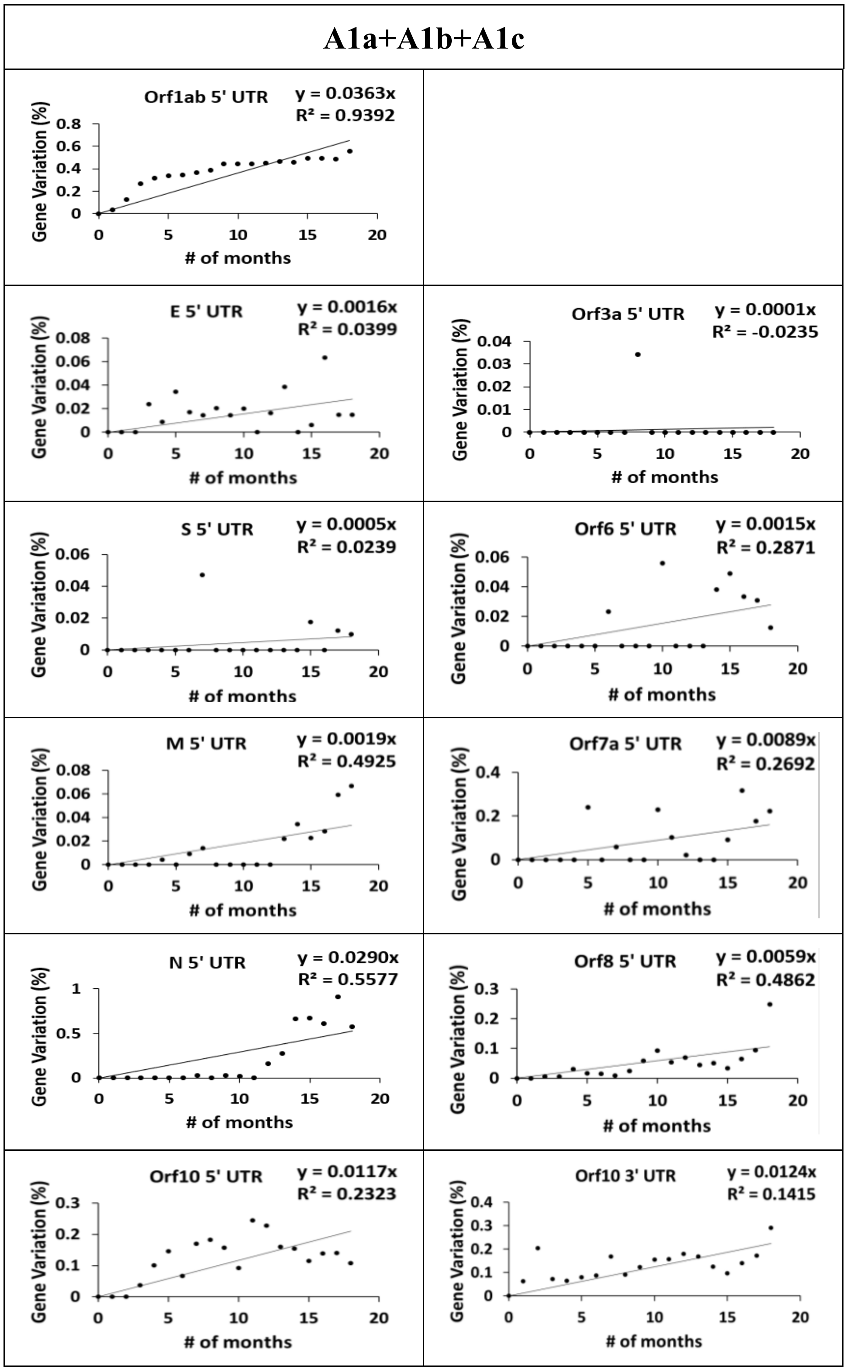

Figure S2. Total percent NT substitution variation for each UTR over evolution time averaged over the combined datasets. *This figure was sourced from our previous paper.

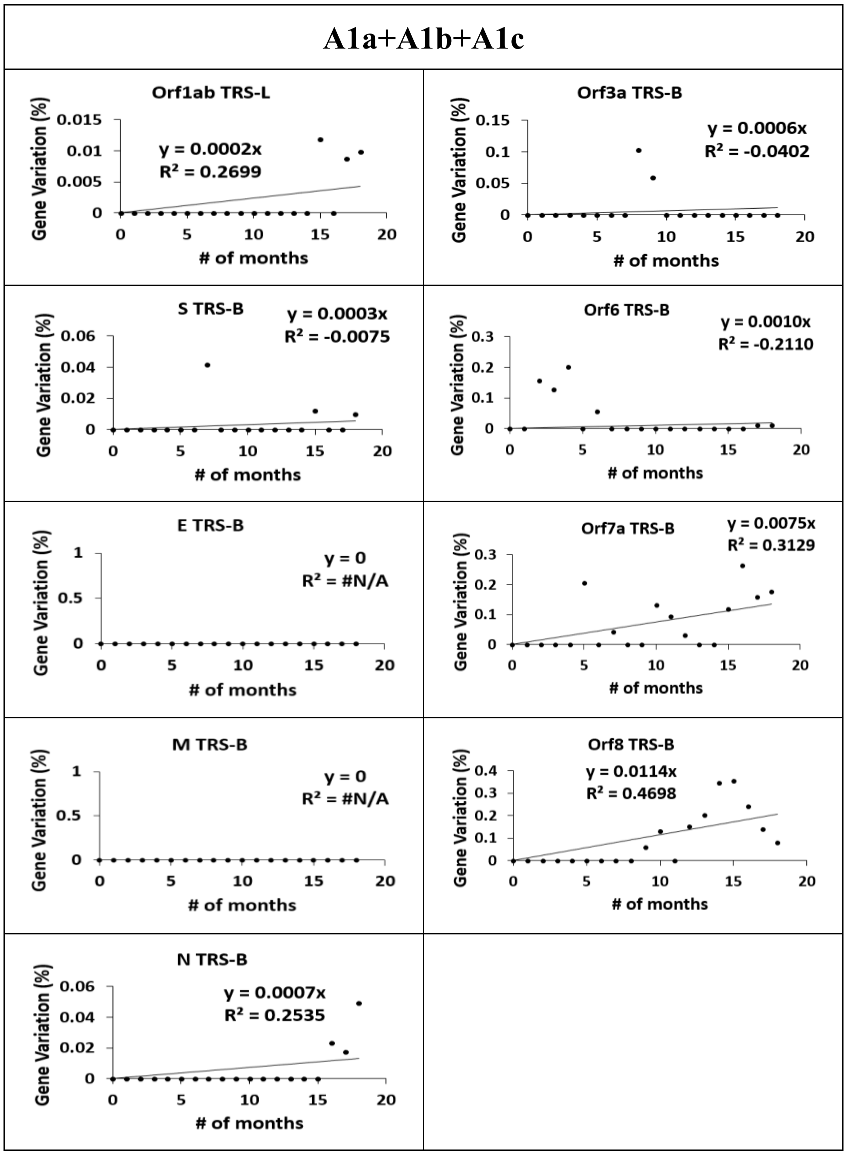

Figure S3. Total percent NT substitution variation for each TRS over evolution time averaged over the combined datasets. *This figure was sourced from our previous paper.

A1a+A1b+A1c

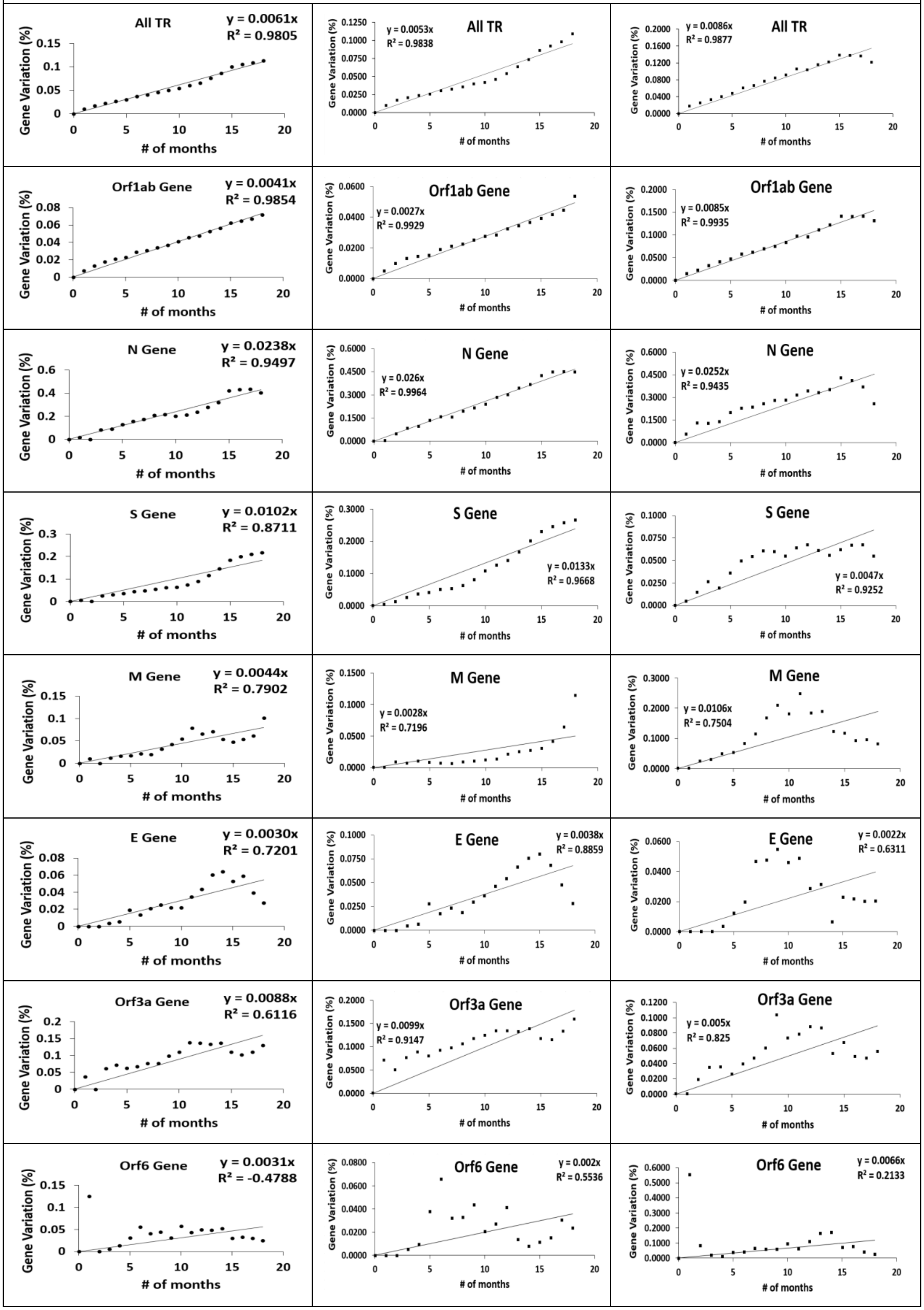
All TR
y = 0.0061x
R² = 0.9805
All TR
y = 0.0053x
R² = 0.9838
All TR
y = 0.0086x
R² = 0.9877
Orf1ab Gene
y = 0.0041x
R² = 0.9854
Orf1ab Gene
y = 0.0027x
R² = 0.9929
Orf1ab Gene
y = 0.0085x
R² = 0.9935
N Gene
y = 0.0238x
R² = 0.9497
N Gene
y = 0.026x
R² = 0.9964
N Gene
y = 0.0252x
R² = 0.9435
S Gene
y = 0.0102x
R² = 0.8711
S Gene
y = 0.0133x
R² = 0.9668
S Gene
y = 0.0047x
R² = 0.9252
M Gene
y = 0.0044x
R² = 0.7902
M Gene
y = 0.0028x
R² = 0.7196
M Gene
y = 0.0106x
R² = 0.7504
E Gene
y = 0.0030x
R² = 0.7201
E Gene
y = 0.0038x
R² = 0.8859
E Gene
y = 0.0022x
R² = 0.6311
Orf3a Gene
y = 0.0088x
R² = 0.6116
Orf3a Gene
y = 0.0099x
R² = 0.9147
Orf3a Gene
y = 0.005x
R² = 0.825
Orf6 Gene
y = 0.0031x
R² = -0.4788
Orf6 Gene
y = 0.002x
R² = 0.5536
Orf6 Gene
y = 0.0066x
R² = 0.2133
Gene Variation (%)
# of months

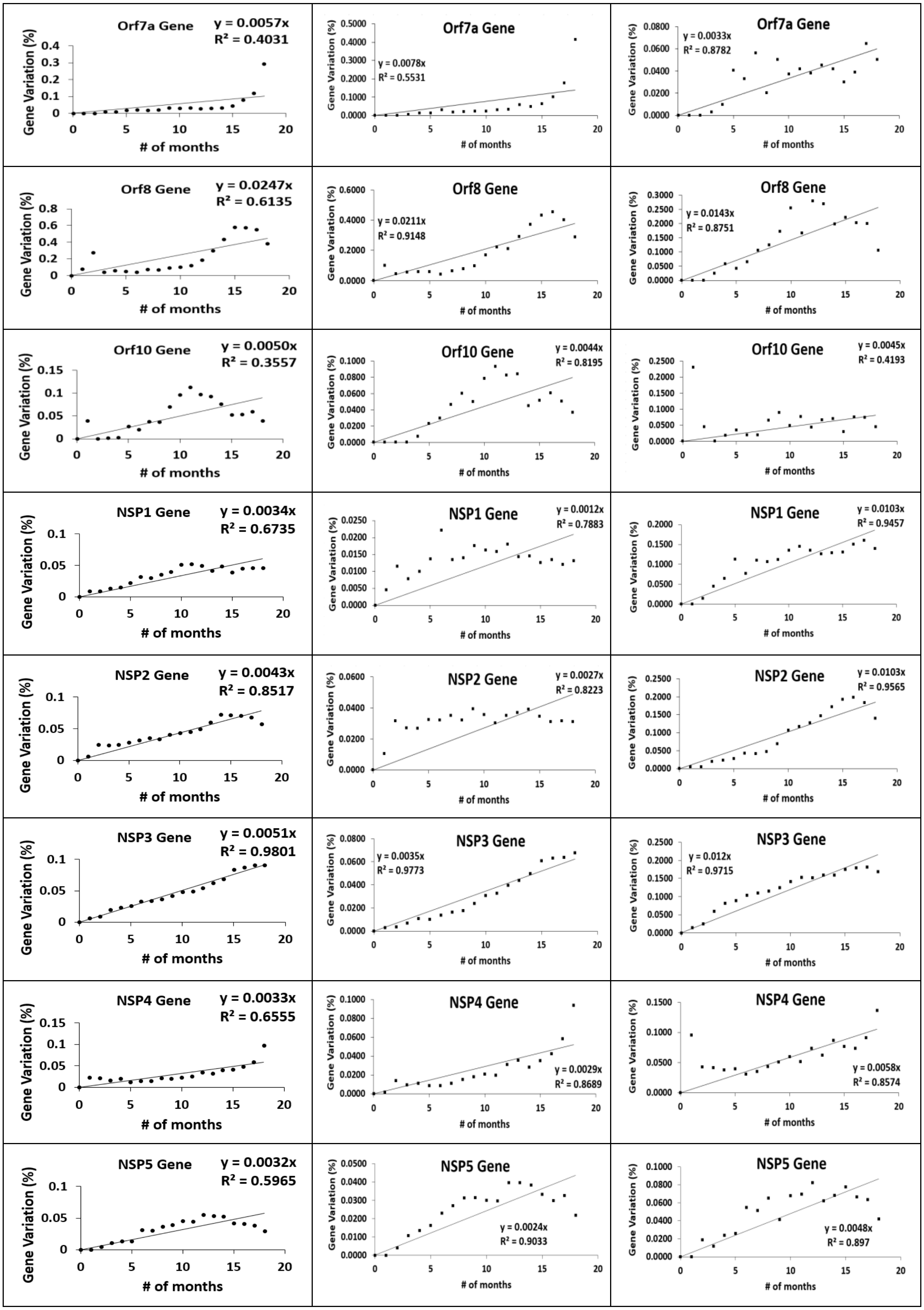
Orf7a Gene
y = 0.0057x
R² = 0.4031
Gene Variation (%)
# of months
Orf7a Gene
y = 0.0078x
R² = 0.5531
Gene Variation (%)
# of months
Orf7a Gene
y = 0.0033x
R² = 0.8782
Gene Variation (%)
# of months
Orf8 Gene
y = 0.0247x
R² = 0.6135
Gene Variation (%)
# of months
Orf8 Gene
y = 0.0211x
R² = 0.9148
Gene Variation (%)
# of months
Orf8 Gene
y = 0.0143x
R² = 0.8751
Gene Variation (%)
# of months
Orf10 Gene
y = 0.0050x
R² = 0.3557
Gene Variation (%)
# of months
Orf10 Gene
y = 0.0044x
R² = 0.8195
Gene Variation (%)
# of months
Orf10 Gene
y = 0.0045x
R² = 0.4193
Gene Variation (%)
# of months
NSP1 Gene
y = 0.0034x
R² = 0.6735
Gene Variation (%)
# of months
NSP1 Gene
y = 0.0012x
R² = 0.7883
Gene Variation (%)
# of months
NSP1 Gene
y = 0.0103x
R² = 0.9457
Gene Variation (%)
# of months
NSP2 Gene
y = 0.0043x
R² = 0.8517
Gene Variation (%)
# of months
NSP2 Gene
y = 0.0027x
R² = 0.8223
Gene Variation (%)
# of months
NSP2 Gene
y = 0.0103x
R² = 0.9565
Gene Variation (%)
# of months
NSP3 Gene
y = 0.0051x
R² = 0.9801
Gene Variation (%)
# of months
NSP3 Gene
y = 0.0035x
R² = 0.9773
Gene Variation (%)
# of months
NSP3 Gene
y = 0.012x
R² = 0.9715
Gene Variation (%)
# of months
NSP4 Gene
y = 0.0033x
R² = 0.6555
Gene Variation (%)
# of months
NSP4 Gene
y = 0.0029x
R² = 0.8689
Gene Variation (%)
# of months
NSP4 Gene
y = 0.0058x
R² = 0.8574
Gene Variation (%)
# of months
NSP5 Gene
y = 0.0032x
R² = 0.5965
Gene Variation (%)
# of months
NSP5 Gene
y = 0.0024x
R² = 0.9033
Gene Variation (%)
# of months
NSP5 Gene
y = 0.0048x
R² = 0.897
Gene Variation (%)
# of months

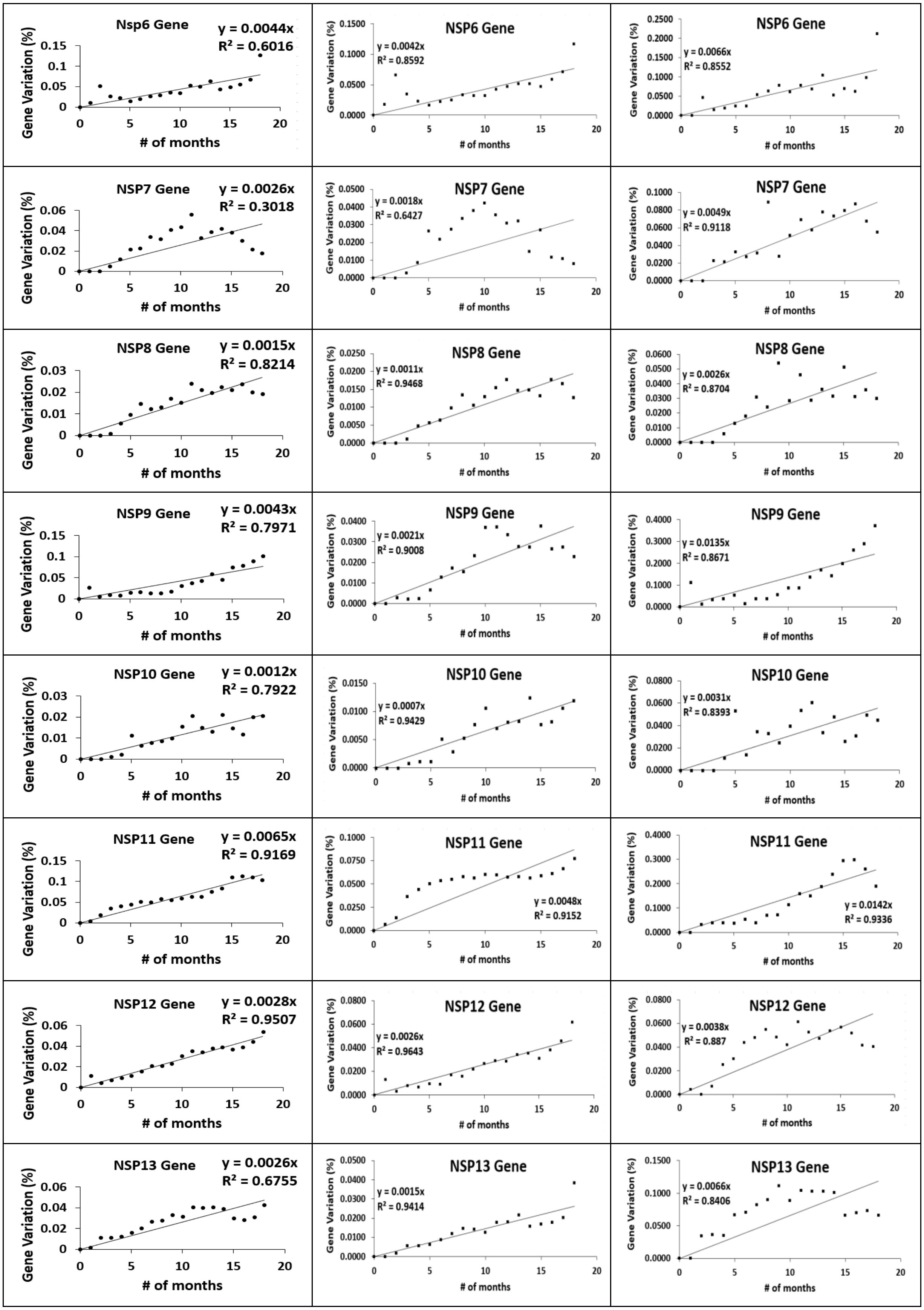

Nsp6 Gene
y = 0.0044x
R² = 0.6016
NSP6 Gene
y = 0.0042x
R² = 0.8592
NSP6 Gene
y = 0.0066x
R² = 0.8552
NSP7 Gene
y = 0.0026x
R² = 0.3018
NSP7 Gene
y = 0.0018x
R² = 0.6427
NSP7 Gene
y = 0.0049x
R² = 0.9118
NSP8 Gene
y = 0.0015x
R² = 0.8214
NSP8 Gene
y = 0.0011x
R² = 0.9468
NSP8 Gene
y = 0.0026x
R² = 0.8704
NSP9 Gene
y = 0.0043x
R² = 0.7971
NSP9 Gene
y = 0.0021x
R² = 0.9008
NSP9 Gene
y = 0.0135x
R² = 0.8671
NSP10 Gene
y = 0.0012x
R² = 0.7922
NSP10 Gene
y = 0.0007x
R² = 0.9429
NSP10 Gene
y = 0.0031x
R² = 0.8393
NSP11 Gene
y = 0.0065x
R² = 0.9169
NSP11 Gene
y = 0.0048x
R² = 0.9152
NSP11 Gene
y = 0.0142x
R² = 0.9336
NSP12 Gene
y = 0.0028x
R² = 0.9507
NSP12 Gene
y = 0.0026x
R² = 0.9643
NSP12 Gene
y = 0.0038x
R² = 0.887
NSP13 Gene
y = 0.0026x
R² = 0.6755
NSP13 Gene
y = 0.0015x
R² = 0.9414
NSP13 Gene
y = 0.0066x
R² = 0.8406
Gene Variation (%)
# of months

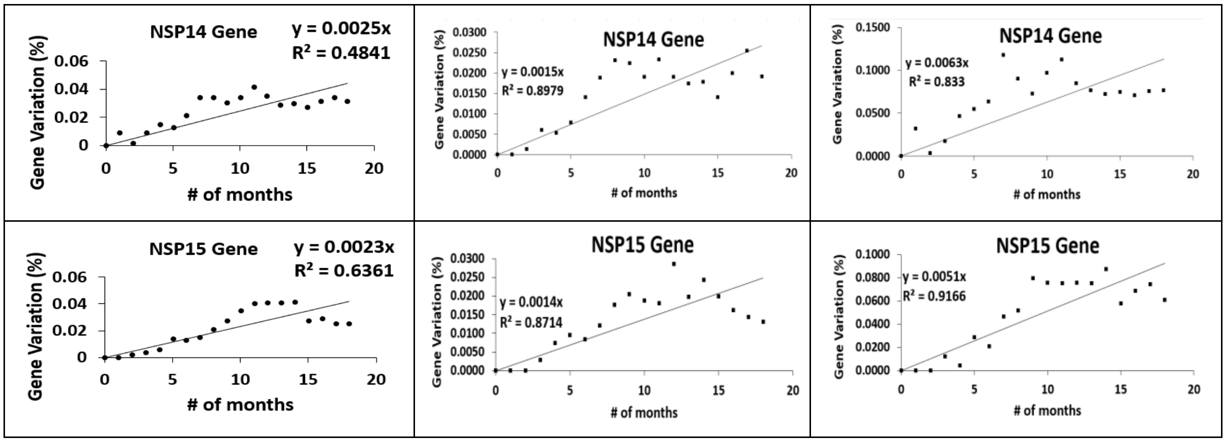

Figure S4. The percent sequence variation for each major gene and accessory gene over evolution time and averaged over the combined dataset. (**Column 1**) Percent total codon substitution rate. (**Column 2**) Percent non-synonymous codon substitution rate. (**Column 3**) Percent synonymous codon substitution rate.

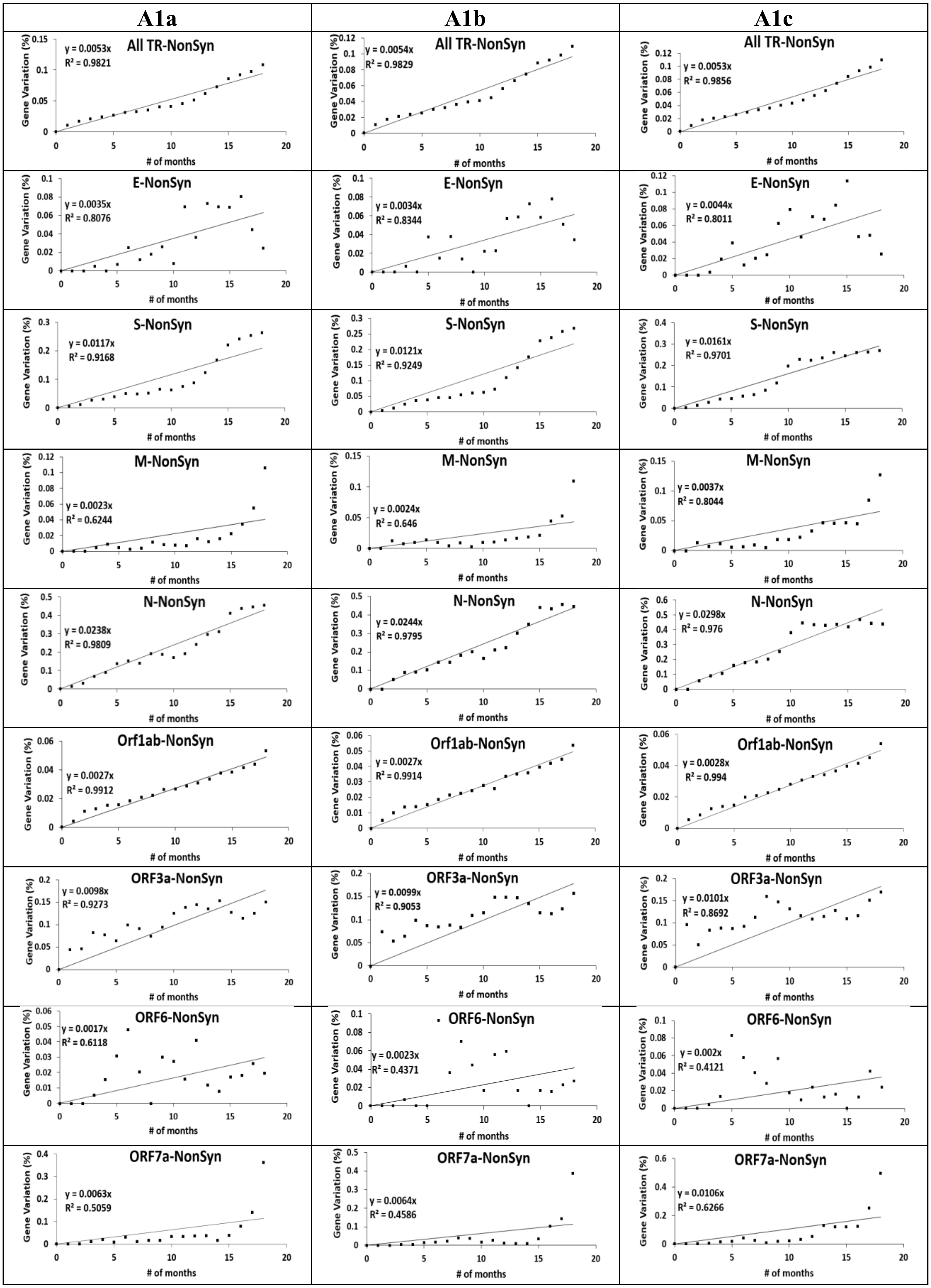
A1a
A1b
A1c
All TR-NonSyn
y = 0.0053x
R² = 0.9821
All TR-NonSyn
y = 0.0054x
R² = 0.9829
All TR-NonSyn
y = 0.0053x
R² = 0.9856
E-NonSyn
y = 0.0035x
R² = 0.8076
E-NonSyn
y = 0.0034x
R² = 0.8344
E-NonSyn
y = 0.0044x
R² = 0.8011
S-NonSyn
y = 0.0117x
R² = 0.9168
S-NonSyn
y = 0.0121x
R² = 0.9249
S-NonSyn
y = 0.0161x
R² = 0.9701
M-NonSyn
y = 0.0023x
R² = 0.6244
M-NonSyn
y = 0.0024x
R² = 0.646
M-NonSyn
y = 0.0037x
R² = 0.8044
N-NonSyn
y = 0.0238x
R² = 0.9809
N-NonSyn
y = 0.0244x
R² = 0.9795
N-NonSyn
y = 0.0298x
R² = 0.976
Orf1ab-NonSyn
y = 0.0027x
R² = 0.9912
Orf1ab-NonSyn
y = 0.0027x
R² = 0.9914
Orf1ab-NonSyn
y = 0.0028x
R² = 0.994
ORF3a-NonSyn
y = 0.0098x
R² = 0.9273
ORF3a-NonSyn
y = 0.0099x
R² = 0.9053
ORF3a-NonSyn
y = 0.0101x
R² = 0.8692
ORF6-NonSyn
y = 0.0017x
R² = 0.6118
ORF6-NonSyn
y = 0.0023x
R² = 0.4371
ORF6-NonSyn
y = 0.002x
R² = 0.4121
ORF7a-NonSyn
y = 0.0063x
R² = 0.5059
ORF7a-NonSyn
y = 0.0064x
R² = 0.4586
ORF7a-NonSyn
y = 0.0106x
R² = 0.6266
Gene Variation (%)
# of months

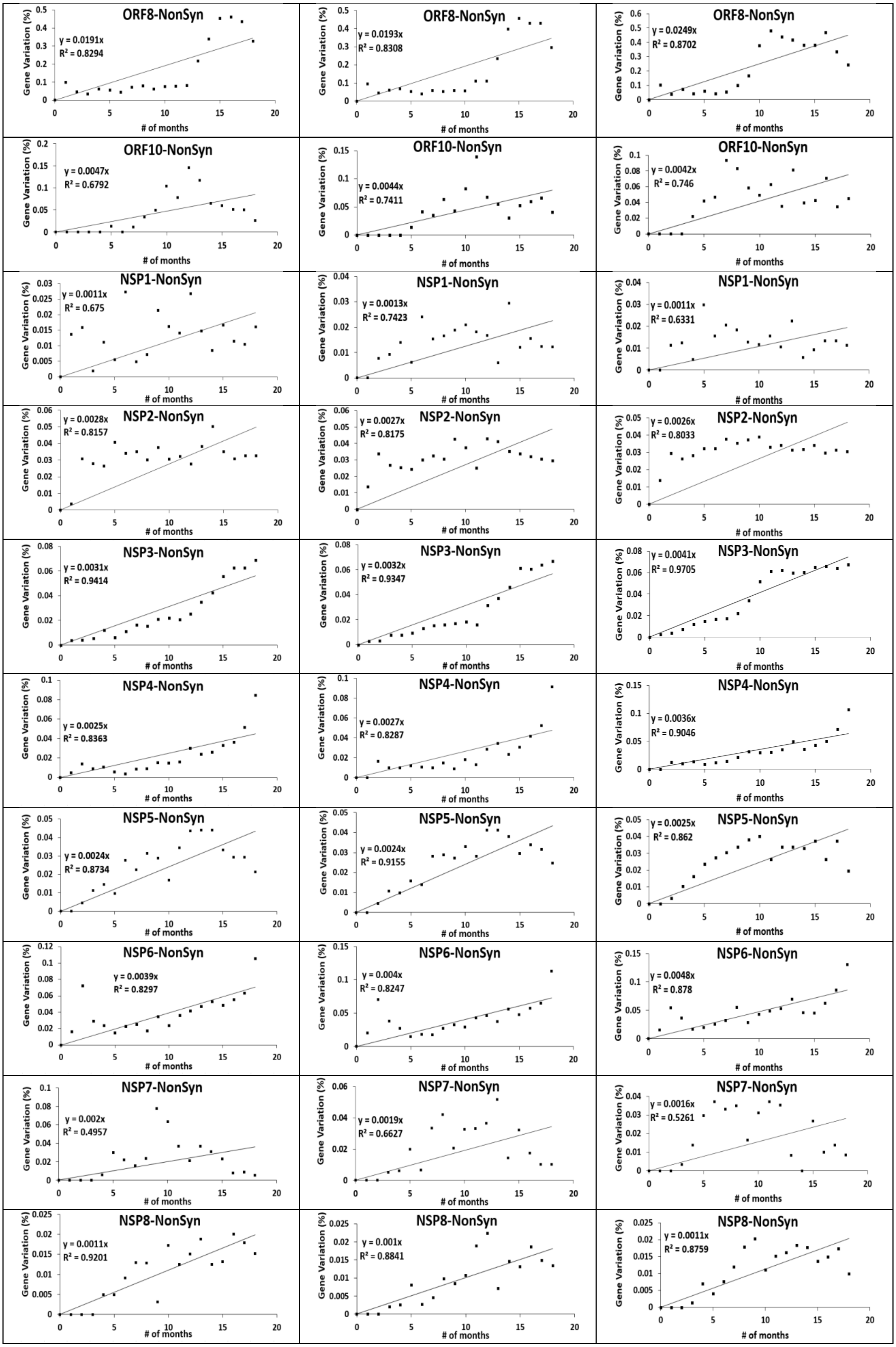

ORF8-NonSyn
y = 0.0191x
R² = 0.8294
ORF8-NonSyn
y = 0.0193x
R² = 0.8308
ORF8-NonSyn
y = 0.0249x
R² = 0.8702
ORF10-NonSyn
y = 0.0047x
R² = 0.6792
ORF10-NonSyn
y = 0.0044x
R² = 0.7411
ORF10-NonSyn
y = 0.0042x
R² = 0.746
NSP1-NonSyn
y = 0.0011x
R² = 0.675
NSP1-NonSyn
y = 0.0013x
R² = 0.7423
NSP1-NonSyn
y = 0.0011x
R² = 0.6331
NSP2-NonSyn
y = 0.0028x
R² = 0.8157
NSP2-NonSyn
y = 0.0027x
R² = 0.8175
NSP2-NonSyn
y = 0.0026x
R² = 0.8033
NSP3-NonSyn
y = 0.0031x
R² = 0.9414
NSP3-NonSyn
y = 0.0032x
R² = 0.9347
NSP3-NonSyn
y = 0.0041x
R² = 0.9705
NSP4-NonSyn
y = 0.0025x
R² = 0.8363
NSP4-NonSyn
y = 0.0027x
R² = 0.8287
NSP4-NonSyn
y = 0.0036x
R² = 0.9046
NSP5-NonSyn
y = 0.0024x
R² = 0.8734
NSP5-NonSyn
y = 0.0024x
R² = 0.9155
NSP5-NonSyn
y = 0.0025x
R² = 0.862
NSP6-NonSyn
y = 0.0039x
R² = 0.8297
NSP6-NonSyn
y = 0.004x
R² = 0.8247
NSP6-NonSyn
y = 0.0048x
R² = 0.878
NSP7-NonSyn
y = 0.002x
R² = 0.4957
NSP7-NonSyn
y = 0.0019x
R² = 0.6627
NSP7-NonSyn
y = 0.0016x
R² = 0.5261
NSP8-NonSyn
y = 0.0011x
R² = 0.9201
NSP8-NonSyn
y = 0.001x
R² = 0.8841
NSP8-NonSyn
y = 0.0011x
R² = 0.8759
Gene Variation (%)
# of months

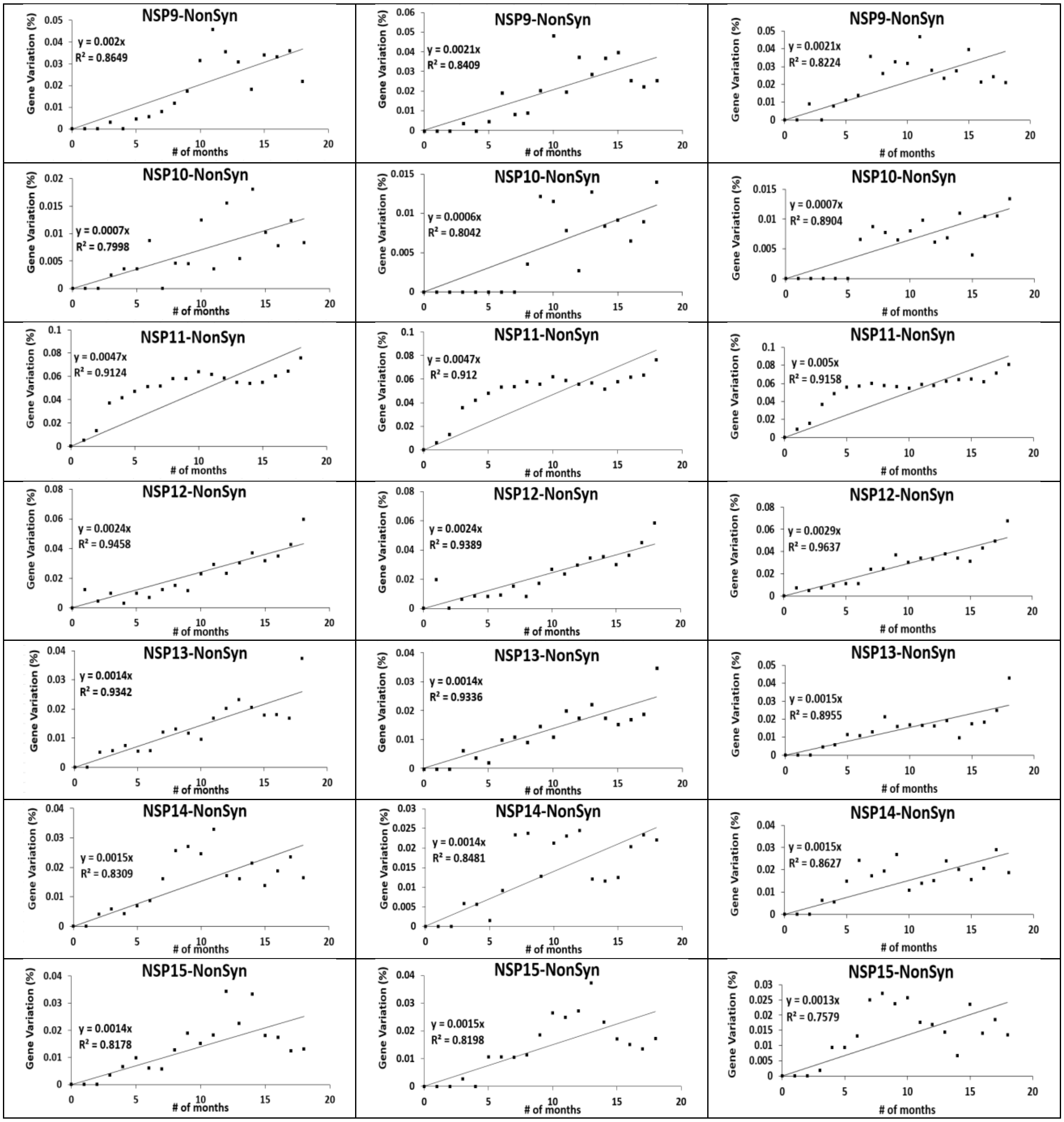

Figure S5. The percent Non-Synonymous codon variation of the proteome, each coding major/accessory protein and Nsp1-15 proteins over evolution time for each individual dataset.

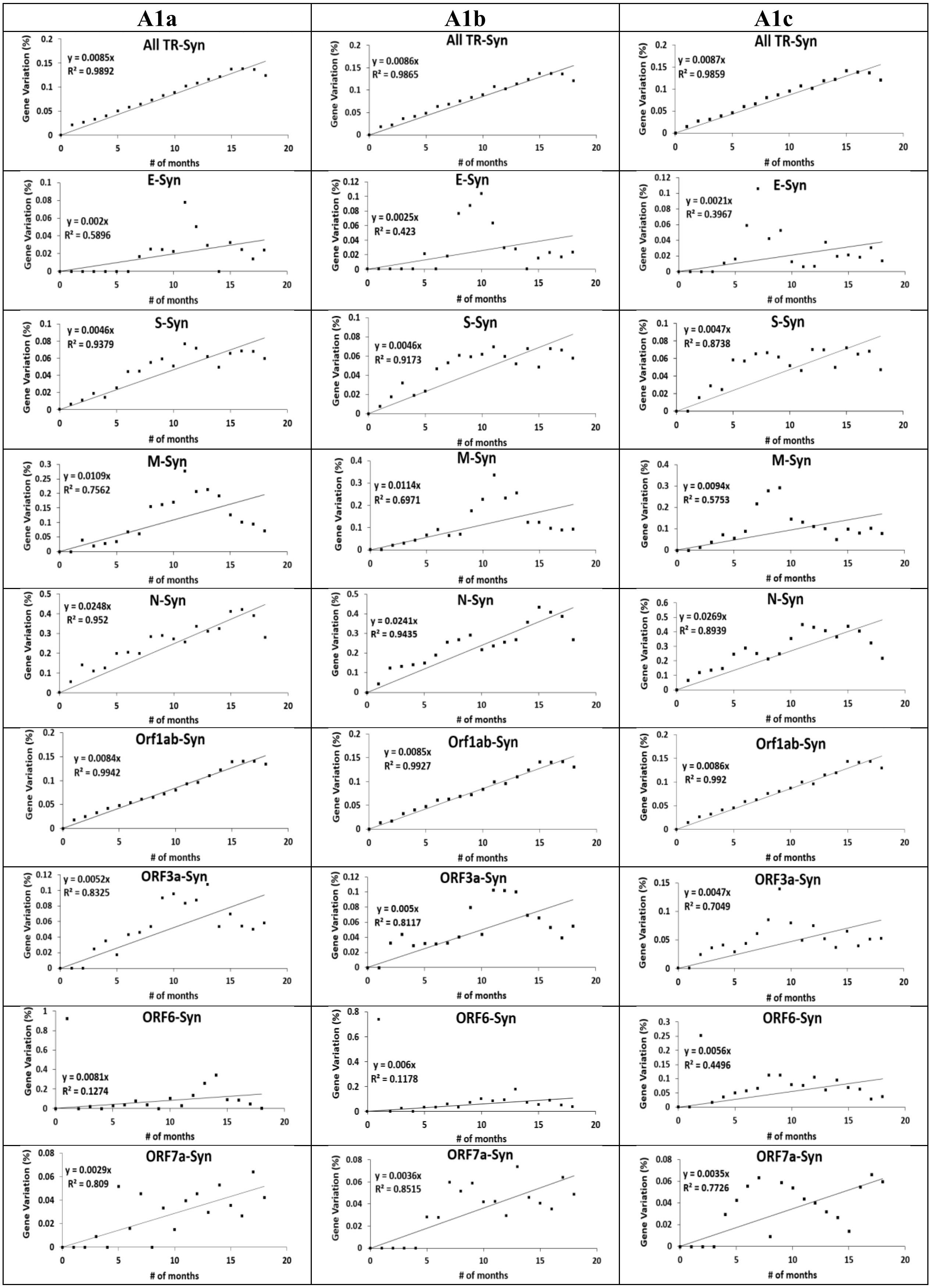
A1a
A1b
A1c
All TR-Syn
y = 0.0085x
R² = 0.9892
All TR-Syn
y = 0.0086x
R² = 0.9865
All TR-Syn
y = 0.0087x
R² = 0.9859
E-Syn
y = 0.002x
R² = 0.5896
E-Syn
y = 0.0025x
R² = 0.423
E-Syn
y = 0.0021x
R² = 0.3967
S-Syn
y = 0.0046x
R² = 0.9379
S-Syn
y = 0.0046x
R² = 0.9173
S-Syn
y = 0.0047x
R² = 0.8738
M-Syn
y = 0.0109x
R² = 0.7562
M-Syn
y = 0.0114x
R² = 0.6971
M-Syn
y = 0.0094x
R² = 0.5753
N-Syn
y = 0.0248x
R² = 0.952
N-Syn
y = 0.0241x
R² = 0.9435
N-Syn
y = 0.0269x
R² = 0.8939
Orf1ab-Syn
y = 0.0084x
R² = 0.9942
Orf1ab-Syn
y = 0.0085x
R² = 0.9927
Orf1ab-Syn
y = 0.0086x
R² = 0.992
ORF3a-Syn
y = 0.0052x
R² = 0.8325
ORF3a-Syn
y = 0.005x
R² = 0.8117
ORF3a-Syn
y = 0.0047x
R² = 0.7049
ORF6-Syn
y = 0.0081x
R² = 0.1274
ORF6-Syn
y = 0.006x
R² = 0.1178
ORF6-Syn
y = 0.0056x
R² = 0.4496
ORF7a-Syn
y = 0.0029x
R² = 0.809
ORF7a-Syn
y = 0.0036x
R² = 0.8515
ORF7a-Syn
y = 0.0035x
R² = 0.7726
Gene Variation (%)
# of months

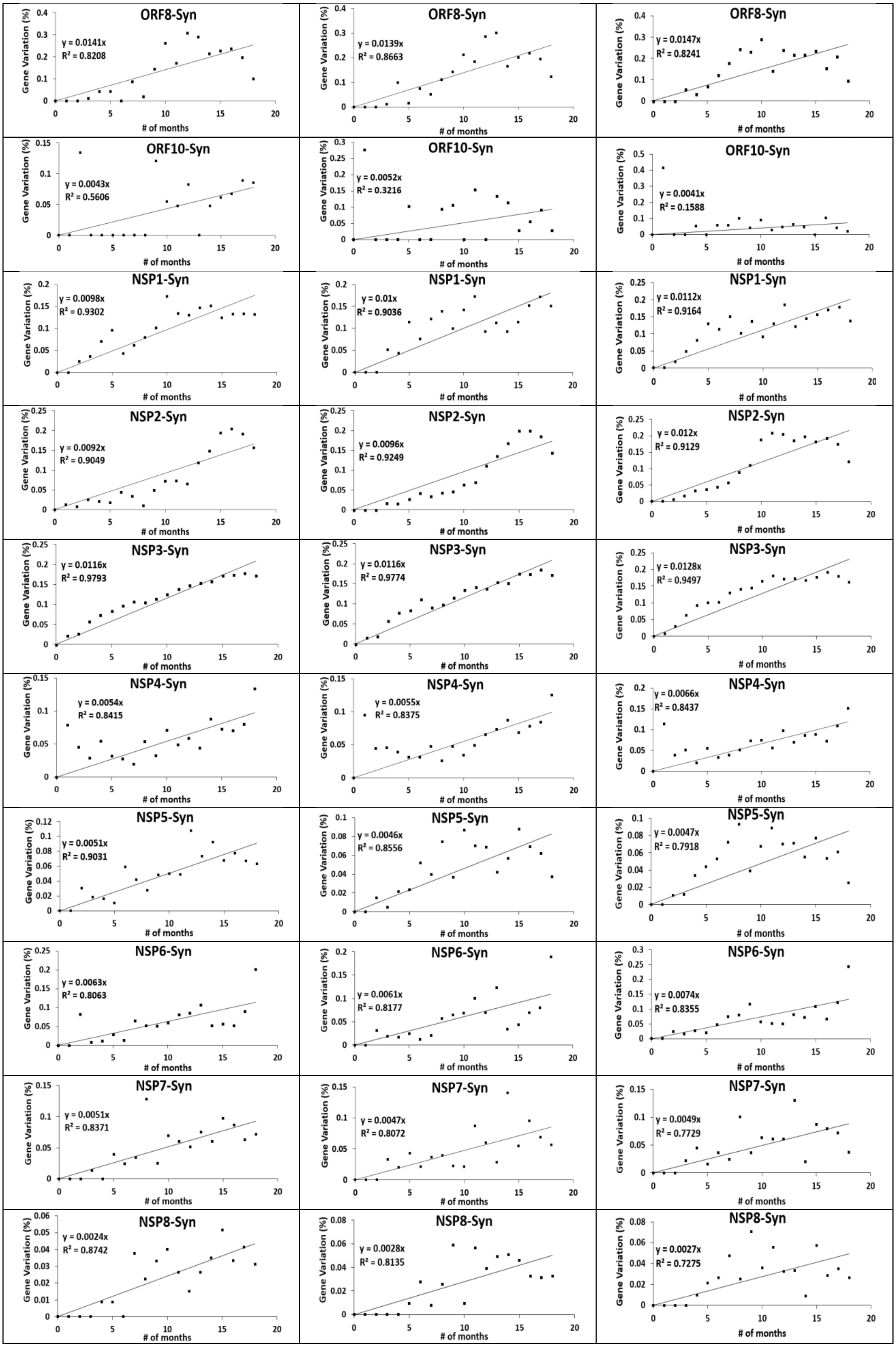
ORF8-Syn
y = 0.0141x
R² = 0.8208
ORF8-Syn
y = 0.0139x
R² = 0.8663
ORF8-Syn
y = 0.0147x
R² = 0.8241
ORF10-Syn
y = 0.0043x
R² = 0.5606
ORF10-Syn
y = 0.0052x
R² = 0.3216
ORF10-Syn
y = 0.0041x
R² = 0.1588
NSP1-Syn
y = 0.0098x
R² = 0.9302
NSP1-Syn
y = 0.01x
R² = 0.9036
NSP1-Syn
y = 0.0112x
R² = 0.9164
NSP2-Syn
y = 0.0092x
R² = 0.9049
NSP2-Syn
y = 0.0096x
R² = 0.9249
NSP2-Syn
y = 0.012x
R² = 0.9129
NSP3-Syn
y = 0.0116x
R² = 0.9793
NSP3-Syn
y = 0.0116x
R² = 0.9774
NSP3-Syn
y = 0.0128x
R² = 0.9497
NSP4-Syn
y = 0.0054x
R² = 0.8415
NSP4-Syn
y = 0.0055x
R² = 0.8375
NSP4-Syn
y = 0.0066x
R² = 0.8437
NSP5-Syn
y = 0.0051x
R² = 0.9031
NSP5-Syn
y = 0.0046x
R² = 0.8556
NSP5-Syn
y = 0.0047x
R² = 0.7918
NSP6-Syn
y = 0.0063x
R² = 0.8063
NSP6-Syn
y = 0.0061x
R² = 0.8177
NSP6-Syn
y = 0.0074x
R² = 0.8355
NSP7-Syn
y = 0.0051x
R² = 0.8371
NSP7-Syn
y = 0.0047x
R² = 0.8072
NSP7-Syn
y = 0.0049x
R² = 0.7729
NSP8-Syn
y = 0.0024x
R² = 0.8742
NSP8-Syn
y = 0.0028x
R² = 0.8135
NSP8-Syn
y = 0.0027x
R² = 0.7275
Gene Variation (%)
# of months

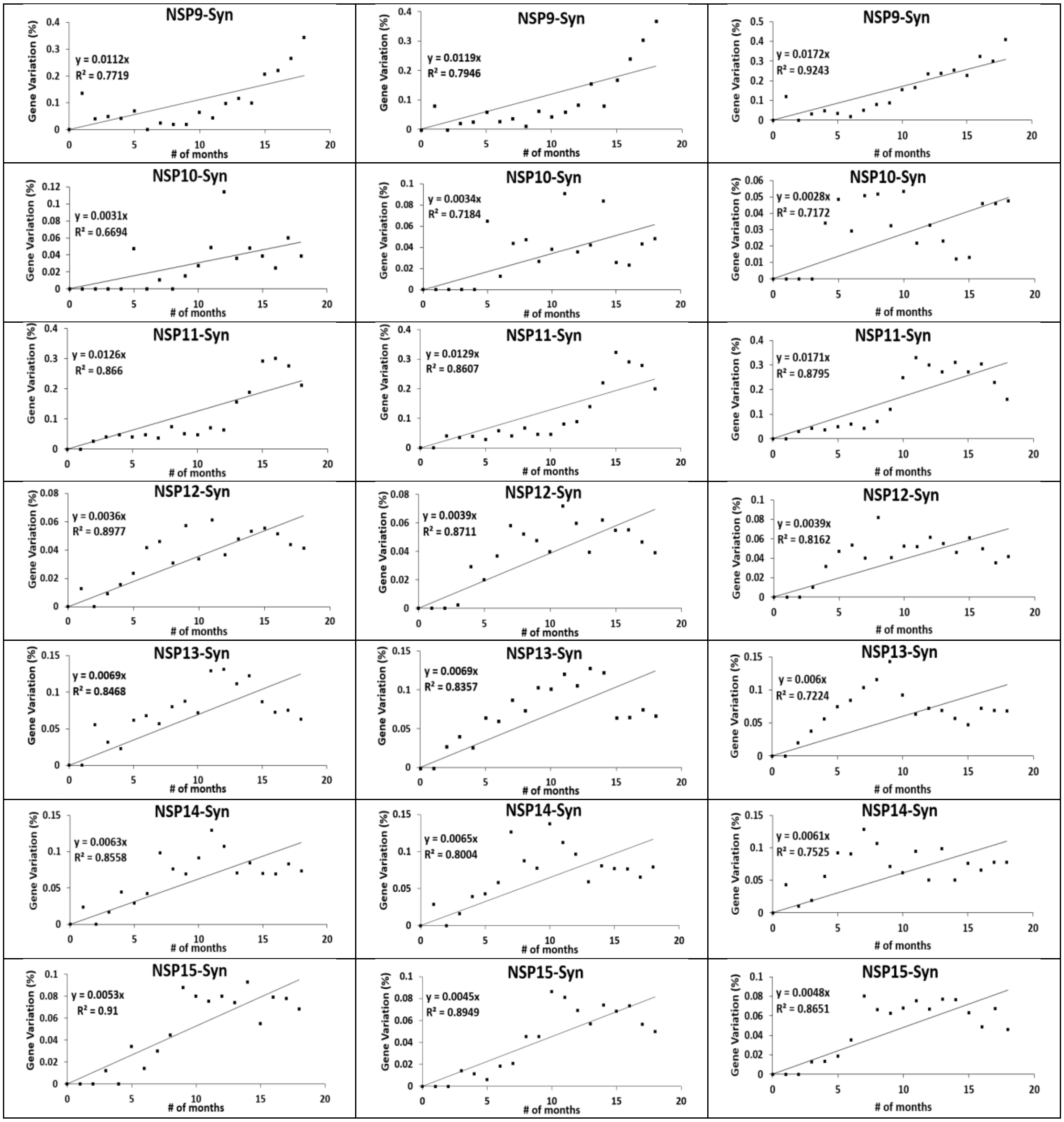

Figure S6. The percent Synonymous NT substitutions for All-TR, each coding major/accessory gene and Nsp1-15 genes over evolution time for each individual dataset.

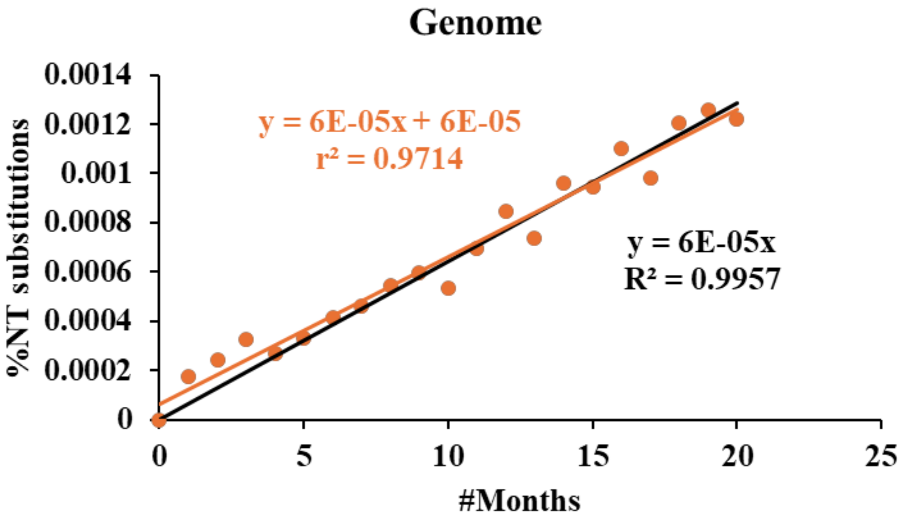

Figure S7. The trendlines of the SARS-COV-2 genomic substitution rate fitted through the origin (R^2, coefficient of determination, black) and when not fitted through the origin (r^2, correlation coefficient, orange).
